# *Lindera* cyclopentenedione intermediates from the roots of *Lindera aggregata*†

**DOI:** 10.1039/c8ra03094d

**Published:** 2018-05-16

**Authors:** Lin Chen, Bo Liu, Jun-Jie Deng, Jun-Sheng Zhang, Wei Li, Abrar Ahmed, Sheng Yin, Gui-Hua Tang

**Affiliations:** School of Pharmaceutical Sciences, Sun Yat-sen University Guangzhou Guangdong 510006 China tanggh5@mail.sysu.edu.cn +86-20-39943043 +86-20-39943043; The Second Clinical Medical College, Guangzhou University of Chinese Medicine Guangzhou 510006 China

## Abstract

Chromatographic fractionation of the roots of *Lindera aggregata* has led to the isolation of three new monomers of *Lindera* cyclopentenedione derivatives (1–3), a pair of new enantiomers of bi-linderone derivatives (4a/4b), and six known *Lindera* cyclopentenediones (5–8 and 9a/9b). Their structures were determined by NMR and MS data. The absolute configurations of the new bi-linderone derivative enantiomers (4a/4b) were determined by ECD calculation. (±)-Lindepentone A (1) presents the novel skeleton of 3,5-dioxocyclopent-1-enecarboxylate. Lindoxepines A (2) and B (3) present an unprecedented oxepine-2,5-dione derivative skeleton, which may be enlightening for the *in vivo* biosynthesis of the monomers of *Lindera* cyclopentenediones. A possible biosynthetic pathway for 1 and a plausible biosynthetic pathway from stilbenes to *Lindera* cyclopentenediones *via* the key intermediates 2 and 3 were postulated. The inhibitory activity of these compounds against three microorganisms was also evaluated.

## Abstract 2

*Lindera* cyclopentenediones together with their dimers and novel biosynthetic intermediates were isolated from *Lindera aggregata*.

## Graphical abstract

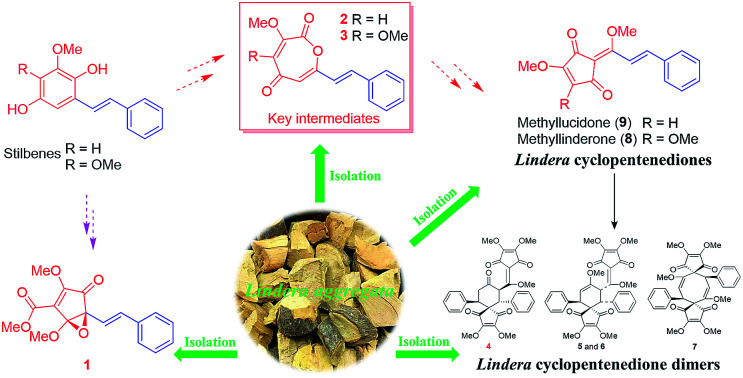

## Introduction

**Fig. 1:**
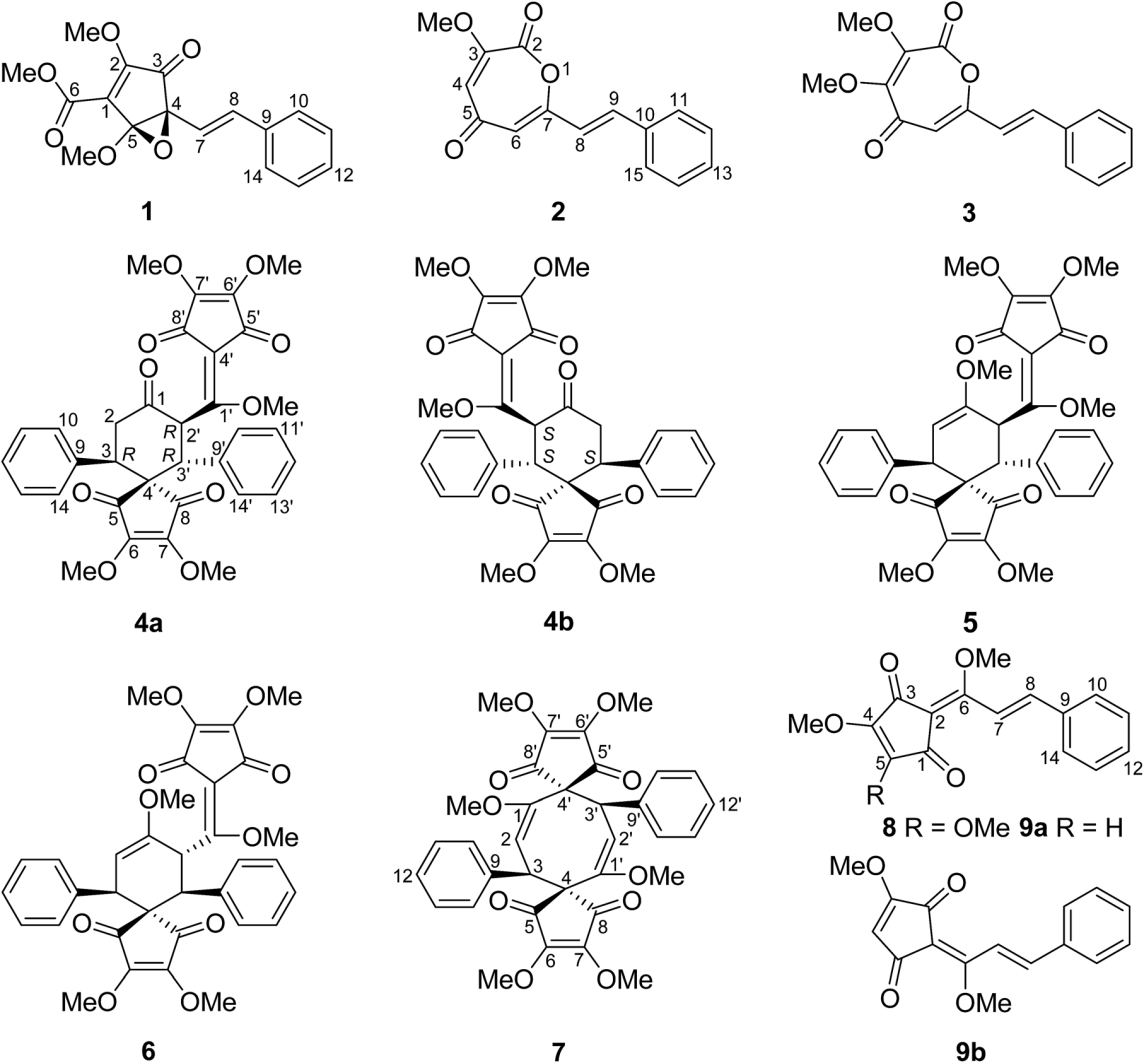
Structures of the compounds isolated from *Lindera aggregata*.

The dry roots of *Lindera aggregata* (Sims) Kosterm. (*L. strychnifolia*) (Lauraceae), Radix Linderae (Chinese name “Wuyao” and Japanese name “Tendai-Uyaku”) have been used for many years as a well-known traditional medicine for the treatment of several diseases such as rheumatism, chest and abdomen pain, regurgitation, and frequent urination.^1,2^ In China, these evergreen shrubs or small trees of *L. aggregata* are mainly distributed in Anhui, Fujian, Guangdong, Zhejiang, and other southern provinces.^3^ So far, various types of natural products such as sesquiterpenoids, alkaloids, flavonoids, and lucidones have been isolated from the plant of *L. aggregata*, some of which exhibit various types of bioactivity such as antioxidation, cytotoxicity, protection against postischemic myocardial dysfunction, and the improvement of insulin sensitivity.^4,5^ Lucidones, a rare group of cyclopentenediones currently reported only in five species of the genus *Lindera* (*L. aggregata*, *L. lucida*, *L. erythrocarpa*, *L. oxyphylla*, and *L. pipericarpa*), mainly consist of several monomers (*e.g.* linderone, methyllinderone, lucidone, and methyllucidone) and dimers (*e.g.* linderaspirone A and bi-linderone) with various biological activities such as antimicrobial activity.^5–7^ Herein, we use the term “*Lindera* cyclopentenediones” to represent this group of structures. The intriguing structures and diverse activity of *Lindera* cyclopentenedione dimers have attracted broad interest from synthetic chemists. To date, several research groups have totally synthesized linderaspirone A and bi-linderone through different synthetic methods, in which the preparation of the key precursor methyllinderone is a necessary route.^8–10^ However, the biosynthesis of the monomers of *Lindera* cyclopentenediones *in vivo* has not been reported. The novel structures of lindoxepines A and B (2 and 3) discovered from *L. aggregata* may shed some light on this issue (Fig. 1). In the present study, besides the two novel oxepine-2,5-dione derivatives (2 and 3), a novel 3,5-dioxocyclopent-1-enecarboxylate derivative (1) and a pair of enantiomers of bi-linderone derivatives (4a/4b), six known *Lindera* cyclopentenediones (5–8 and 9a/9b) were obtained from this plant during our ongoing search for structurally diverse and biologically significant compounds from medical plants.^11–17^ A possible biosynthetic pathway for 1 and a plausible biosynthetic pathway from stilbenes to *Lindera* cyclopentenediones *via* the key intermediates 2 and 3 were postulated. The inhibitory effects of these compounds on three microorganisms were also evaluated using the microplate reader method.

## Results and discussion

**Fig. 2:**
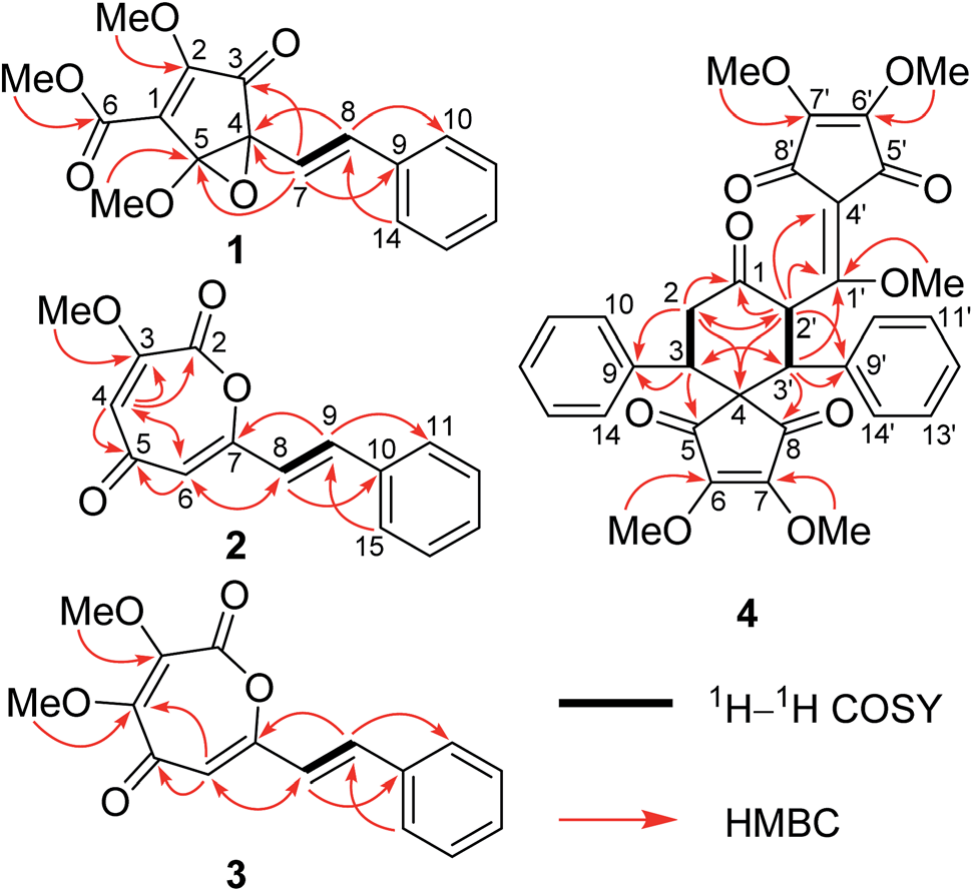
Key ^1^H–^1^H COSY and HMBC correlations of compounds 1–4.

HRESIMS of 1 displayed a pseudomolecular ion peak at *m*/*z* 317.1011 [M + H]^+^ (calcd 317.1020), allowing the molecular formula C_17_H_16_O_6_ with ten degrees of unsaturation to be assigned. The IR spectrum exhibited absorptions for unsaturated ketone (1737 cm^−1^) and phenyl (1636, 1497, and 1449 cm^−1^) moieties. The ^1^H and ^13^C NMR data (Table 1) displayed signals for three methoxy groups [*δ*_H_ 4.26, 3.80, and 3.59 (each 3H, s); *δ*_C_ 60.2, 56.1, and 53.1 (each CH_3_)], a mono-substituted phenyl [*δ*_H_ 7.44 (2H, d, *J* = 7.5 Hz), 7.35 (2H, t, *J* = 7.5 Hz), and 7.27 (1H, t, *J* = 7.5 Hz); *δ*_C_ 138.0 (C), 129.7 (CH × 2), 128.9 (CH), and 127.6 (CH × 2)], a *trans*-double bond [*δ*_H_ 6.87 (1H, d, *J* = 16.1 Hz) and 6.33 (1H, d, *J* = 16.1 Hz); *δ*_C_ 133.0 (CH) and 127.9 (CH)], a tetrasubstituted double bond [*δ*_C_ 158.7 (C) and 136.6 (C)], a conjugated ketone [*δ*_C_ 196.0 (C)], one ester carbonyl group [*δ*_C_ 170.8 (C)], and two oxygenated quaternary carbons [*δ*_C_ 89.7 (C) and 83.3 (C)]. This collective information suggests that 1 is analogous to the cyclopentenedione derivatives, methyllinderone (8)^18^ and methyllucidone (9),^19^ co-isolated in the current study. The significant difference was that the formation of cyclopentenone had changed due to the presence of the ester carbonyl group and the two oxygenated quaternary carbons. The HMBC correlations of H-7 to C-9, H-8 to C-10 (14), and H-10 (14) to C-8 confirmed that the *trans*-double bond was linked to the mono-substituted phenyl to form an (*E*)-styryl moiety, which was located at the oxygenated quaternary carbon (C-4) by the HMBC correlations of H-8 to C-4 and H-7 to C-3, C-4, and C-5 (Fig. 2). The determination of the cyclopentenone characteristic of a conjugated system (ketone–tetrasubstituted double bond–ester carboxyl group, C-3–Δ^1^–C-6) with a methoxy group at C-2 and a methoxy group at C-6 was confirmed by the comparison of these carbon chemical shifts with those of the substructure of the synthesis, (4*R*,5*R*)-ethyl-4-(allyloxy)-2-methoxy-4-(4-methoxyphenyl)-3-oxo-5-phenylcyclopent-1-enecarboxylate,^20^ and the HMBC correlations of the methoxy protons (*δ*_H_ 4.26) to C-2 and the methoxy protons (*δ*_H_ 3.80) to C-6. As nine of the ten degrees of unsaturation were accounted for by a phenyl group, two double bonds, a ketone group, one ester carbonyl group, and a pentyl ring, the remaining one degree of unsaturation suggested that an epoxy ring was present between the two oxygenated quaternary carbons (C-4 and C-5). The HMBC correlation of the methoxy protons (*δ*_H_ 3.59) to C-5 determined that this methoxy group was located at the ketal carbon (C-5). Thus, the gross structure of 1 was deduced as shown, and represented a novel 3,5-dioxocyclopent-1-enecarboxylate derivative.

**Table 1:** ^1^H NMR (400 MHz) and ^13^C NMR (100 MHz) spectroscopic data of 1–3 (*δ* in ppm)

Position	1^a^		Position	2^b^		3^b^	
	*δ*_H_, multi (*J* in Hz)	*δ*_C_, type		*δ*_H_, multi (*J* in Hz)	*δ*_C_, type	*δ*_H_, multi (*J* in Hz)	*δ*_C_, type
1		136.6, C	2		151.9, C		144.6, C
2		158.7, C	3		160.7, C		160.8, C
3		196.0, C	4	7.05, d (2.3)	119.3, CH		149.9, C
4		83.3, C	5		179.7, C		176.9, C
5		89.7, C	6	6.37, d (2.3)	115.2, CH	6.43, s	114.7, CH
6		170.8, C	7		162.5, C		161.3, C
7	6.33, d (16.1)	127.9, CH	8	6.72, d (16.0)	118.9, CH	6.69, d (16.1)	118.7, CH
8	6.87, d (16.1)	133.0, CH	9	7.561, d (16.0)	138.0, CH	7.50, d (16.1)	138.0, CH
9		138.0, C	10		134.8, C		134.8, C
10/14	7.44, d (7.5)	127.6, CH	11/15	7.562, dd (7.6, 1.8)	127.9, CH	7.55, dd (8.0, 1.8)	127.9, CH
11/13	7.35, t (7.5)	129.7, CH	12/14	7.38–7.43, m	129.2, CH	7.38–7.43, m	129.2, CH
12	7.27, t (7.5)	128.9, CH	13	7.38–7.43, m	130.2, CH	7.38–7.43, m	130.2, CH
2-OMe	4.26, s	60.2, CH_3_	4-OMe			4.03, s	61.3, CH_3_
5-OMe	3.59, s	56.1, CH_3_	3-OMe	4.00, s	53.6, CH_3_	4.01, s	53.3, CH_3_
6-OMe	3.80, s	53.1, CH_3_					

a In CD_3_OD.

b In CDCl_3_.

The relative configuration of 1 was determined by NOESY experiment. The observed cross-peaks of H-7 and H-8 with the protons of 5-OCH_3_ suggested that the styryl moiety and 5-OCH_3_ were cofacial and were arbitrarily assigned as the α-orientation. This compound might be a racemic mixture because its specific rotation was zero. Therefore, compound 1 was named as (±)-lindepentone A.

**Scheme 1:**
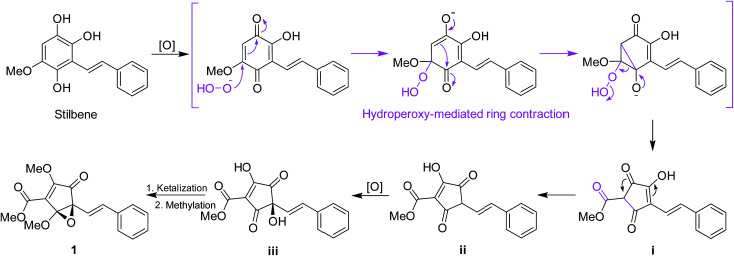
Plausible biosynthetic pathway for 1.

Structurally, for the biosynthesis of the novel skeleton of 3,5-dioxocyclopent-1-enecarboxylate, a putative biogenetic pathway to 1 was proposed in Scheme 1 from a stilbene precursor, 2,5,6-trihydroxy-3-methoxystilbene, which could be modified through oxidation followed by hydroperoxy-mediated ring contraction^21^ to form the intermediate i. Enol-isomerization could convert i to ii which can be further oxidized to iii, which could be intraketally ketalized to form the epoxy ring, and finally methylated to give 1.

Lindoxepine A (2) has the molecular formula C_15_H_12_O_4_ with ten degrees of unsaturation as established by the HRESIMS (*m*/*z* 257.0800 [M + H]^+^, calcd 257.0808) and ^13^C NMR data. The ^1^H and ^13^C NMR data of 2 (Table 1) exhibited signals for a methoxy group [*δ*_H_ 4.00 (3H, s); *δ*_C_ 53.6 (CH_3_)] and one (*E*)-styryl moiety [*δ*_H_ 7.561 and 6.72 (each 1H, d, *J* = 16.0 Hz, *E*-double bond protons), 7.562 (2H, dd, *J* = 7.6, 1.8 Hz, Ar–H), and 7.38–7.43 (3H, m, Ar–H); *δ*_C_ 138.0 and 118.9 (each CH, double bond carbons), 134.8 and 130.2 (each C, Ar–C), and 129.2 and 127.9 (each CH × 2, Ar–C)] as in 1, which was further supported by the HSQC and HMBC correlations (Fig. 2). The remaining resonances in the ^1^H and ^13^C NMR data of 2 (Table 1) were very similar to oxepine-2,5-dione derivatives.^22^ Analysis of its 2D NMR correlations (Fig. 2) and degrees of unsaturation further confirmed the presence of an oxepine-2,5-dione moiety with two substituents. The HMBC correlations of H-8 to C-6, H-9 to C-7, and H-6 to C-8 determined that the (*E*)-styryl group was located at C-7, and the methoxy group was placed at C-3 by the HMBC correlation of the protons to C-3. Thus, compound 2 was elucidated as shown, and represented a novel oxepine-2,5-dione derivative firstly isolated as a natural product.

The molecular formula of compound 3 was determined to be C_16_H_14_O_5_ by the HRESIMS and ^13^C NMR data. Comparison of the 1D NMR data (Table 1) and MS information of 3 with those of 2 suggested that 3 had an additional methoxy group at the down-shifted carbon C-4 [*δ*_C_ 149.9 (C) in 3; *δ*_C_ 119.3 (CH) in 2], which was further supported by the HMBC correlations of the protons (*δ*_H_ 4.03) of OCH_3_ to C-4 (Fig. 2). Thus, compound 3 was elucidated as lindoxepine B.

**Scheme 2:**
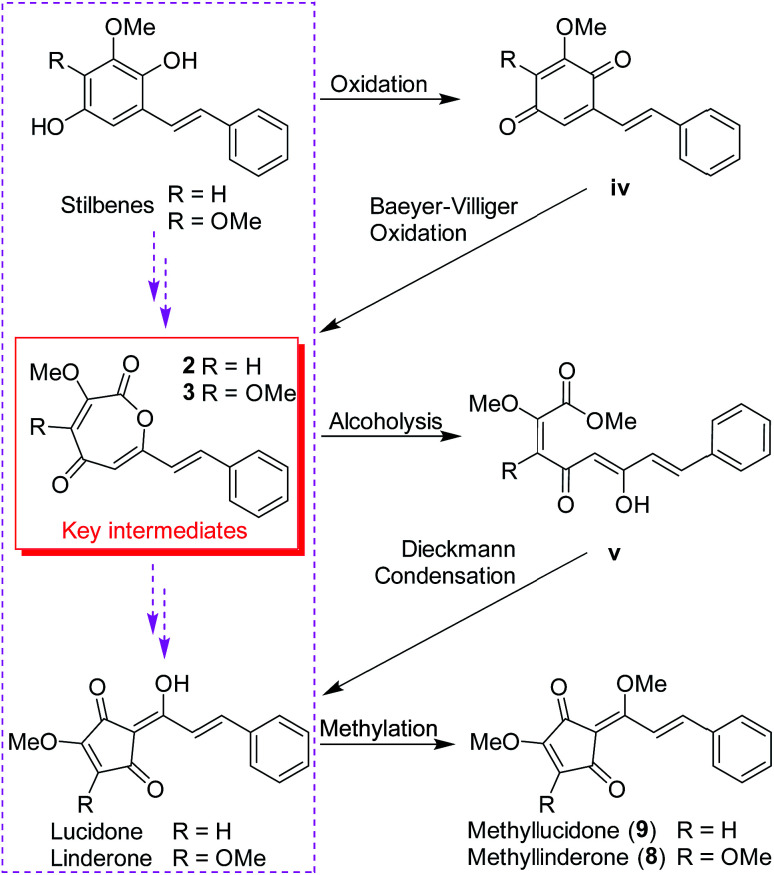
The plausible biosynthetic pathway from stilbenes to *Lindera* cyclopentenediones *via* the key intermediates (2 and 3) isolated from *Lindera aggregata*.

Lindoxepines A (2) and B (3) present an unprecedented oxepine-2,5-dione derivative skeleton, which may be enlightening for the *in vivo* biosynthesis of the monomers of *Lindera* cyclopentenediones. As shown in Scheme 2, the stilbene natural products may be oxidized to form *p*-benzoquinone derivatives (iv), followed by Baeyer–Villiger oxidation to obtain the key intermediates 2 and 3. Alcoholysis of 2 and 3 yielded v, which was then converted into the known *Lindera* cyclopentenediones, lucidone and linderone,^5^ by Dieckmann condensation. Moreover, the co-isolated methyllinderone (8)^18^ and methyllucidone (9)^19^ should be the methylated products of linderone and lucidone, respectively. Thus, lindoxepines A (2) and B (3) may be the key intermediates for the synthesis of *Lindera* cyclopentenediones *in vivo*.

Compound 4 was isolated as a white amorphous powder, and its molecular formula was established as C_33_H_30_O_10_ by the HRESIMS and ^13^C NMR data. The ^1^H and ^13^C NMR signals (Table 1) of 4 bore a high resemblance to those of *epi*-bi-linderone (5),^8^ a synthesized compound firstly isolated as a natural product in the current study, except that the characteristic signals for the enol ether moiety [*δ*_H_ 4.83 (1H, d, *J* = 5.1 Hz, H-2) and 3.58 (3H, s, 1-OMe); *δ*_C_ 155.2 (C, C-1), 95.6 (CH, C-2), and 55.0 (CH_3_, 1-OMe)] in 5 were not observed in 4 but signals were shown for an isolated ketone carbonyl (*δ*_C_ 206.4) and a methylene [*δ*_H_ 3.54 (1H, dd, *J* = 17.0 and 14.0 Hz) and 2.76 (1H, dd, *J* = 17.0 and 2.9 Hz); *δ*_C_ 41.5 (CH_2_)] in its 1D NMR spectrum. This information suggested that the enol ether at Δ^1^ was hydrolyzed to form a ketone at C-1, which was supported by the ^1^H–^1^H COSY correlations of H_2_-2 with H-3 and the HMBC correlations of H-2 and H-2′ to the ketone (C-1) (Fig. 2). Thus, the gross structure of 4 was elucidated as shown in Fig. 2.

**Fig. 3:**
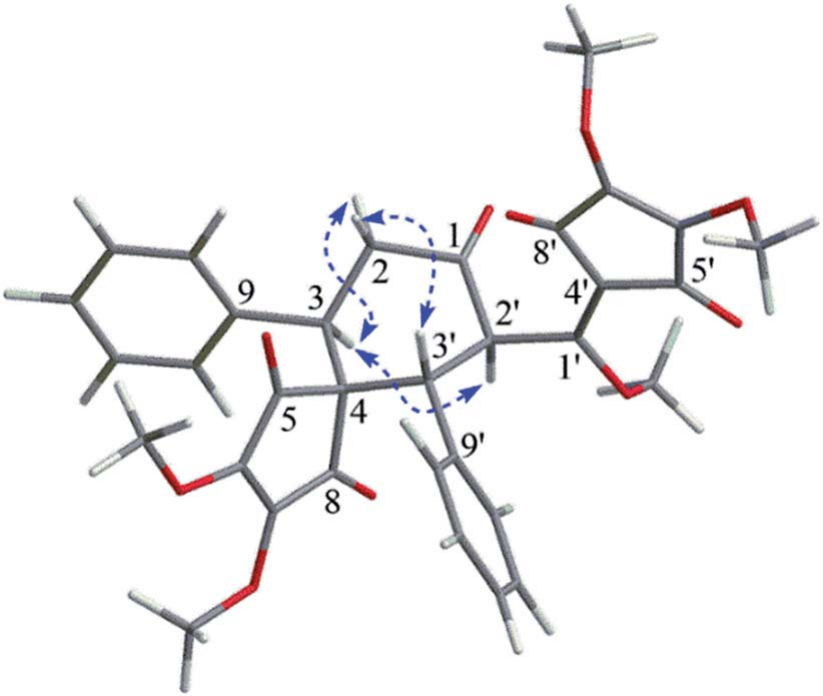
Key NOE correlations of compound 4.

The relative configuration of 4 was determined by the analysis of its coupling constant and NOE correlations. The big coupling constant (*J*_2′/3′_ = 13.3 Hz) of H-2′ and H-3′ in the ^1^H NMR spectrum suggested that H-2′ and H-3′ had a *trans*-configuration, and H-2′ and H-3′ were arbitrarily assigned as the α- and β-orientation, respectively. The NOE correlation of H-3/H-2′ in the NOESY spectrum indicated that H-3 was in the α-orientation (Fig. 3).

**Fig. 4:**
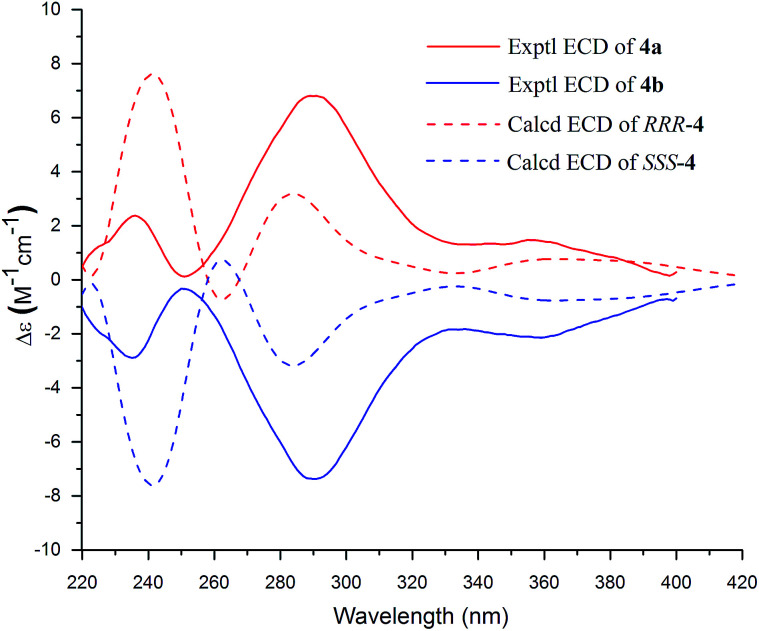
Experimental ECD spectra of 4a and 4b and TDDFT calculated ECD spectra for *RRR*-4 and *SSS*-4.

Compound 4 was a racemic mixture because its specific rotation approached zero and it had no cotton effect in its ECD spectrum. Subsequently, 4 was subjected to HPLC with a chiral column to obtain the enantiomers 4a and 4b, which had opposite specific rotations ([*α*]^25^_D_ = +274.4 for 4a and [*α*]^25^_D_ = −286.8 for 4b) and mirror image-like ECD curves (Fig. 4). The experimental ECD spectra of 4a and 4b were compared with the calculated ECD spectra of (*RRR*)-4 or (*SSS*)-4 by the TDDFT method to determine the absolute configurations of the enantiomers. In Fig. 4, the experimental ECD spectrum of 4a matched the calculated ECD curve for (*RRR*)-4, indicating that 4a possessed the same absolute configuration as (*RRR*)-4. Meanwhile, the absolute configuration of 4b was deduced to be the same as that of (*SSS*)-4 on the basis of their matched ECD spectra. Thus, the enantiomers 4a and 4b were determined as shown and named (+)-demethoxy-*epi*-bi-linderone (4a) and (−)-demethoxy-*epi*-bi-linderone (4b), respectively.

The known compounds were identified as *epi*-bi-linderone (5),^8^ bi-linderone (6),^6^ linderaspirone A (7),^7^ methyllinderone (8),^18^ and methyllucidone (9) (a pair of *cis*–*trans* isomers, 9a and 9b)^19^ by comparing their NMR data with those reported in the literature.

Compounds 1–9 from *Lindera aggregata* were tested for their antimicrobial activity against the bacteria *Bacillus subtilis* and the fungi *Candida albicans* and *Aspergillus niger* by the microplate reader method. It is shown in Table 3 that compounds 1–4a/4b and 6–9 exhibited weak inhibitory activity against *C. albicans* (MIC values: 25 μg mL^−1^ for 6 and 7 and 50 μg mL^−1^ for the others) and compound 7 had a weak effect on *A. niger* with a MIC value of 50 μg mL^−1^. All of the compounds were inactive towards the bacteria *B. subtilis* (MIC values: >50 μg mL^−1^) and compound 5 and compounds 1–6, 8, and 9 were inactive towards the fungi *C. albicans* and *A. niger*, respectively.

**Table 2:** The ^1^H (400 MHz) and ^13^C NMR (100 MHz) data of 4 in CDCl_3_ (*δ* in ppm)

Position	*δ*_H_, multi (*J* in Hz)	*δ*_C_, type	Position	*δ*_H_, multi (*J* in Hz)	*δ*_C_, type
1		206.4, C	1′		169.2, C
2	*β* 3.54, dd (17.0, 14.0); *α* 2.76, dd (17.0, 2.9)	41.5, CH_2_	2′	6.39, d (13.3)	52.8, CH
3	3.97, dd (14.0, 2.9)	41.2, CH	3′	4.23, d (13.3)	45.0, CH
4		58.7, C	4′		110.7, C
5, 8		195.9 and 195.7, each C	5′, 8′		187.5 and 183.3, each C
6, 7		153.2 and 153.1, each C	6′, 7′		148.7 and 146.9, each C
9		137.1, C	9′		135.7, C
10/14, 11/13, 12	7.02–7.24, m, overlapped	128.8 × 2, 128.6 × 2, and 127.9, each CH	10′/14′, 11′/13′, 12′	7.02–7.24, m, overlapped	128.8 × 2, 128.4 × 2, and 127.8, each CH
6-, 7-OMe	3.74 and 3.66, each s	59.8 and 59.6, each CH_3_	6′-, 7′-OMe	4.20 and 4.12, each s	60.2 and 59.7, each CH_3_
			1′-OMe	3.65, s	65.8, CH_3_

**Table 3:** Antimicrobial activity of 1–9 (MIC in μg mL^−1^)

Compd.	*Bacillus subtilis*	*Candida albicans*	*Aspergillus niger*
1	>50	50	>50
2	>50	50	>50
3	>50	50	>50
4a	>50	50	>50
4b	>50	50	>50
5	>50	>50	>50
6	>50	25	>50
7	>50	25	50
8	>50	50	>50
9	>50	50	>50
Ampicillin^a^	<1	NT^b^	NT^b^
Amphotericin B^a^	NT^b^	<1	NT^b^

a Positive control.

b Not tested.

## Experimental section

### General experimental procedures

Optical rotations were measured on a Rudolph Autopol I automatic polarimeter, UV spectra on a Shimadzu UV-2450 spectrophotometer, and IR spectra on Bruker Tensor 37 infrared spectrophotometers. NMR spectra were measured on Bruker AM-400 spectrometers at 25 °C. ESIMS was measured on a Finnigan LCQ Deca instrument, and HRESIMS was performed on a Waters Micromass Q-TOF spectrometer. Silica gel (300–400 mesh, Qingdao Haiyang Chemical Co., Ltd.), reversed-phase C_18_ (RP-C_18_) (12 nm, S-50 μm, YMC Co., Ltd.), and Sephadex LH-20 gel (Amersham Biosciences) were used for column chromatography (CC). A Shimadzu LC-20 AT equipped with a SPD-M20A PDA detector was used for HPLC. A YMC-pack ODS-A column (250 × 10 mm, S-5 μm, 12 nm) and a Phenomenex Lux cellulose-2 chiral column (10 × 250 mm, 5 μm) were used for semi-preparative HPLC separation. All of the solvents used were of analytical grade (Guangzhou Chemical Reagents Company, Ltd.).

### Plant material

The roots of *L. aggregata* were collected in the Kunming, Yunnan Province, P. R. China, in November 2016, and were authenticated by one of the authors (G. H. Tang). A voucher specimen (accession number: YLAC-201611) has been deposited at the School of Pharmaceutical Sciences, Sun Yat-sen University.

### Extraction and isolation

The air-dried powder of the roots of *L. aggregata* (2.5 kg) was extracted with 95% EtOH (3 × 10 L) at rt to give 362 g of crude extract. The extract was suspended in H_2_O (2 L) and successively partitioned with petroleum ether (PE, 3 × 2 L), EtOAc (3 × 2 L), and *n*-BuOH (3 × 2 L) to yield three corresponding portions. The EtOAc extract (120 g) was subjected to silica gel CC (PE/EtOAc, 20 : 1 → 0 : 1) to afford Fr. I–Fr. IV. Fr. I (4.5 g) was subjected to silica gel CC (PE/CH_2_Cl_2_, 5 : 1 → 1 : 1) to get 8 (870 mg). Fr. III (3.2 g) was subjected to silica gel CC (PE/CH_2_Cl_2_, 5 : 1 → 0 : 1) to afford Fr. IIIa–Fr. IIIc. Fr. IIIb was loaded onto a Sephadex LH-20 column and eluted with MeOH to give Fr. IIIb1–Fr. IIIb3. Fr. IIIb2 was purified on a silica gel CC using PE/CH_2_Cl_2_, 10 : 1 → 0 : 1 to give 7 (6 mg). Fr. IIIb3 was subjected to silica gel CC (PE/acetone, 5 : 1 → 2 : 1) to give 1 (5.3 mg). Fr. IIIa was loaded onto a Sephadex LH-20 column and eluted with MeOH to give Fr. IIIa1–Fr. IIIa2, Fr. IIIa1 was subjected to silica gel CC (PE/EtOAc, 10 : 1 → 5 : 1) to afford Fr. IIIa1a–Fr. IIIa1b. Fr. IIIa1a was purified on a semi-preparative HPLC system equipped with a YMC column (MeOH/H_2_O, 7.5 : 2.5, 3 mL min^−1^), to give 5 (15 mg, *t*_R_ 7 min). Fr. IIIa1b was subjected to silica gel CC (PE/EtOAc, 10 : 1 → 1 : 1) to give 6 (4 mg). Compound 4 (12 mg) was purified by using HPLC (MeOH/H_2_O, 8 : 2, 3 mL min^−1^) from Fr. IIIa1b, and this was followed by HPLC with a Phenomenex Lux chiral column (MeOH/H_2_O, 100 : 0, 3 mL min^−1^) to obtain 4a (3.3 mg, *t*_R_ 15 min) and 4b (3.1 mg, *t*_R_ 19 min), respectively. Fr. IIIa2 was chromatographed over a RP-C_18_ column eluted with MeOH/H_2_O (7 : 3 → 10 : 0) to afford Fr. IIIa2a–Fr. IIIa2c. Fr. IIIa2a was loaded onto a Sephadex LH-20 column and eluted with MeOH to give Fr. IIIa2a1–Fr. IIIa2a2. and Fr. IIIa2a1 was purified using HPLC (MeOH/H_2_O, 8 : 2, 3 mL min^−1^), to give 2 (10 mg, *t*_R_ 10 min) and 3 (4 mg, *t*_R_ 12 min). Fr. IIIa2a2 was subjected to silica gel CC (PE/CH_2_Cl_2_, 3 : 1 → 1 : 1) to give 9 (13 mg).

#### (±)-Lindepentone A (1)

White amorphous powder; [*α*]^25^_D_ 0 (*c* 0.20, MeCN); UV (MeCN) *λ*_max_ (log *ε*) 261 (4.20), 194 (4.31) nm; IR (KBr) *ν*_max_ 1737, 1636, 1497, 1449, 1377, 1263, 1123 cm^−1^; for the ^1^H and ^13^C NMR data, see Table 1; ESIMS *m*/*z* 317.1 [M + H]^+^; HRESIMS *m*/*z* 317.1011 [M + H]^+^ (calcd for C_17_H_17_O_6_^+^, 317.1020).

#### Lindoxepine A (2)

White amorphous powder; UV (MeCN) *λ*_max_ (log *ε*) 322 (4.28), 264 (4.12), 235 (4.07), 228 (4.06) nm; IR (KBr) *ν*_max_ 1747, 1654, 1600, 1582, 1449, 1407, 1371, 1258, 1181, 1110 cm^−1^; for the ^1^H and ^13^C NMR data, see Table 1; ESIMS *m*/*z* 257.1 [M + H]^+^; HRESIMS *m*/*z* 257.0800 [M + H]^+^ (calcd for C_15_H_13_O_4_^+^, 257.0808).

#### Lindoxepine B (3)

White amorphous powder; UV (MeCN) *λ*_max_ (log *ε*) 320 (4.30), 257 (4.06), 235 (4.10), 228 (4.12) nm; IR (KBr) *ν*_max_ 1735, 1633, 1580, 1450, 1435, 1409, 1363, 1229, 1198, 1175, 1149 cm^−1^; for the ^1^H and ^13^C NMR data, see Table 1; ESIMS *m*/*z* 287.1 [M + H]^+^; HRESIMS *m*/*z* 287.0911 [M + H]^+^ (calcd for C_16_H_15_O_5_^+^, 287.0914).

#### (+)-Demethoxy-*epi*-bi-linderone (4a)

White amorphous powder; [*α*]^25^_D_ +274.4 (*c* 0.13, MeCN); UV (MeCN) *λ*_max_ (log *ε*) 296 (4.14), 248 (4.14) nm; ECD (*c* 0.87 × 10^−4^ M, MeCN) *λ*_max_ (Δ*ε*) 360 (+3.06), 290 (+12.38), 235 (+4.19) nm; IR (KBr) *ν*_max_ 2955, 2923, 2854, 1721, 1670, 1639, 1460, 1322, 1279, 1208, 1117, 1019 cm^−1^; for the ^1^H and ^13^C NMR data, see Table 2; ESIMS *m*/*z* 587.1 [M + H]^+^; HRESIMS *m*/*z* 587.1912 [M + H]^+^ (calcd for C_33_H_31_O_10_^+^, 587.1912).

#### (−)-Demethoxy-*epi*-bi-linderone (4b)

White amorphous powder; [*α*]^25^_D_ −286.8 (*c* 0.11, MeCN); ECD (*c* 0.87 × 10^−4^ M, MeCN) *λ*_max_ (Δ*ε*) 354 (−3.36), 291 (−12.77), 234 (−4.43) nm; the UV, IR, NMR, MS, and HRESIMS are the same as those of 4a.

### Computational section

The details of the ECD calculations for compounds 4a and 4b are presented in the ESI.†

### Antimicrobial assays

The test microorganisms obtained from the ATCC (American Type Culture Collection) in this bioassay were the bacteria *Bacillus subtilis* (ATCC 21332) (grows in beef-protein medium) and the fungi *Candida albicans* (ATCC 60193) (grows in YPD medium) and *Aspergillus niger* (GIM3.412) (grows in PDA medium). The minimum inhibitory concentrations (MICs) were determined using the microplate reader method according to the Clinical and Laboratory Standards Institute (CLSI) guidelines. For the antimicrobial assays, all of the compounds were dissolved in DMSO at a concentration of 4 mg mL^−1^, and were diluted with the corresponding medium to 100 μg mL^−1^ working solutions. The cultures were respectively inoculated with 1 × 10^6^ CFU (colony-forming units) mL^−1^ for B. *subtilis*, 1 × 10^3^ CFU mL^−1^ for *C. albicans*, and 0.4–5 × 10^4^ CFU mL^−1^ for *A. niger* in a final volume of 200 μL in 96-well tissue culture-treated microtiter plates (Greiner Bio-One, CELLSTAR) and incubated at 37 °C. The microbial growth was determined turbidimetrically (OD595) using a BIO-RAD iMark microplate reader. The MIC values were obtained after 16 h for the bacteria or 24 h for the fungi. The resulting values were compared with the values for a positive control under the same conditions.

## Conclusions

Eleven *Lindera* cyclopentenedione derivatives including a novel 3,5-dioxocyclopent-1-enecarboxylate derivative (1), two novel oxepine-2,5-dione derivatives (2 and 3), a pair of new enantiomers of bi-linderone derivatives (4a/4b) as well as six known ones (5–8 and 9a/9b) were obtained from the dry roots of *L. aggregata*. Their structures, including the absolute configurations, were determined by comprehensive spectroscopic analysis and ECD calculations. Interestingly, lindoxepines A (2) and B (3) present an unprecedented oxepine-2,5-dione derivative skeleton, and may be the key intermediates for the synthesis of *Lindera* cyclopentenediones *in vivo* from the proposed precursor stilbenes. A putative pathway to the biosynthesis of a novel skeleton of 3,5-dioxocyclopent-1-enecarboxylate, (±)-lindepentone A (1), was also proposed from a stilbene precursor. To the best of our knowledge, the novel structures of 2 and 3 discovered from *L. aggregata* may be enlightening for the *in vivo* biosynthesis of the monomers of *Lindera* cyclopentenediones. In addition, the synthesized *epi*-bi-linderone (5) was also isolated and identified as a natural product, which provides evidence for the existence of the high biomimetic biosynthetic pathways for *Lindera* cyclopentenedione dimers proposed by Wang *et al.*^8^ Antimicrobial assays showed that compounds 6 and 7 exhibited weak inhibitory activity against *Candida albicans* with a MIC value of 25 μg mL^−1^, while other compounds were inactive (MIC values: >50 μg mL^−1^) against the tested microorganisms.

## Conflicts of interest

There are no conflicts to declare.

## Supplementary Material

RA-008-C8RA03094D-s001
