# Supplementary material for: Lindera cyclopentenedione intermediates from the roots of Lindera aggregata
Source: RSC Adv. 2018 May 16;8(32):17898–904. doi: 10.1039/c8ra03094d (PMC9080501; doi:10.1039/c8ra03094d)
Supplement: RA-008-C8RA03094D-s001 [file RA-008-C8RA03094D-s001.pdf]

Electronic Supplementary Material (ESI) for RSC Advances.
This journal is © The Royal Society of Chemistry 2018

Electronic Supplementary Information

Lindera cyclopentenedione intermediates from the roots of *Lindera aggregate*

Lin Chen[a], Bo Liu[b], Jun-Jie Deng[a], Jun-Sheng Zhang[a], Wei Li[a], Abrar Ahmed[a], Sheng Yin[a], Gui-Hua Tang[a,]*

[a] School of Pharmaceutical Sciences, Sun Yat-sen University, Guangzhou, Guangdong 510006, China

[b] The Second Clinical Medical College, Guangzhou University of Chinese Medicine, Guangzhou 510006, China

*Corresponding author.

E-mail addresses: tanggh5@mail.sysu.edu.cn

Contents:

S1. Tables······p4–6

**Table S1.1.** The ^{1}H NMR (400 MHz) and ^{13}C NMR (100 MHz) data of **5** in $CDCl_3$ (δ in ppm).
**Table S1.2.** The ^{1}H NMR (400 MHz) and ^{13}C NMR (100 MHz) data of **6** in $CDCl_3$ (δ in ppm).
**Table S1.3.** The ^{1}H NMR (400 MHz) and ^{13}C NMR (100 MHz) data of **7** in $CDCl_3$ (δ in ppm).
**Table S1.4.** The ^{1}H (400 MHz) and ^{13}C (100 MHz) NMR data of **8** and **9a/9b** in $CDCl_3$ (δ in ppm).

S2. NMR spectra of compounds **1–7**······p7–38

in CD_3OD	
**S2.1.** ^{1}H NMR spectrum of compound **1**	**S2.2.** ^{13}C NMR and DEPT spectra of compound **1**
**S2.3.** HSQC spectrum of compound **1**	**S2.4.** ^{1}H–^{1}H COSY spectrum of compound **1**
**S2.5.** HMBC spectrum of compound **1**	**S2.6.** NOESY spectrum of compound **1**
in $CDCl_3$	
**S2.7.** ^{1}H NMR spectrum of compound **2**	**S2.8.** ^{13}C NMR and DEPT spectra of compound **2**
**S2.9.** HSQC spectrum of compound **2**	**S2.10.** ^{1}H–^{1}H COSY spectrum of compound **2**
**S2.11.** HMBC spectrum of compound **2**	**S2.12.** ^{1}H NMR spectrum of compound **3**
**S2.13.** ^{13}C NMR and DEPT spectra of compound **3**	**S2.14.** HSQC spectrum of compound **3**
**S2.15.** ^{1}H–^{1}H COSY spectrum of compound **3**	**S2.16.** HMBC spectrum of compound **3**
**S2.17.** ^{1}H NMR spectrum of compounds **4a** and **4b**	**S2.18.** ^{13}C NMR and DEPT spectra of compounds **4a** and **4b**
**S2.19.** HSQC spectrum of compounds **4a** and **4b**	**S2.20.** ^{1}H–^{1}H COSY spectrum of compounds **4a** and **4b**
**S2.21.** HMBC spectrum of compounds **4a** and **4b**	**S2.22.** NOESY spectrum of compounds **4a** and **4b**
**S2.23.** ^{1}H NMR spectrum of compound **5**	**S2.24.** ^{13}C NMR and DEPT spectra of compound **5**
**S2.25.** ^{1}H NMR spectrum of compound **6**	**S2.26.** ^{13}C NMR and DEPT spectra of compound **6**

**S2.27.** ^{1}H NMR spectrum of compound **7**	**S2.28.** ^{13}C NMR and DEPT spectra of compound **7**
**S2.29.** ^{1}H NMR spectrum of compound **8**	**S2.30.** ^{13}C NMR and DEPT spectra of compound **8**
**S2.31.** ^{1}H NMR spectrum of compounds **9a** and **9b**	**S2.32.** ^{13}C NMR and DEPT spectra of compounds **9a** and **9b**

S3. MS, HR-ESI-MS, and IR spectra of new compounds **1–4**··················p39–46

**S3.1.** MS data of compound **1**	**S3.2.** HR-ESI-MS data of compound **1**
**S3.3.** MS data of compound **2**	**S3.4.** HR-ESI-MS data of compound **2**
**S3.5.** MS data of compound **3**	**S3.6.** HR-ESI-MS data of compound **3**
**S3.7.** MS data of compounds **4a** and **4b**	**S3.8.** HR-ESI-MS data of compounds **4a** and **4b**
**S3.9.** IR (KBr disc) spectrum of compound **1**	**S3.10.** IR (KBr disc) spectrum of compound **2**
**S3.11.** IR (KBr disc) spectrum of compound **3**	**S3.12.** IR (KBr disc) spectrum of compounds **4a** and **4b**

S4. ECD computational details···p47–49

Table S1.1. The ^{1}H (400 MHz) and ^{13}C (100 MHz) NMR data of **5** in $CDCl_3$ (δ in ppm)

position	δ_H, multi (J in Hz)	δ_C, type	position	δ_H, multi (J in Hz)	δ_C, type
1		155.2, C	1′		173.2, C
2	4.83 d (5.1)	95.6, CH	2′	5.89, d (11.6)	43.9, CH
3	3.66, d (5.1)	47.3, CH	3′	3.82, d (11.6)	43.1, CH
4		56.9, C	4′		111.1, C
5, 8		195.7 and 193.5, each C	5′, 8′		186.5 and 183.9, each C
6, 7		151.6 and 150.7, each C	6′, 7′		147.9 and 146.8, each C
9		141.5, C	9′		136.5, C
10/14, 11/13, 12	6.99–7.36, m, overlapped	129.7 × 2, 128.2 × 2, and 127.6, each CH	10′/14′, 11′/13′, 12′	6.99–7.36, m, overlapped	129.7 × 2, 127.8 × 2, and 127.4, each CH
1-OMe	3.58, s	55.0, CH_3	1′-OMe	4.10, s	65.9, CH_3
6-, 7-OMe	4.14 and 3.73, each s	59.66 and 59.74, each CH_3	6′-, 7′-OMe	4.08 and 4.04, each s	60.0 and 59.66, each CH_3

Table S1.2. The ^{1}H (400 MHz) and ^{13}C (100 MHz) NMR data of **6** in $CDCl_3$ (δ in ppm)

position	δ_H, multi (J in Hz)	δ_C, type	position	δ_H, multi (J in Hz)	δ_C, type
1		154.9, C	1′		172.8, C
2	4.84, br s	96.6, CH	2′	6.14, d (11.3)	43.4, CH
3	4.32, br s	46.1, CH	3′	3.88, d (11.3)	48.5, CH
4		59.1, C	4′		111.2, C
5, 8		196.2 and 194.8, each C	5′, 8′		186.8 and 184.1, each C
6, 7		153.7 and 153.0, each C	6′, 7′		147.9 and 147.0, each C
9		140.3, C	9′		136.4, C
10/14, 11/13, 12	7.04–7.22, m, overlapped	129.2 × 2, 128.4 × 2, and 127.7, each CH	10′/14′, 11′/13′, 12′	7.04–7.22, m, overlapped	129.2 × 2, 128.2 × 2, and 127.6, each CH
1-OMe	3.59, s	55.0, CH_3	1′-OMe	4.00, s	65.2, CH_3
6-, 7-OMe	3.76 and 3.61, each s	59.4 and 59.6, each CH_3	6′-, 7′-OMe	4.12 and 4.05, each s	59.7 and 60.1, each CH_3

Table S1.3. The ^{1}H (400 MHz) and ^{13}C (100 MHz) NMR data of **7** in $CDCl_3$ (δ in ppm)

position	δ_H, multi (J in Hz)	δ_C, type
1/1′		153.4, C
2/2′	5.43, d (10.3)	102.4, CH
3/3′	5.55, d (10.3)	43.4, CH
4/4′		66.8, C
5/5′, 8/8′		194.9 and 192.4, each C
6/6′, 7/7′		154.1 and 152.0, each C
9/9′		138.2, C
10(10′)/14(14′), 11(11′)/13(13′), 12(12′)	7.23–7.29, m, overlapped	129.8 × 2, 128.6 × 2, and 127.9, each CH
1/1′-OMe	3.50, s	55.6, CH_3
6/6′-, 7/7′-OMe	3.95 and 3.89, each s	59.68 and 59.65, each CH_3

Table S1.4. The ^{1}H (400 MHz) and ^{13}C (100 MHz) NMR data of **6a**, **6b**, and **7** in $CDCl_3$ (δ in ppm).

position	**8**		**9a**		**9b**	
	δ_H, multi (J in Hz)	δ_C, type	δ_H, multi (J in Hz)	δ_C, type	δ_H, multi (J in Hz)	δ_C, type
1		186.9, C		191.8, C		189.7, C
2		109.3, C		109.4, C		109.7, C
3		184.4, C		185.5, C		188.4, C
4		147.6, C		170.2, C		169.9, C
5		148.8, C	5.94, s	111.9, CH	5.93, s	112.2, CH
6		165.1, C		168.9, C		169.1, C
7	7.37, d (15.8)	121.1, CH	7.61, d (15.8)	121.6, CH	7.60, d (15.8)	121.2, CH
8	7.81, d (15.8)	140.9, CH	8.00, d (15.8)	142.8, CH	7.92, d (15.8)	143.0, CH
9		135.4, C		135.6, C		135.4, C
10/14	7.46, dd, (7.8, 1.8)	128.7, CH	7.38, m, overlapped	129.04, CH	7.38, m, overlapped	129.07, CH
11/13	7.23, m, overlapped	128.1, CH	7.38, m, overlapped	128.7, CH	7.38, m, overlapped	128.7, CH
12	7.23, m, overlapped	129.8, CH	7.38, m, overlapped	130.47, CH	7.38, m, overlapped	130.50, CH
4-OMe	3.05, s	59.7, CH_3	3.93, s	58.7, CH_3	3.92, s	58.6, CH_3
5-OMe	4.04, s	59.6, CH_3				
6-OMe	3.97, s	64.1, CH_3	4.19, s	64.7, CH_3	4.21, s	65.1, CH_3

S2.1. ^{1}H NMR spectrum of compound **1**

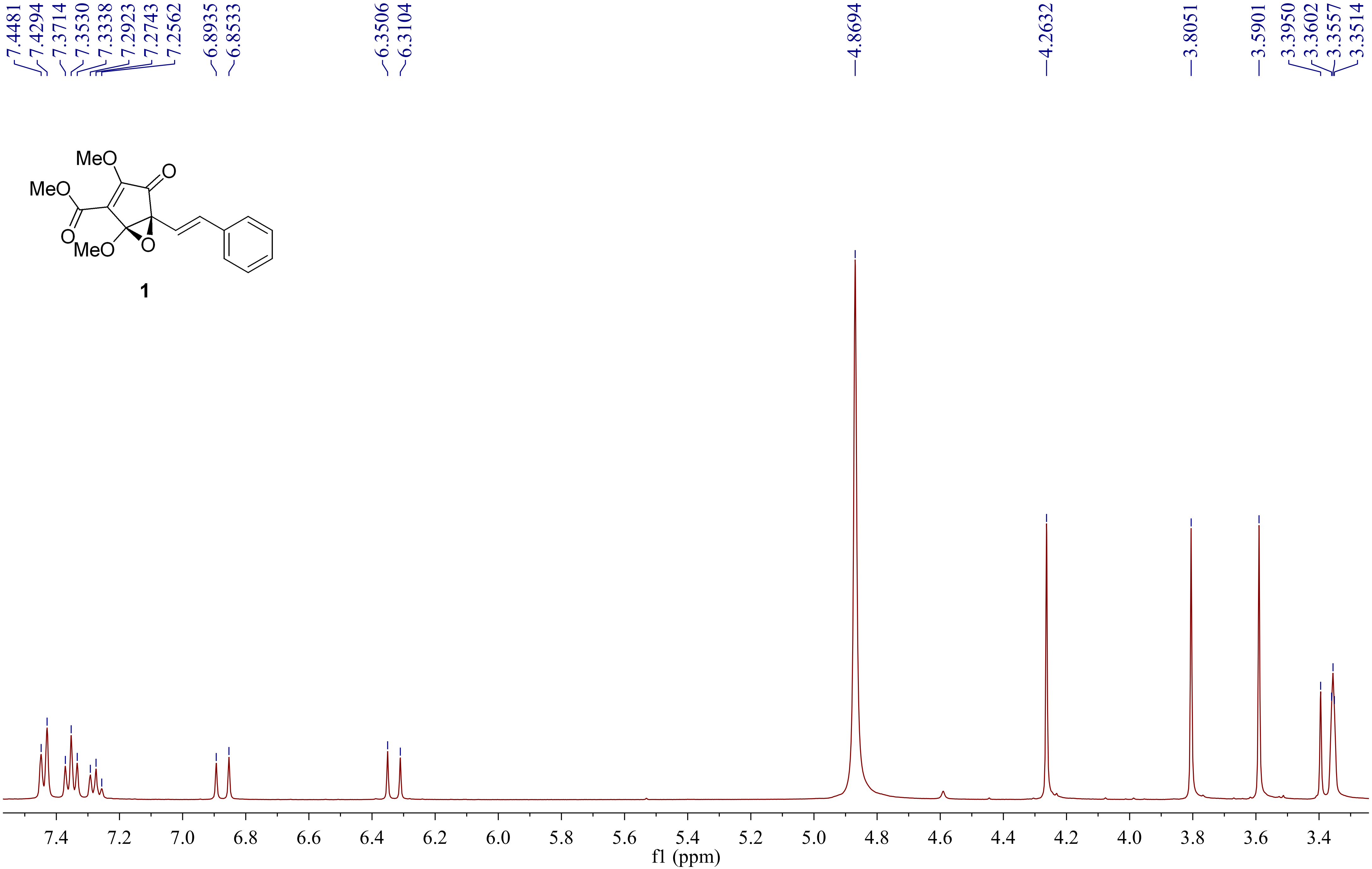

S2.2. ^{13}C NMR and DEPT spectra of compound **1**

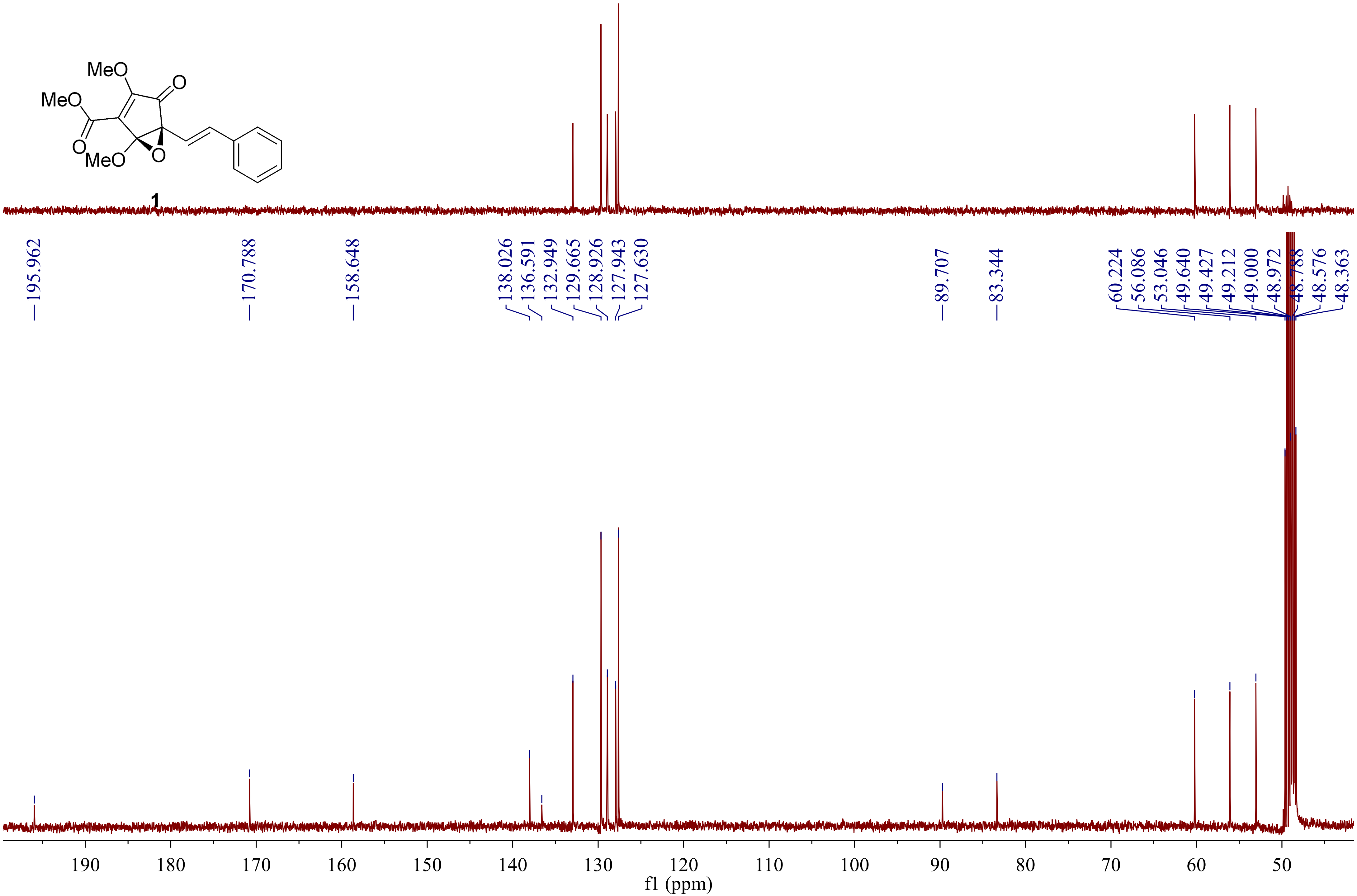

S2.3. HSQC spectrum of compound **1**

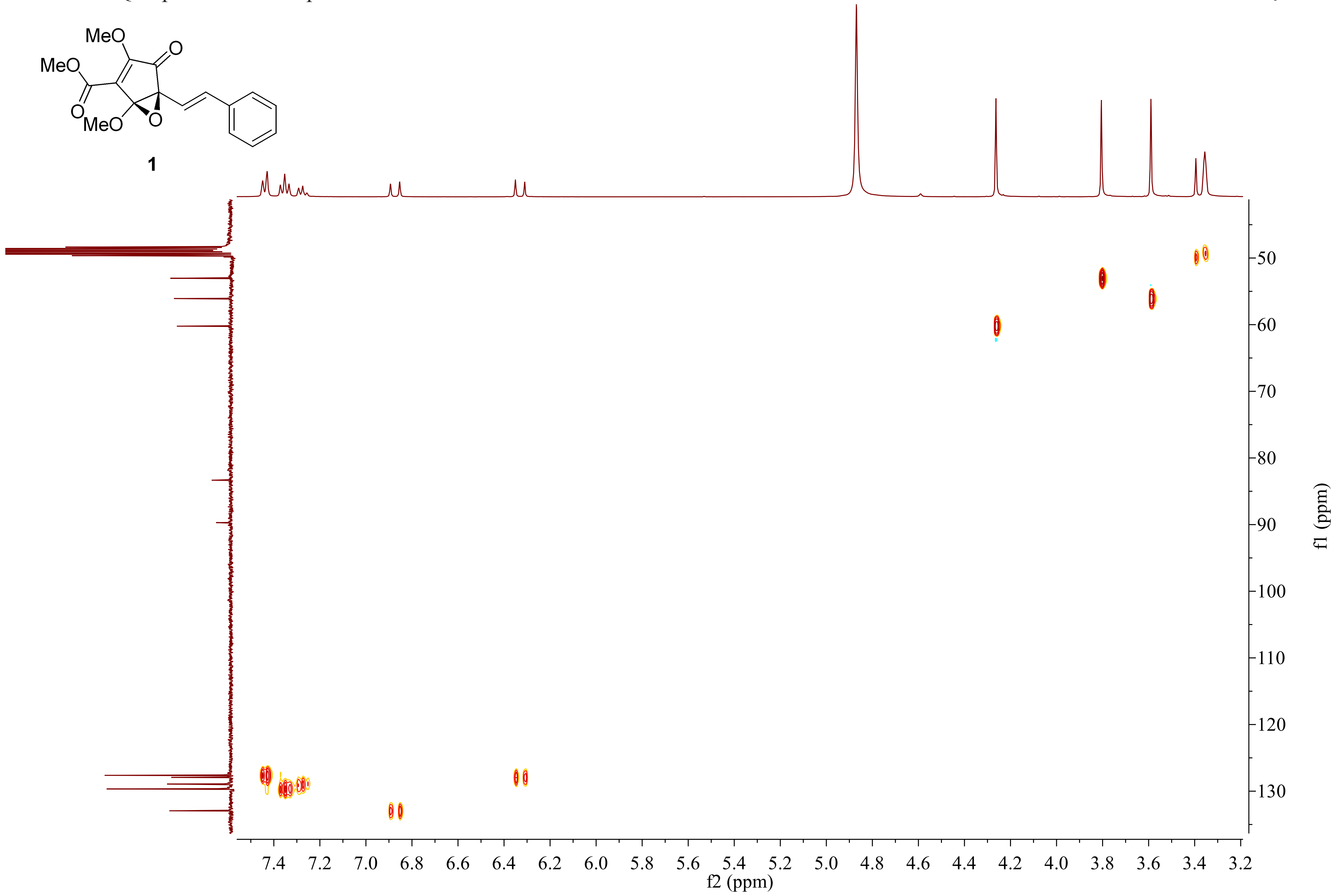

S2.4. ^{1}H-^{1}H COSY spectrum of compound **1**

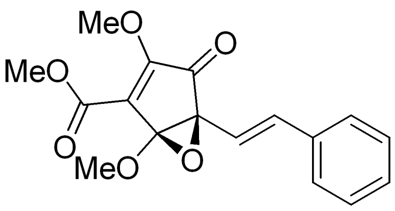

1

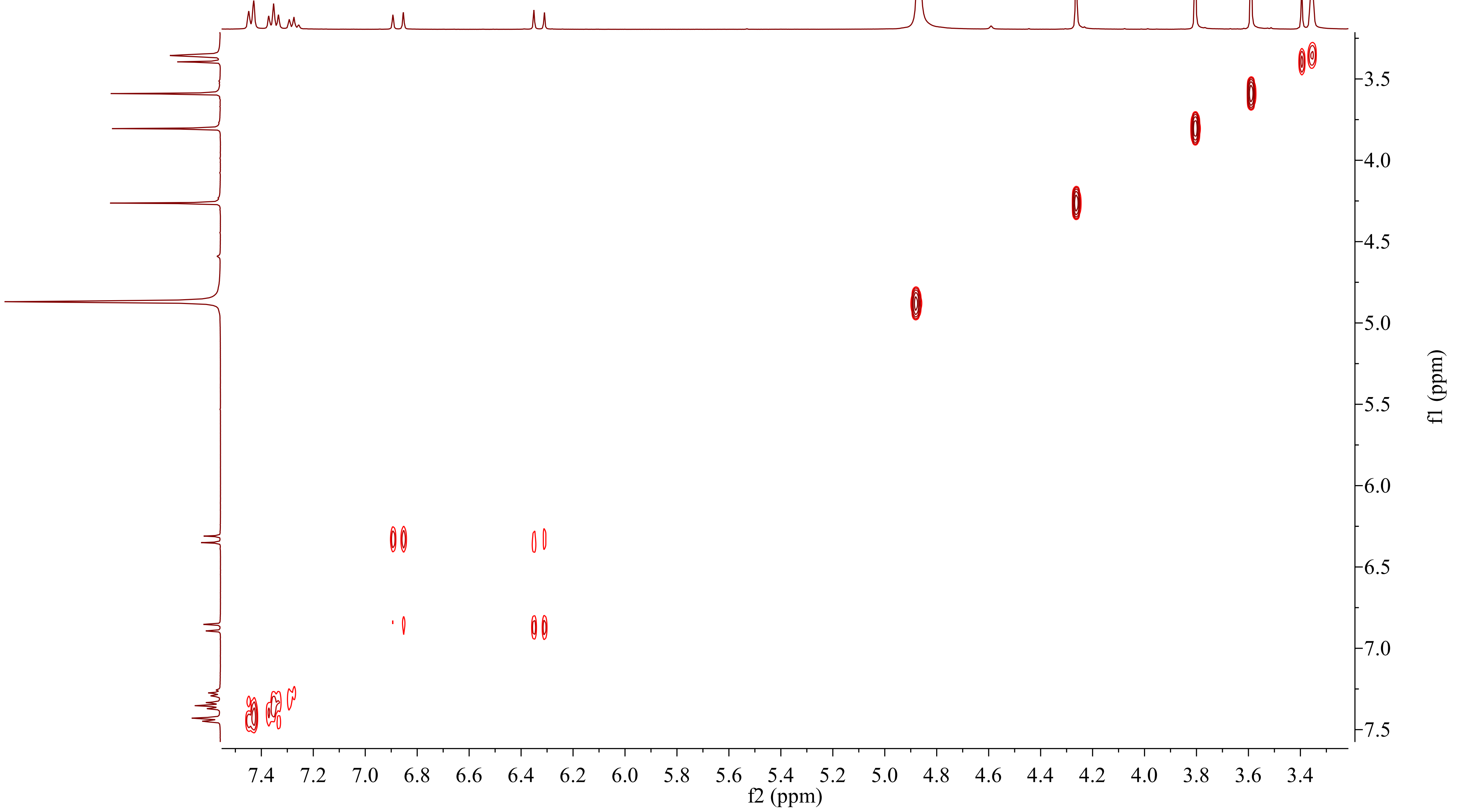

S2.5. HMBC spectrum of compound **1**

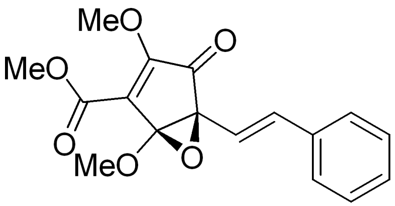

1

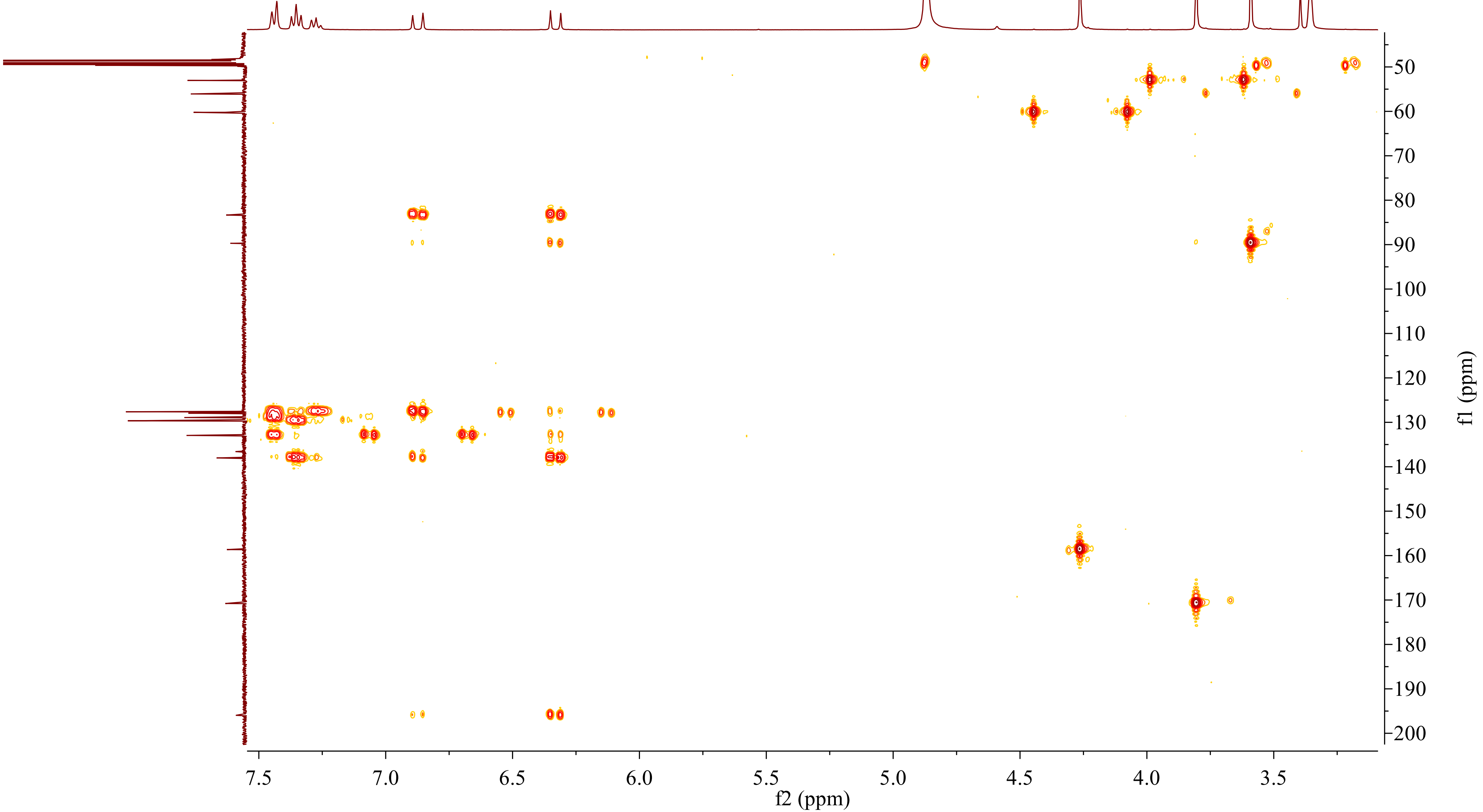

S2.6. NOESY spectrum of compound **1**

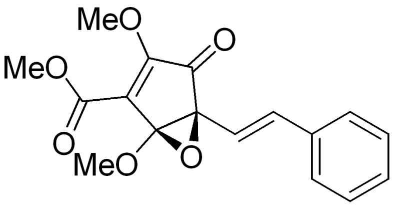

1

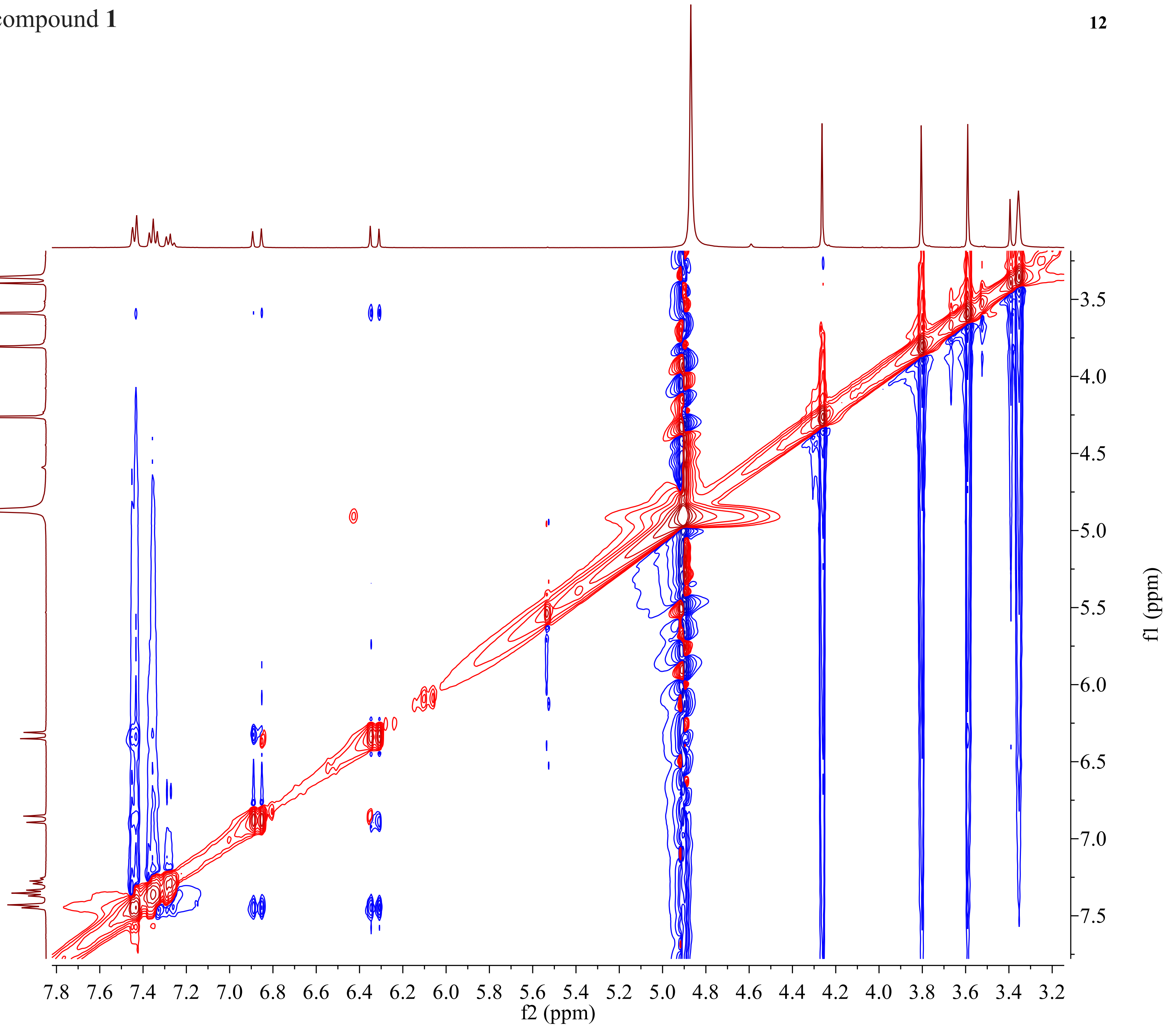

S2.7. ^{1}H NMR spectrum of compound **2**

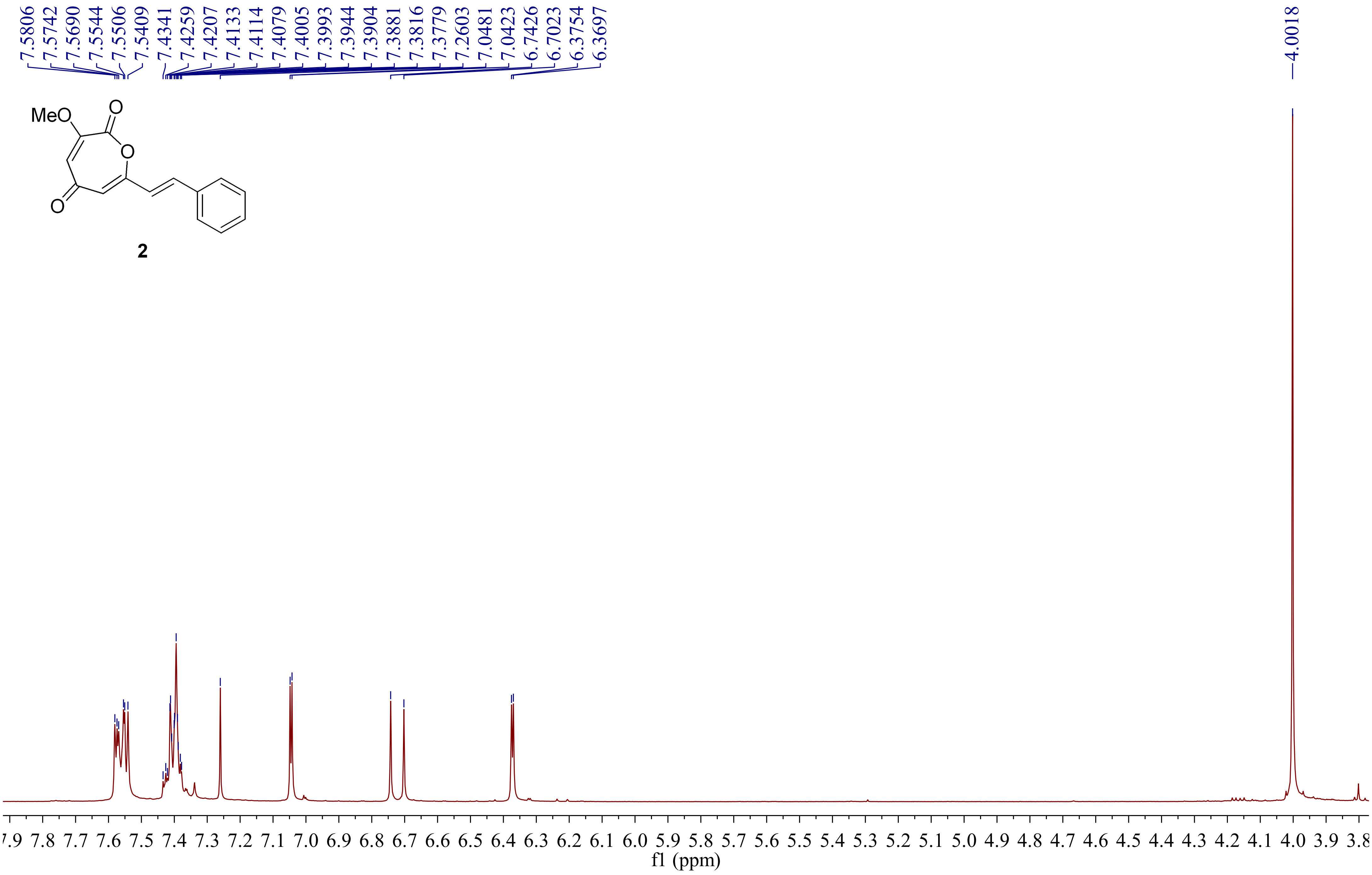

S2.8. ^{13}C NMR and DEPT spectra of compound **2**

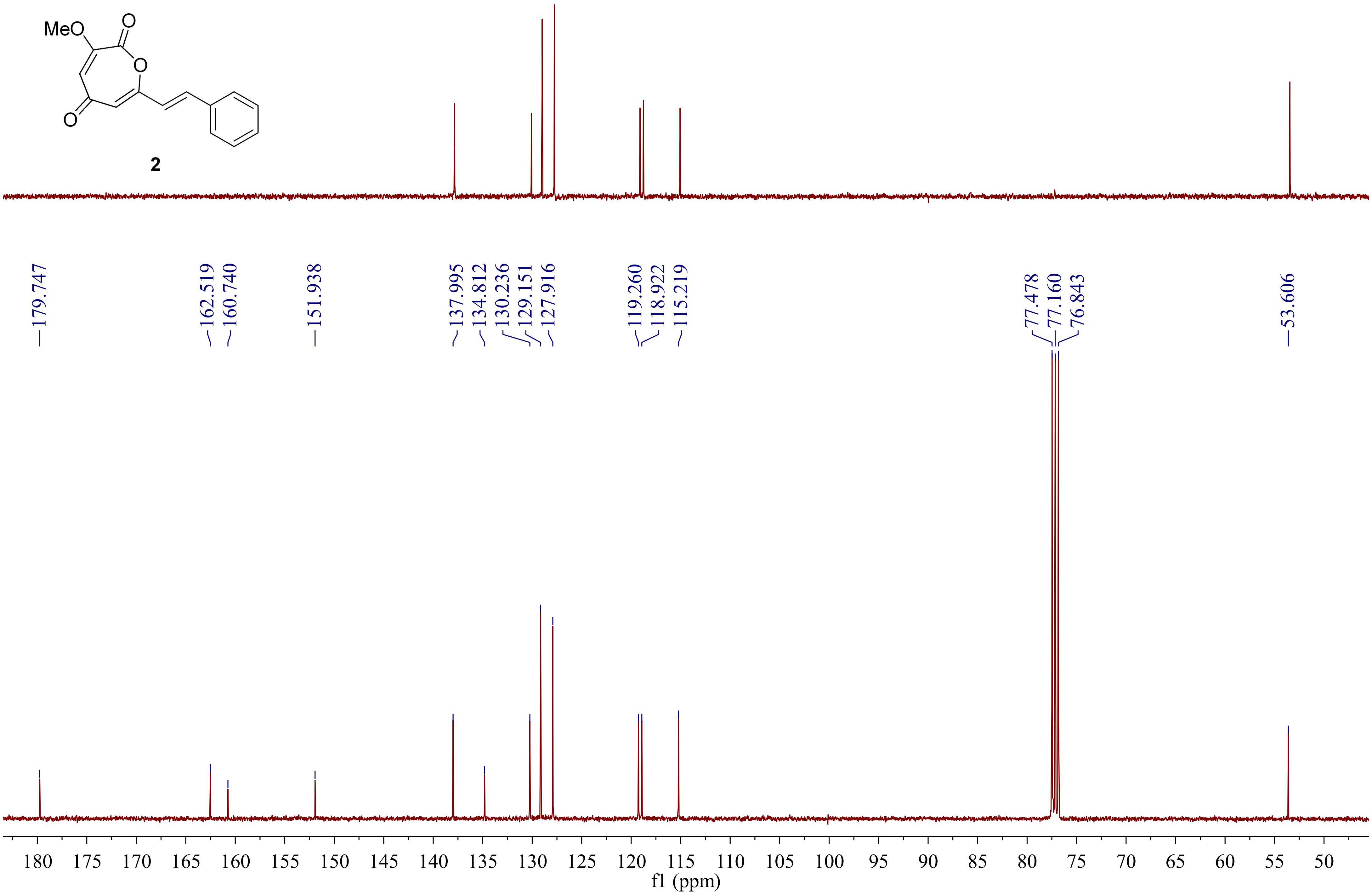

S2.9. HSQC spectrum of compound **2**

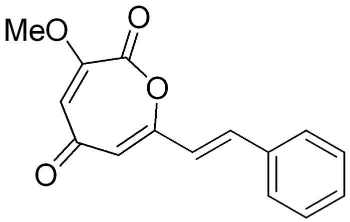

2

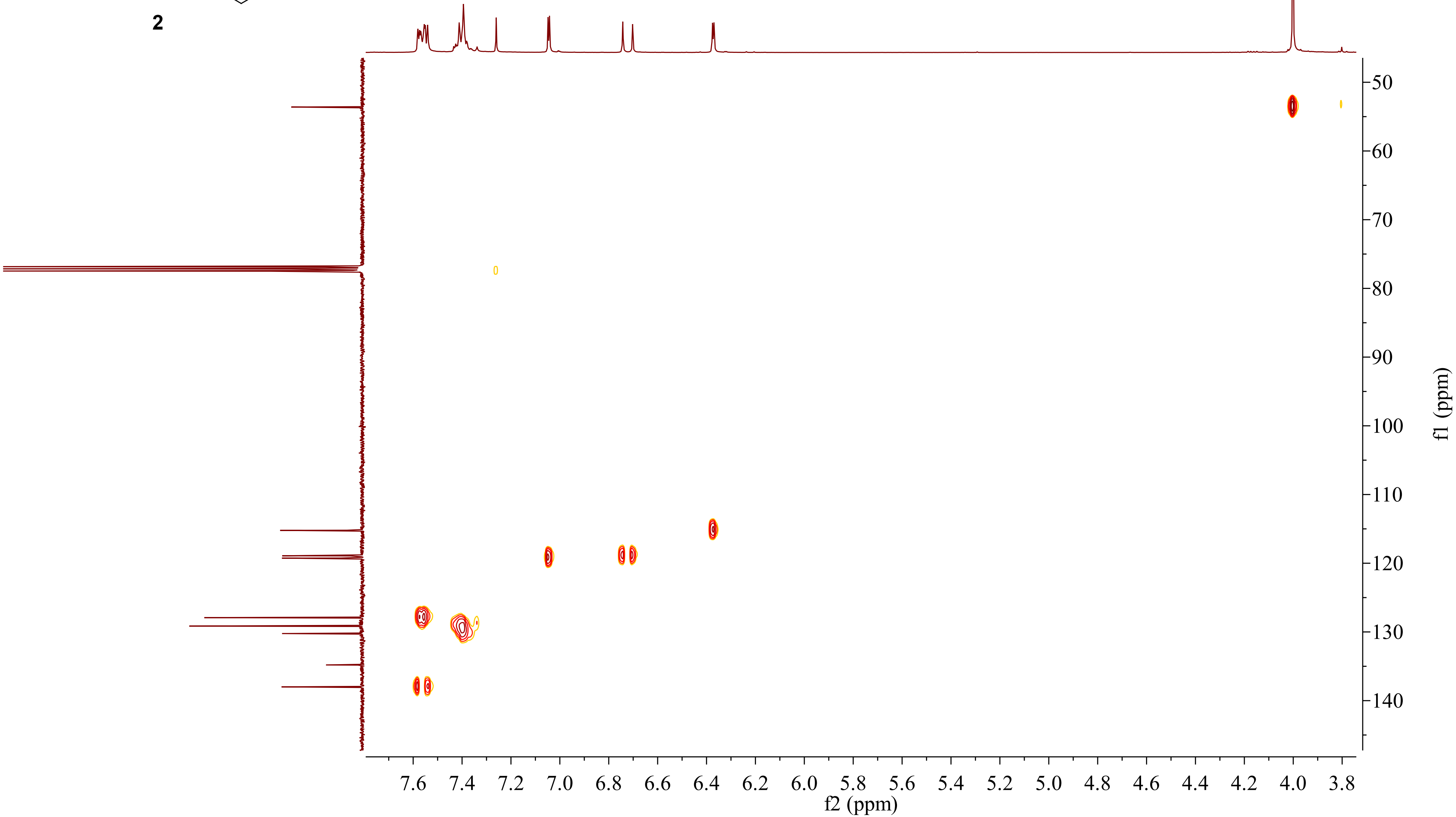

S2.10. ^{1}H-^{1}H COSY spectrum of compound **2**

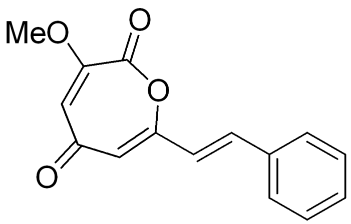

2

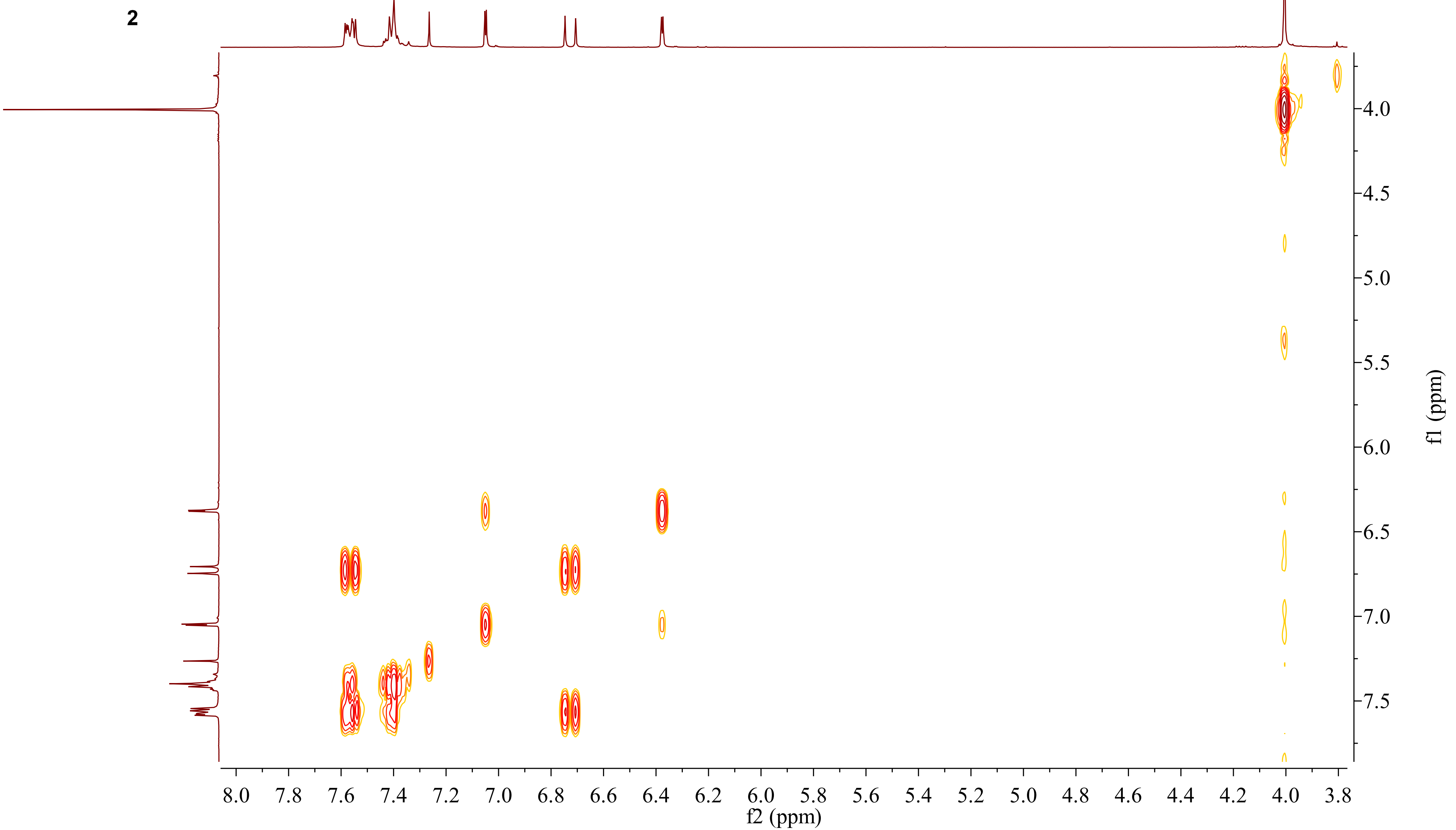

S2.11. HMBC spectrum of compound **2**

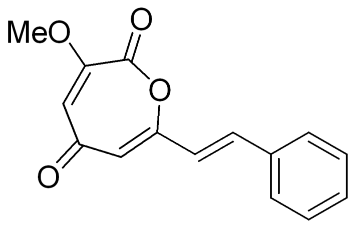

2

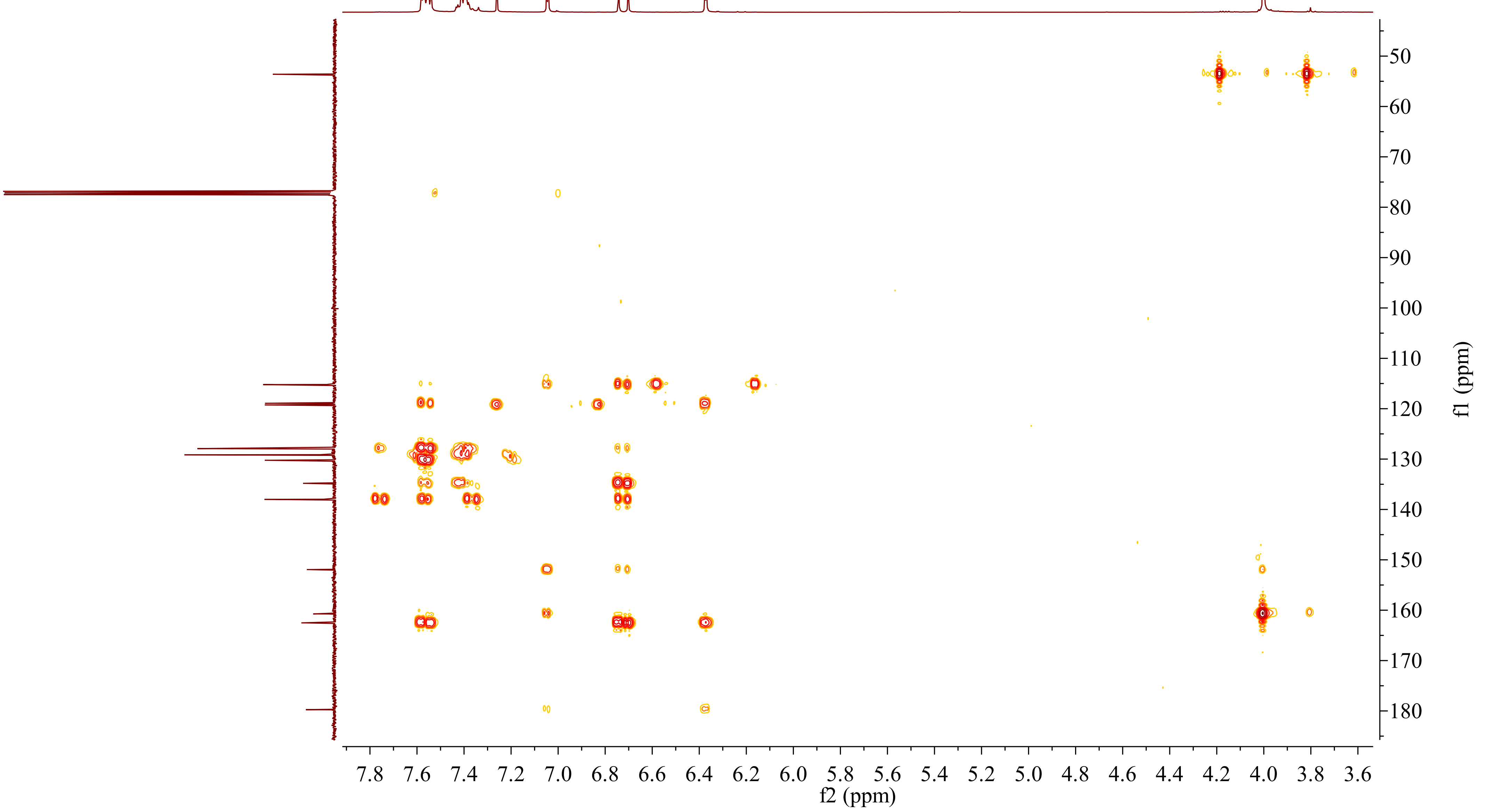

S2.12. ^{1}H NMR spectrum of compound **3**

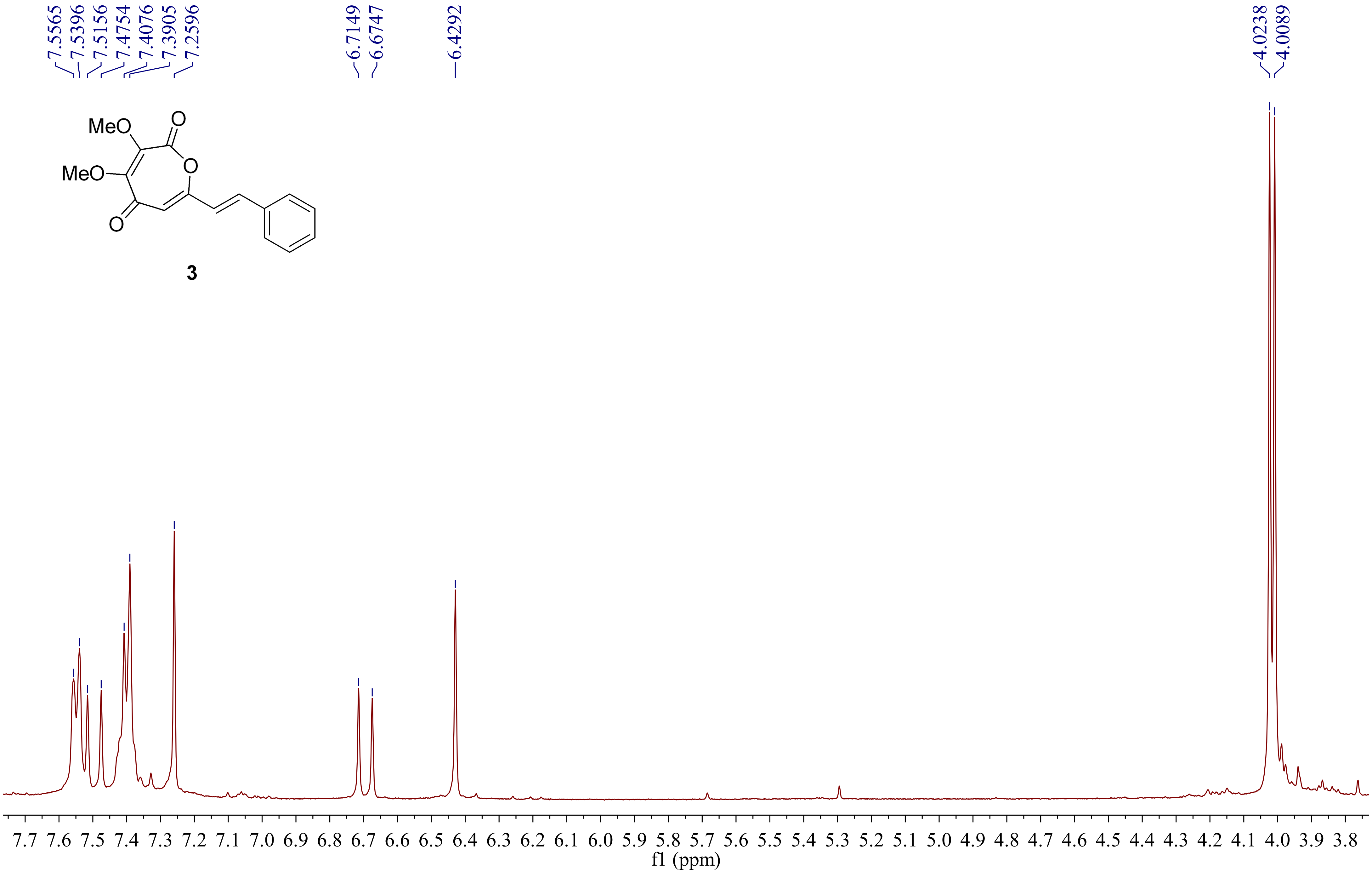

S2.13. ^{13}C NMR and DEPT spectra of compound **3**

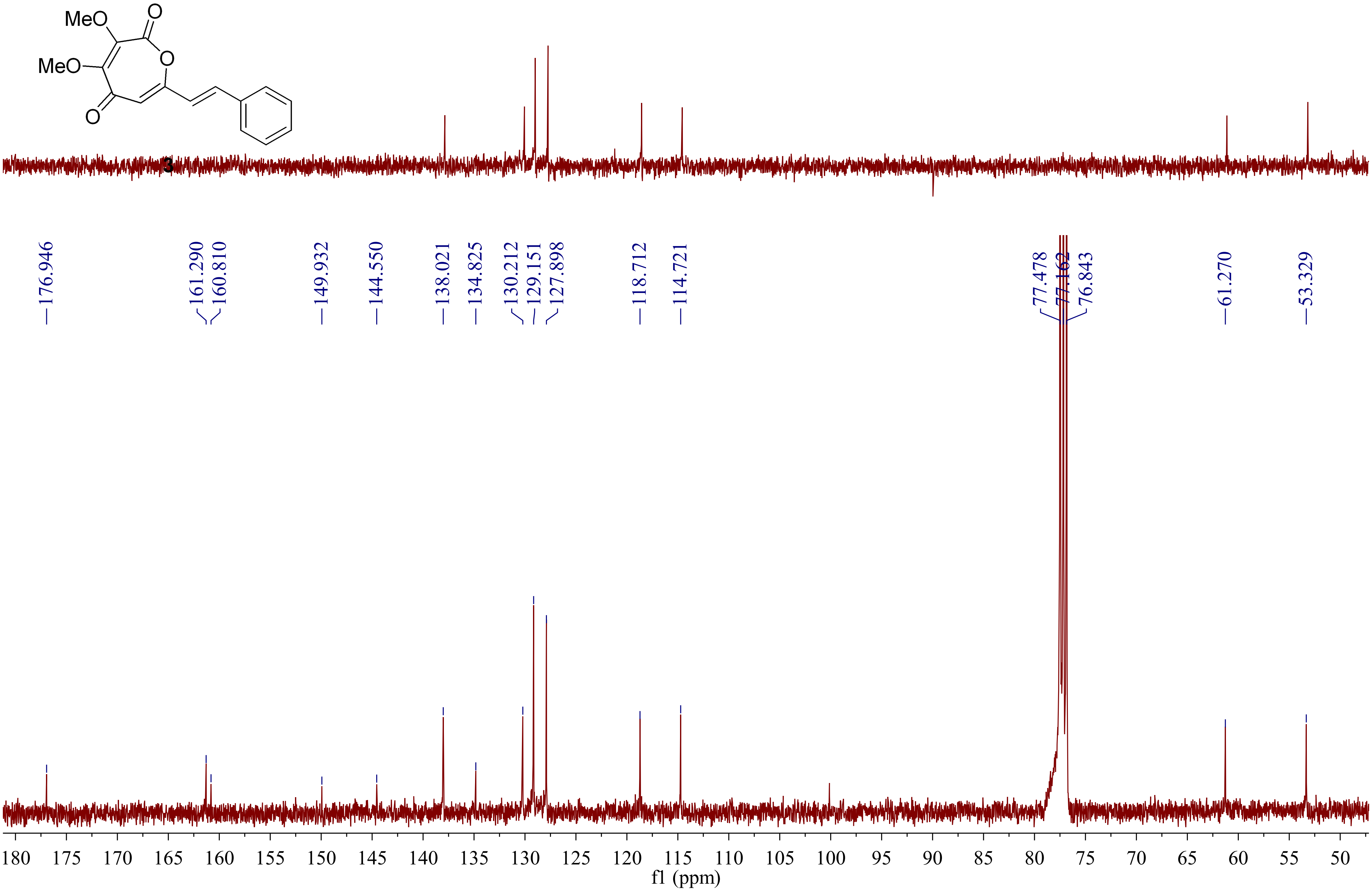

S2.14. HSQC spectrum of compound **3**

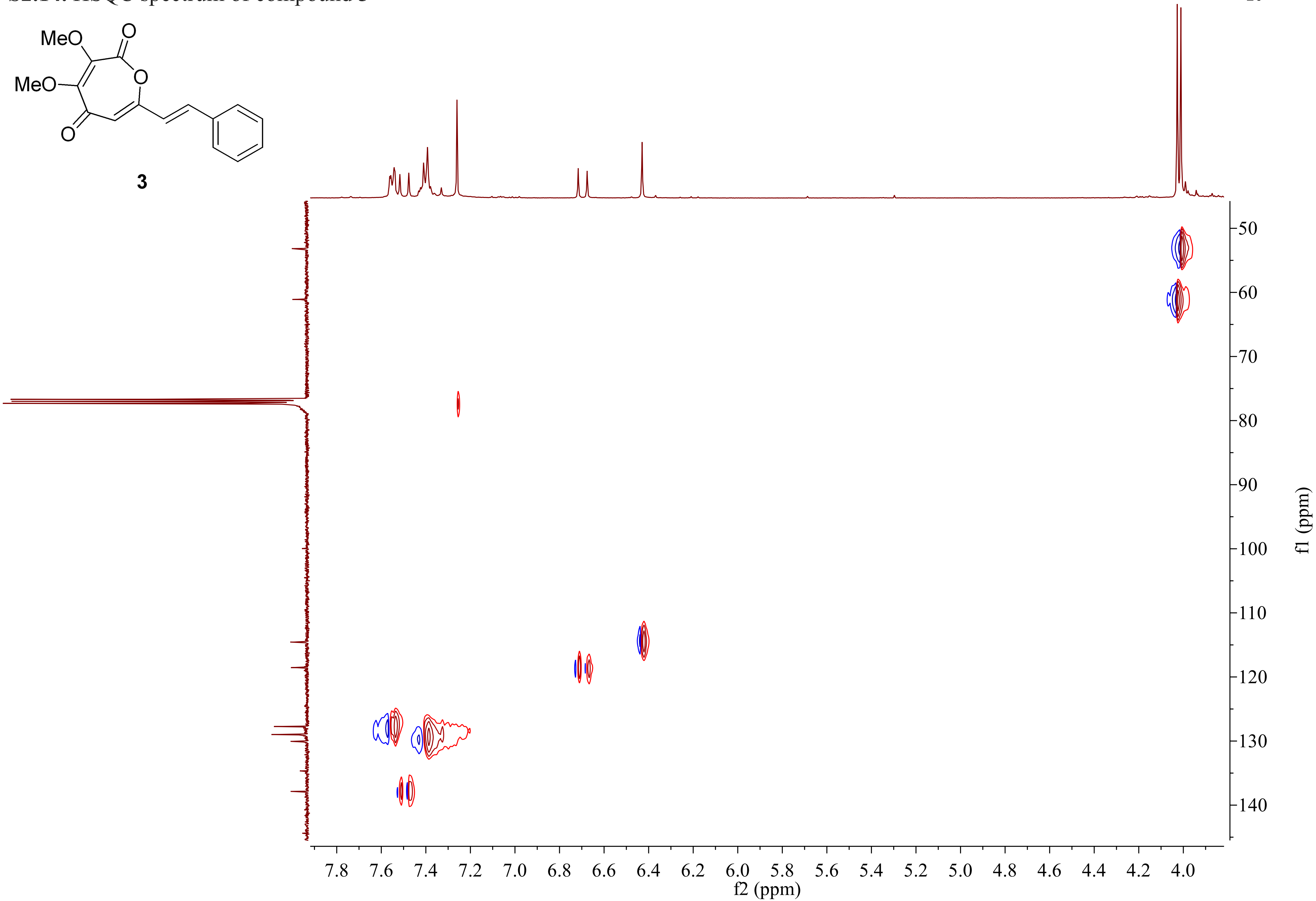

S2.15. ^{1}H-^{1}H COSY spectrum of compound **3**

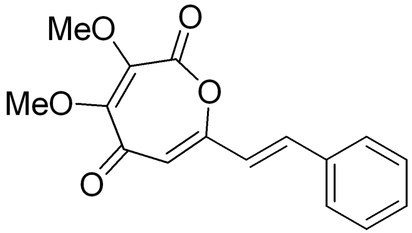

3

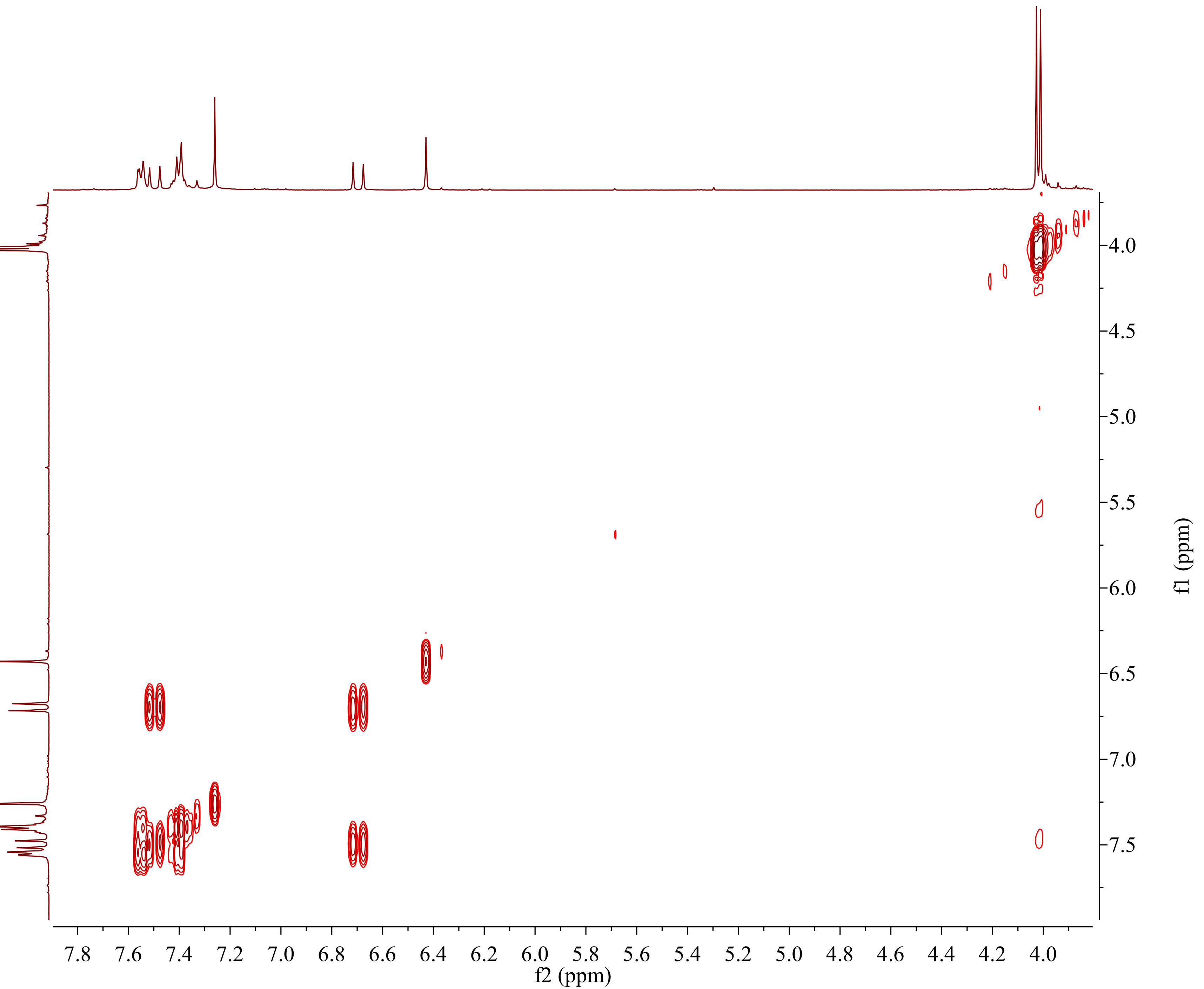

S2.16. HMBC spectrum of compound **3**

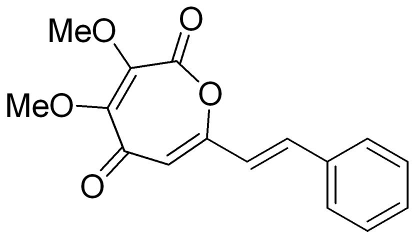

3

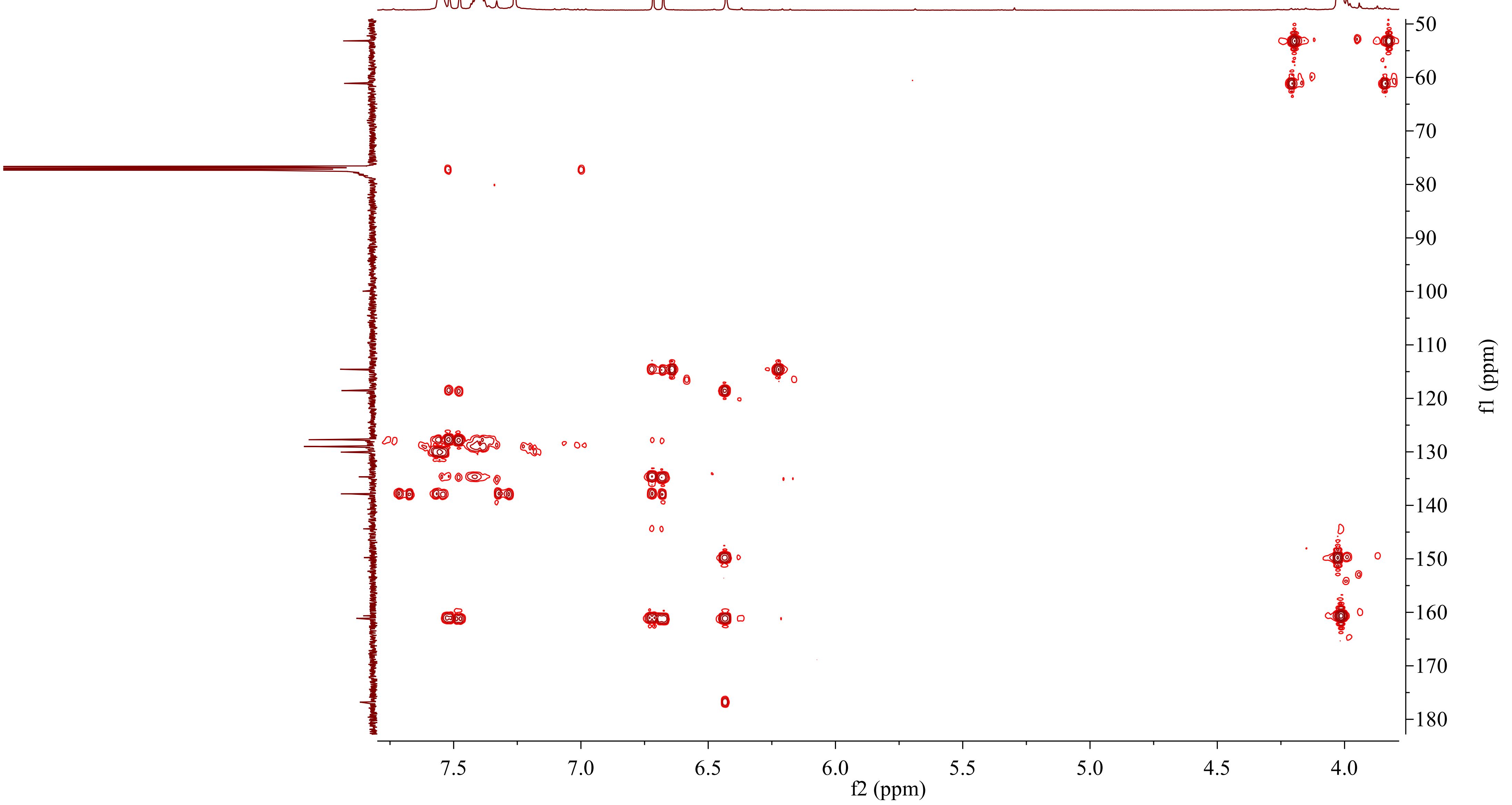

S2.17. ^{1}H NMR spectrum of compounds **4a** and **4b**

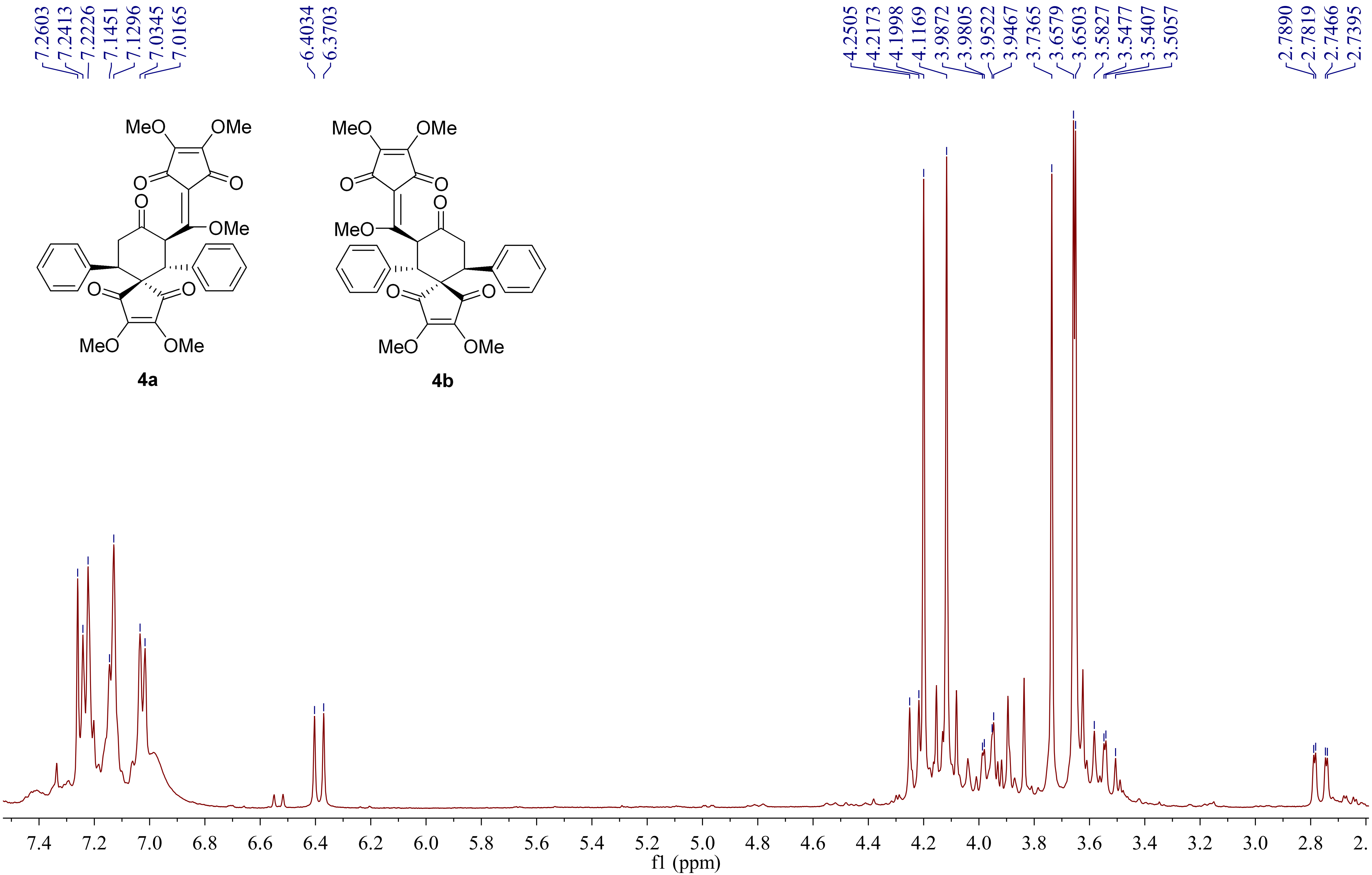

S2.18. ^{13}C NMR and DEPT spectra of compounds **4a** and **4b**

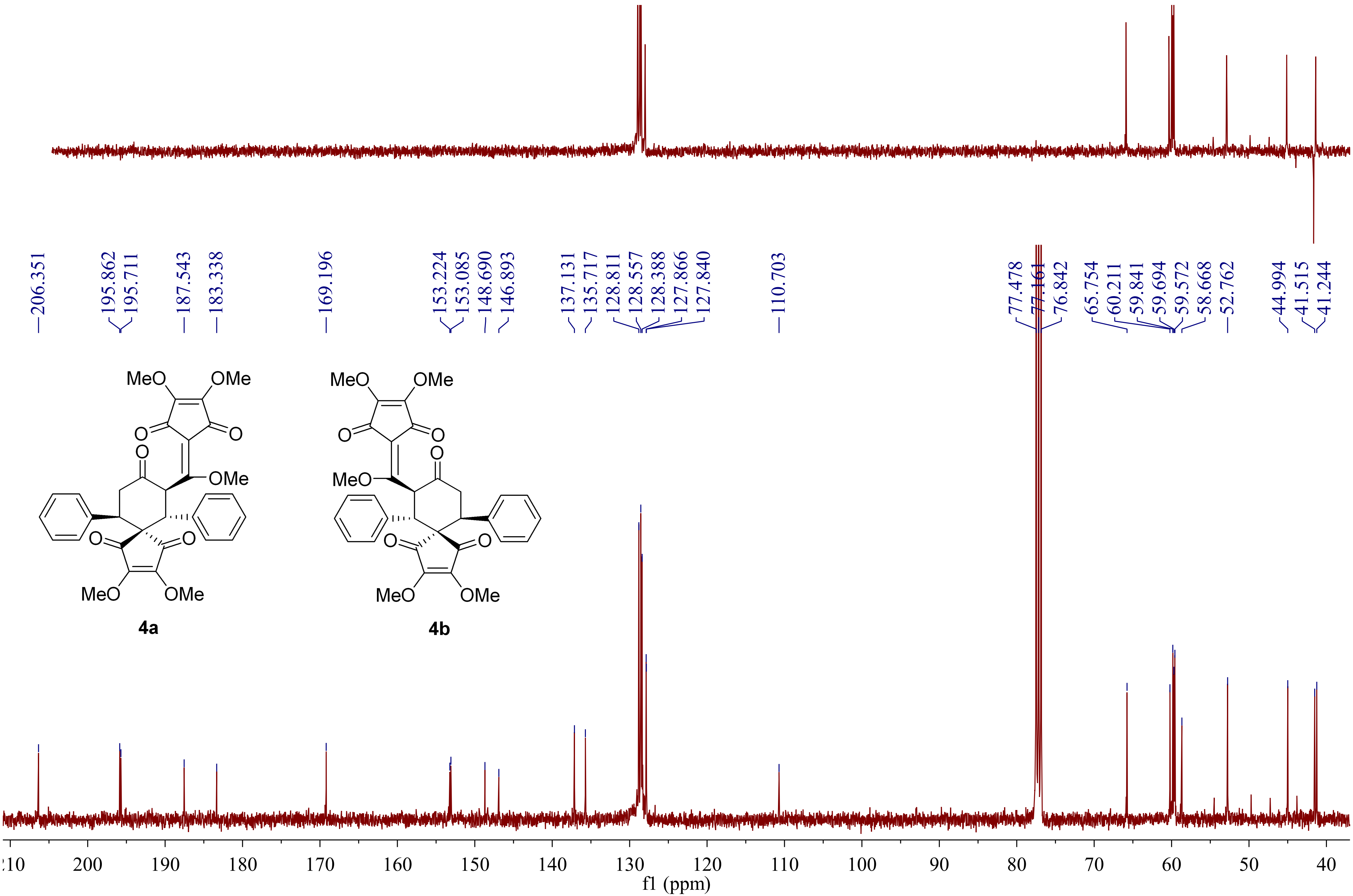

S2.19. HSQC spectrum of compounds **4a** and **4b**

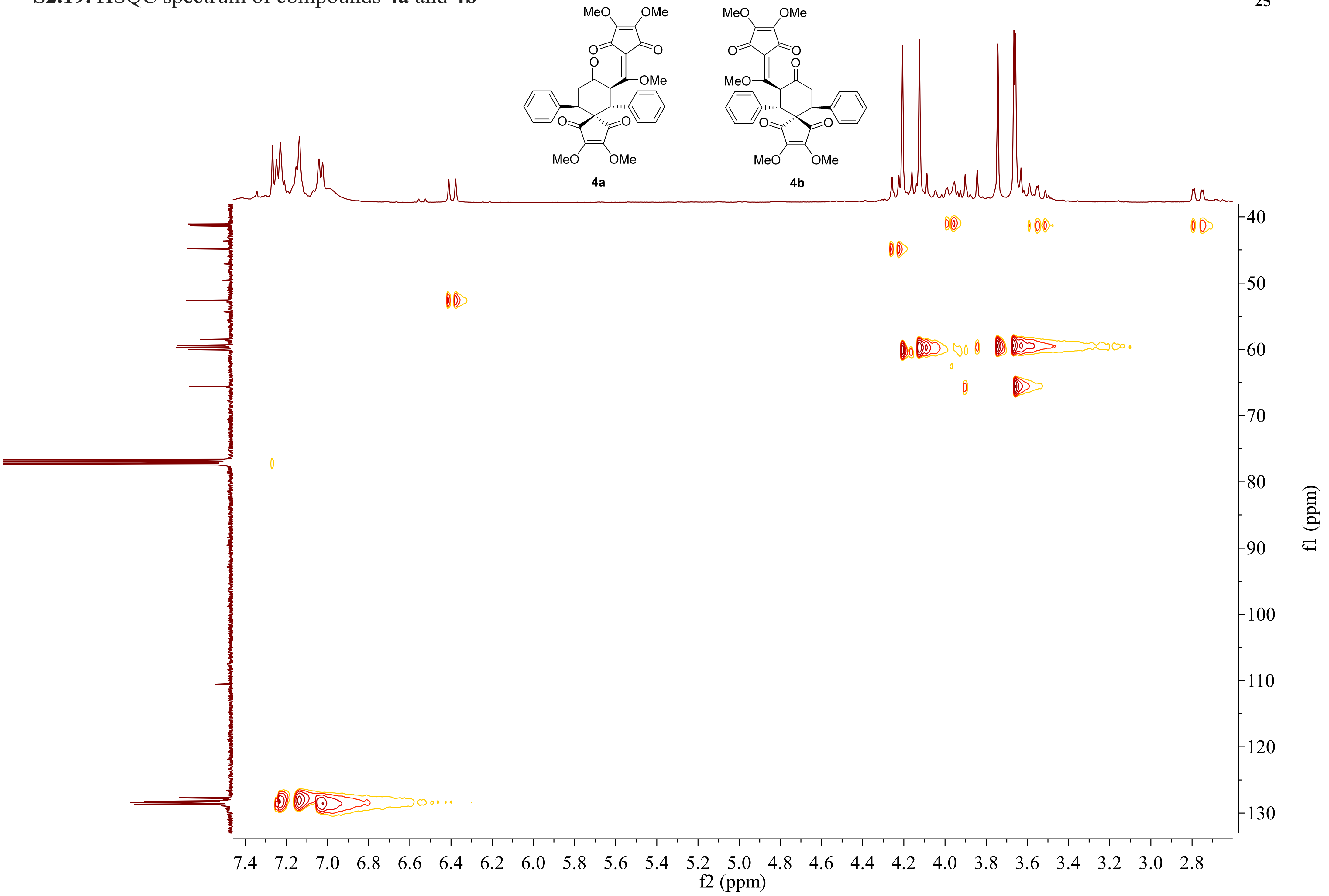

S2.20. ^{1}H-^{1}H COSY spectrum of compounds **4a** and **4b**

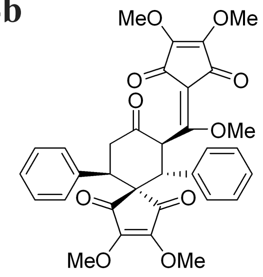

4a

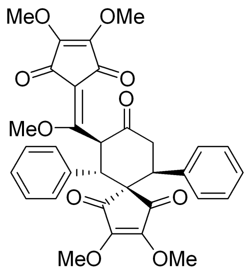

4b

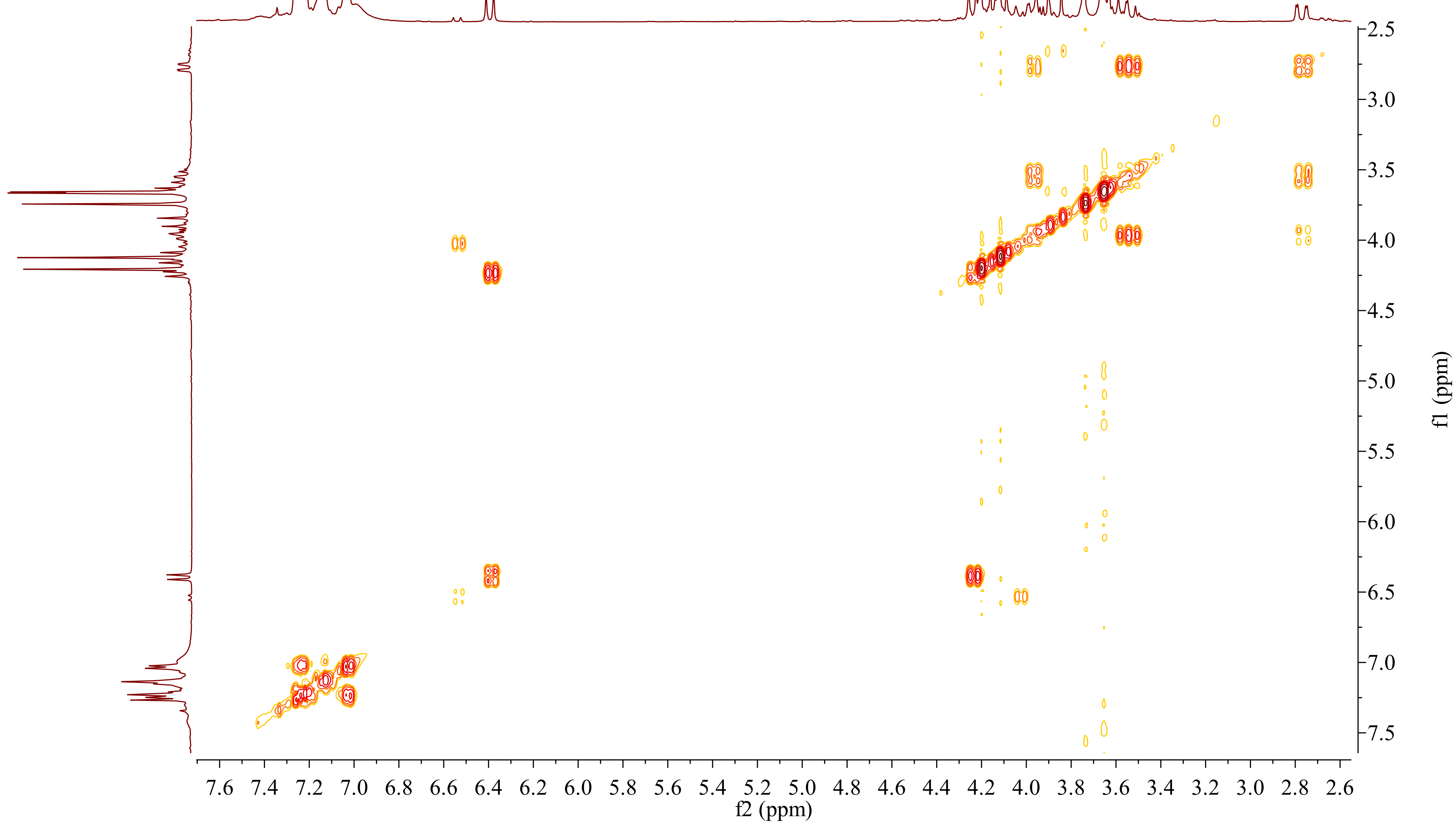

S2.21. HMBC spectrum of compounds **4a** and **4b**

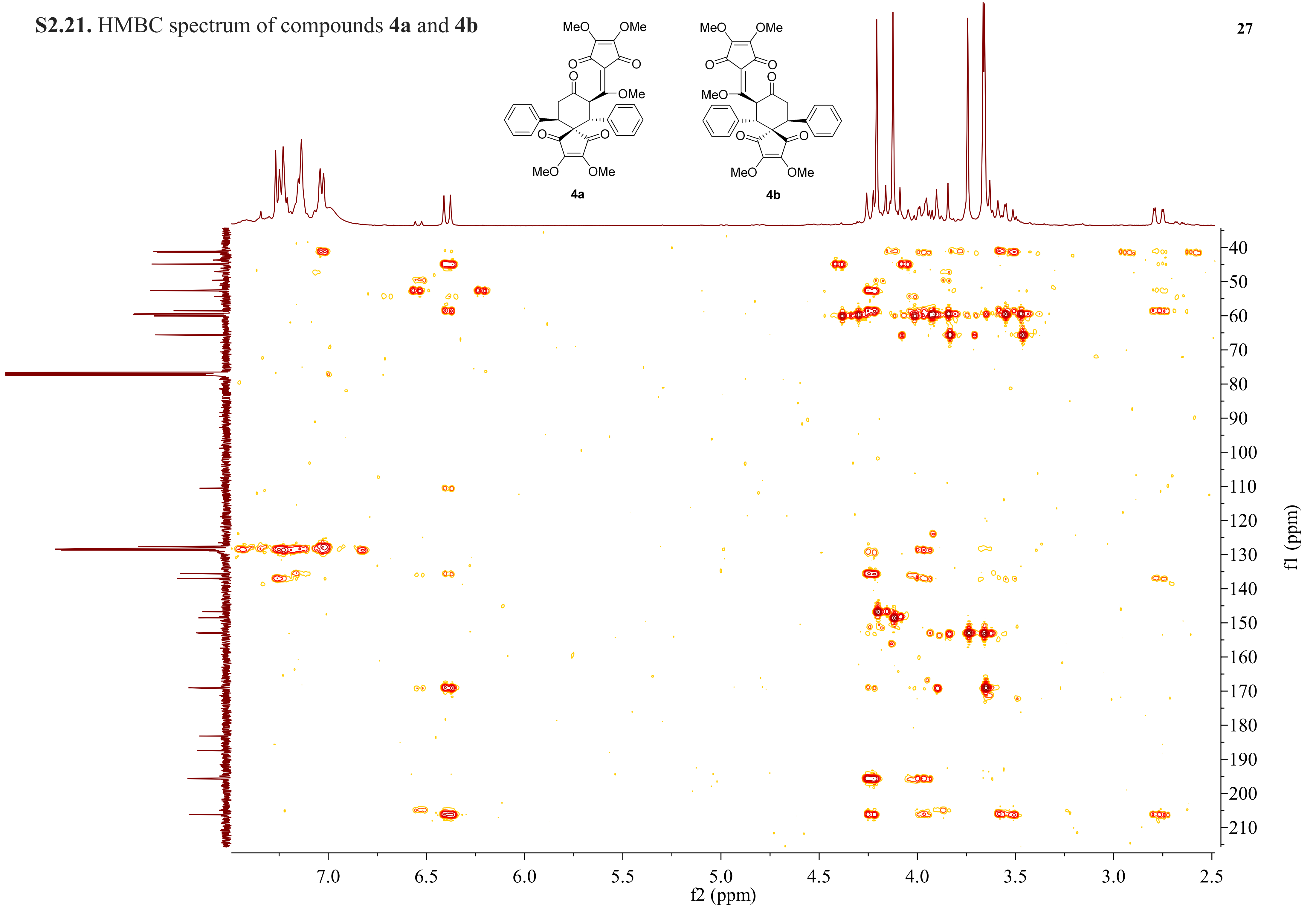

S2.22. NOESY spectrum of compounds **4a** and **4b**

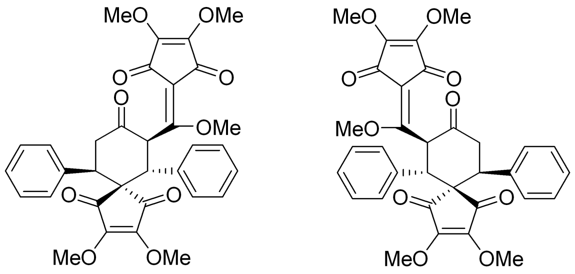

4a **4b**

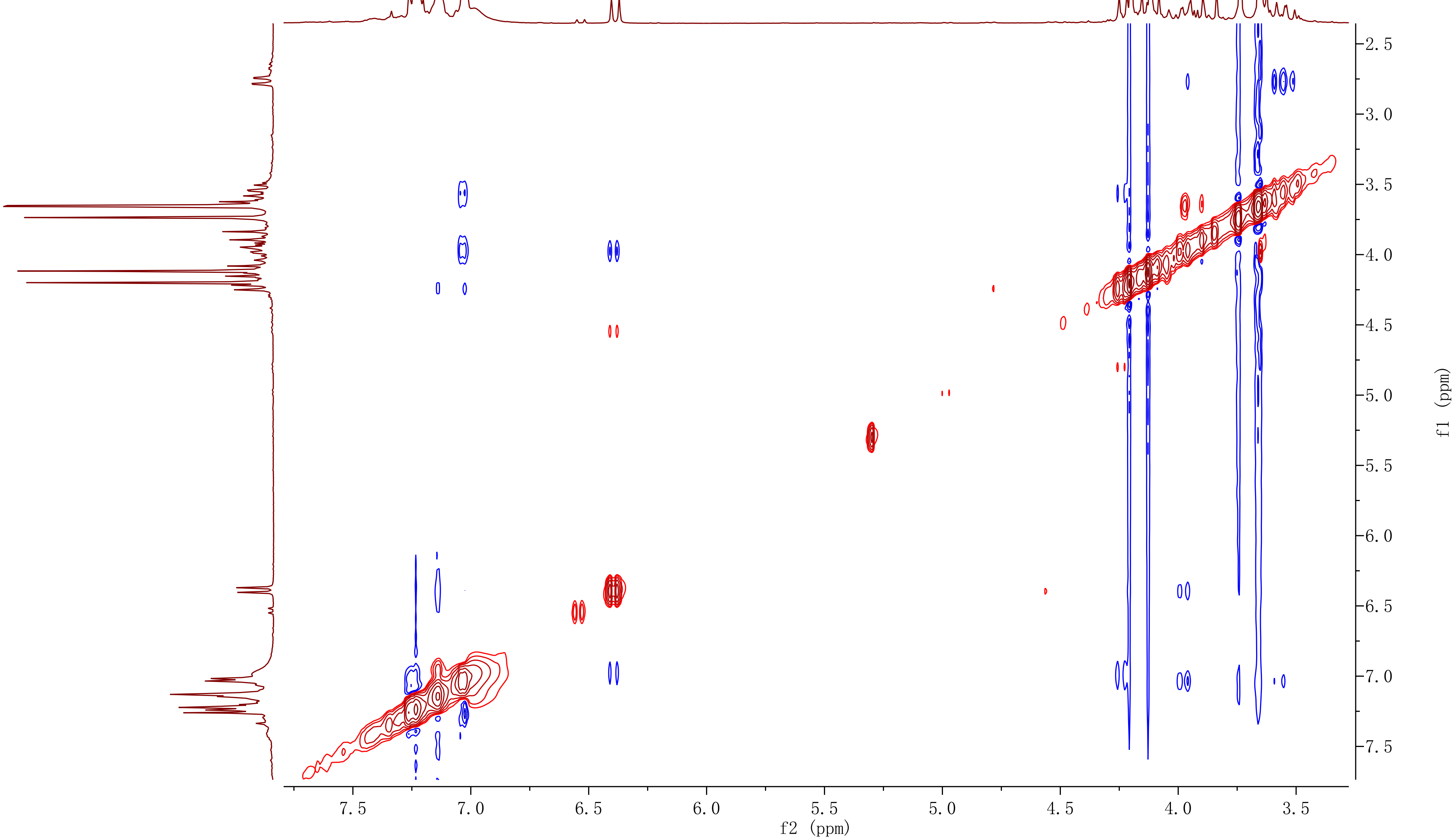

S2.23. ^{1}H NMR spectrum of compound **5**

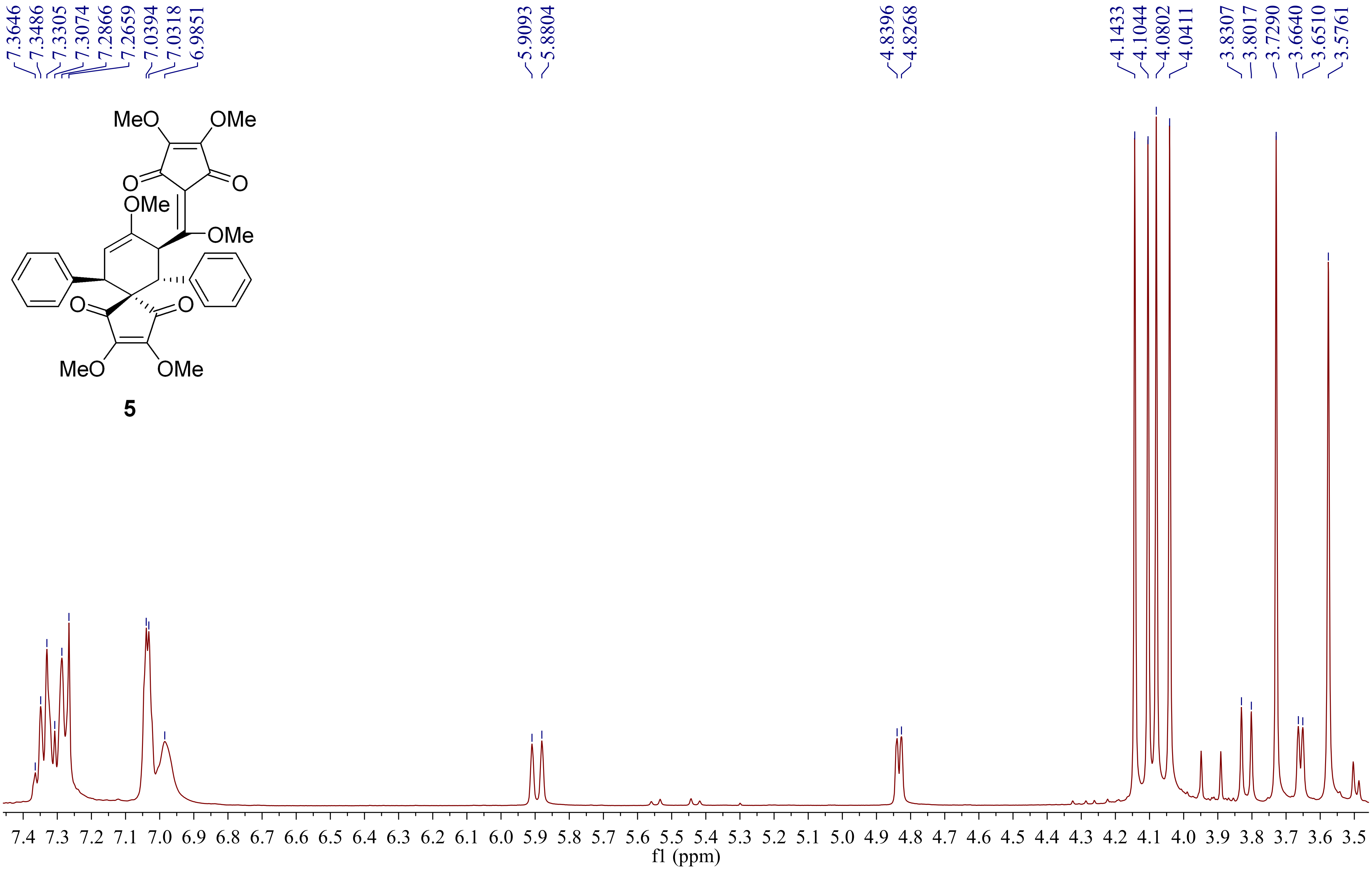

S2.24. ^{13}C NMR and DEPT spectra of compound **5**

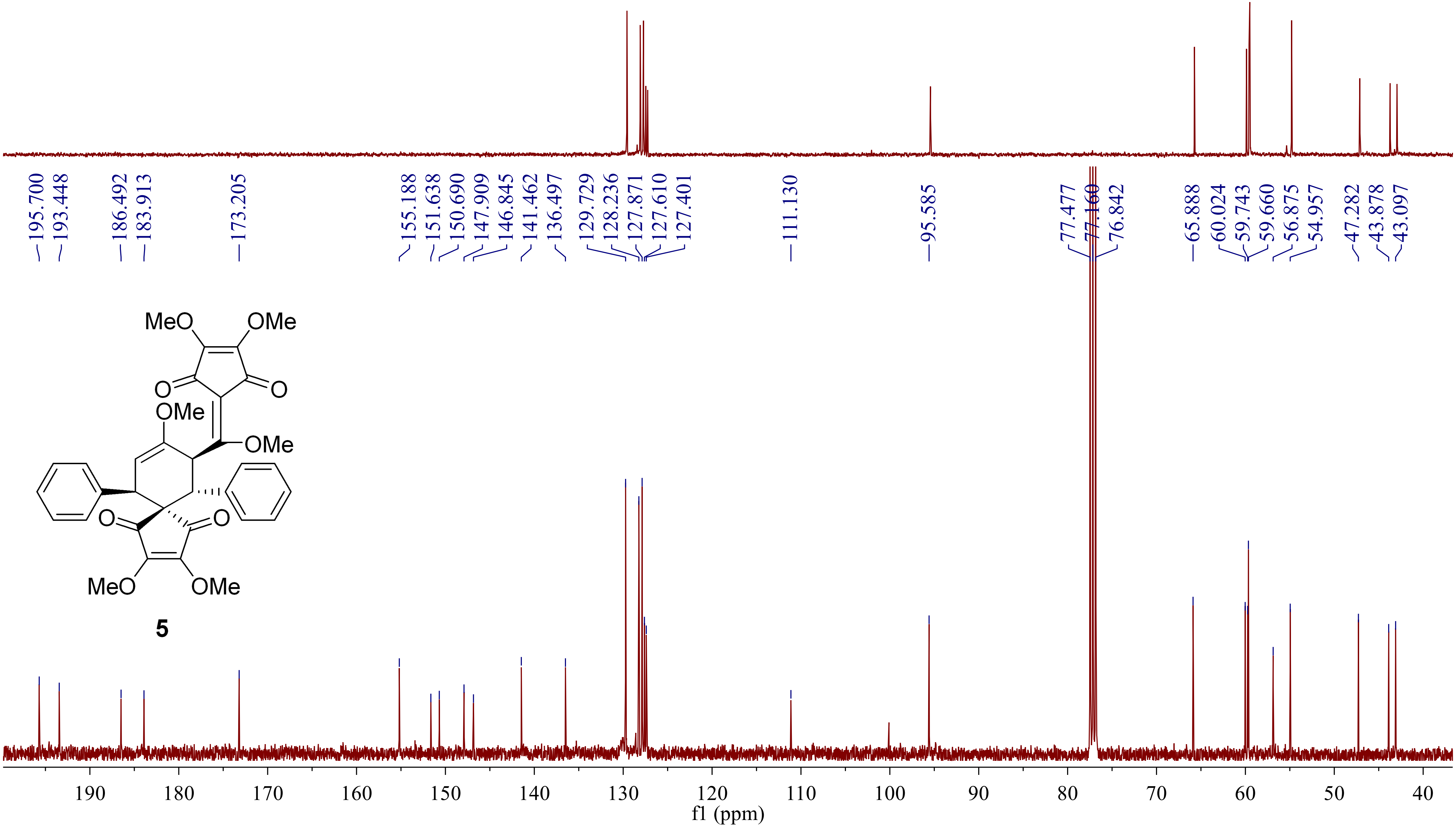

S2.25. ^{1}H NMR spectrum of compound **6**

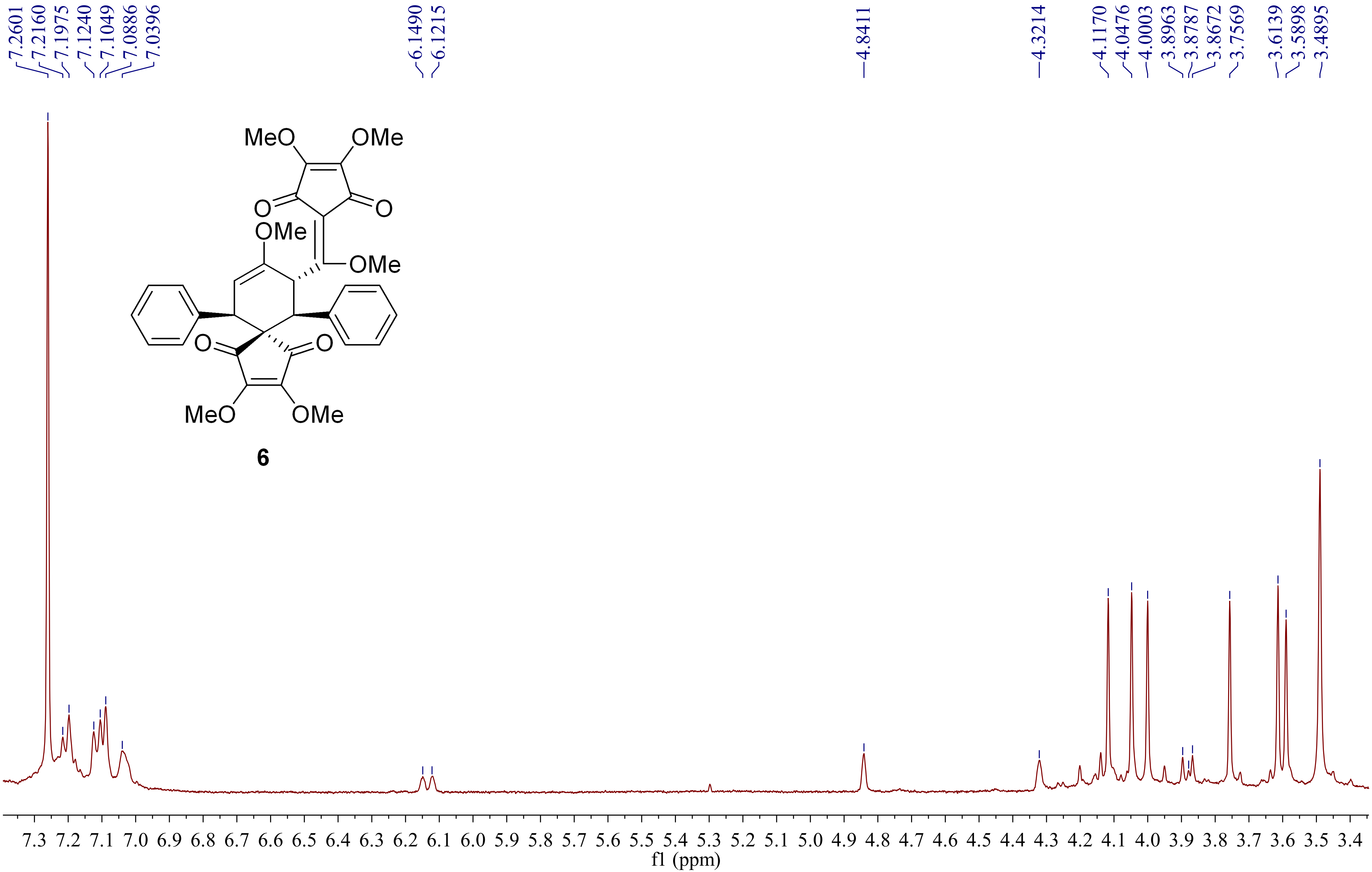

S2.26. ^{13}C NMR and DEPT spectra of compound **6**

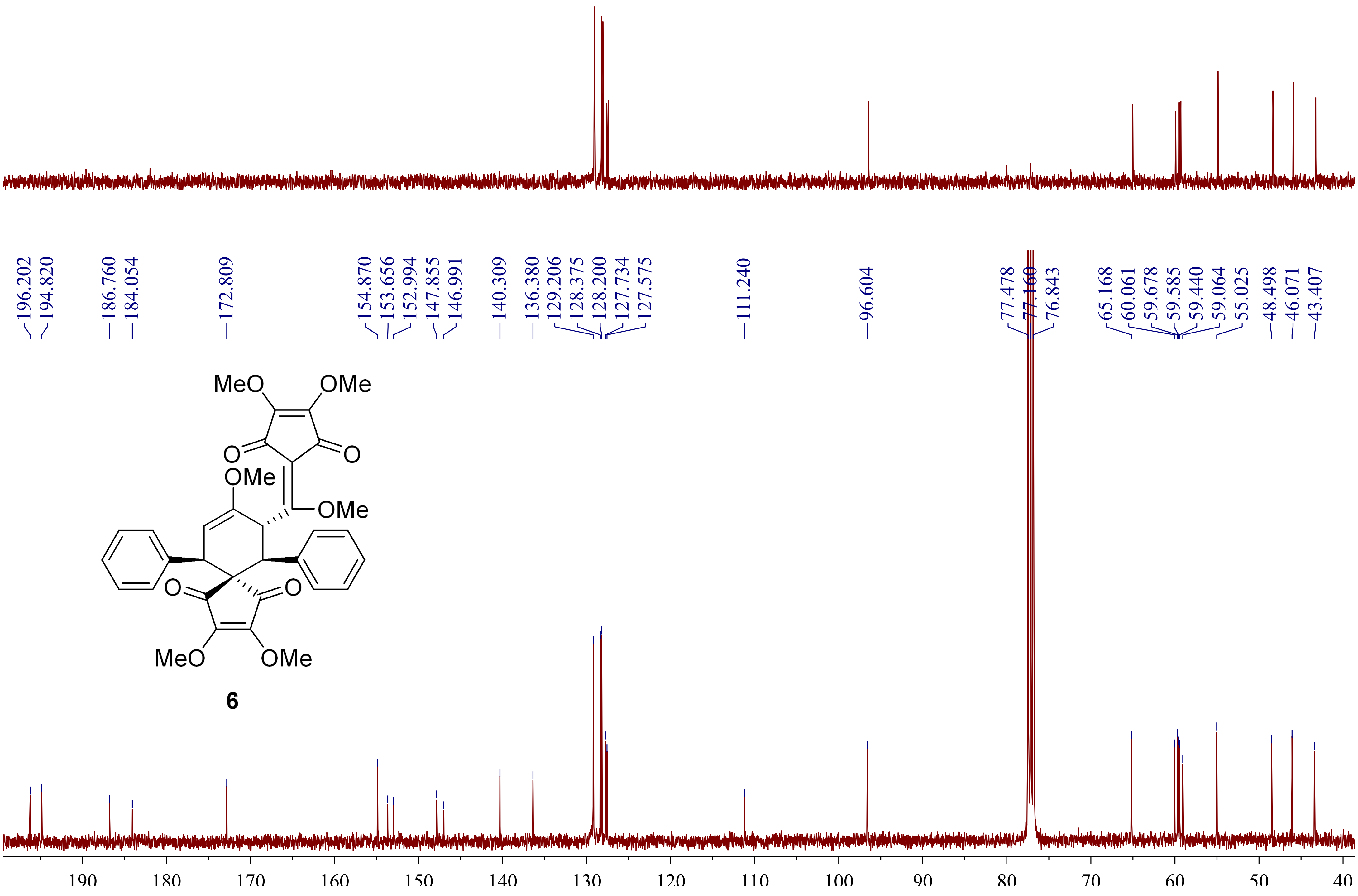

S2.27. ^{1}H NMR spectrum of compound **7**

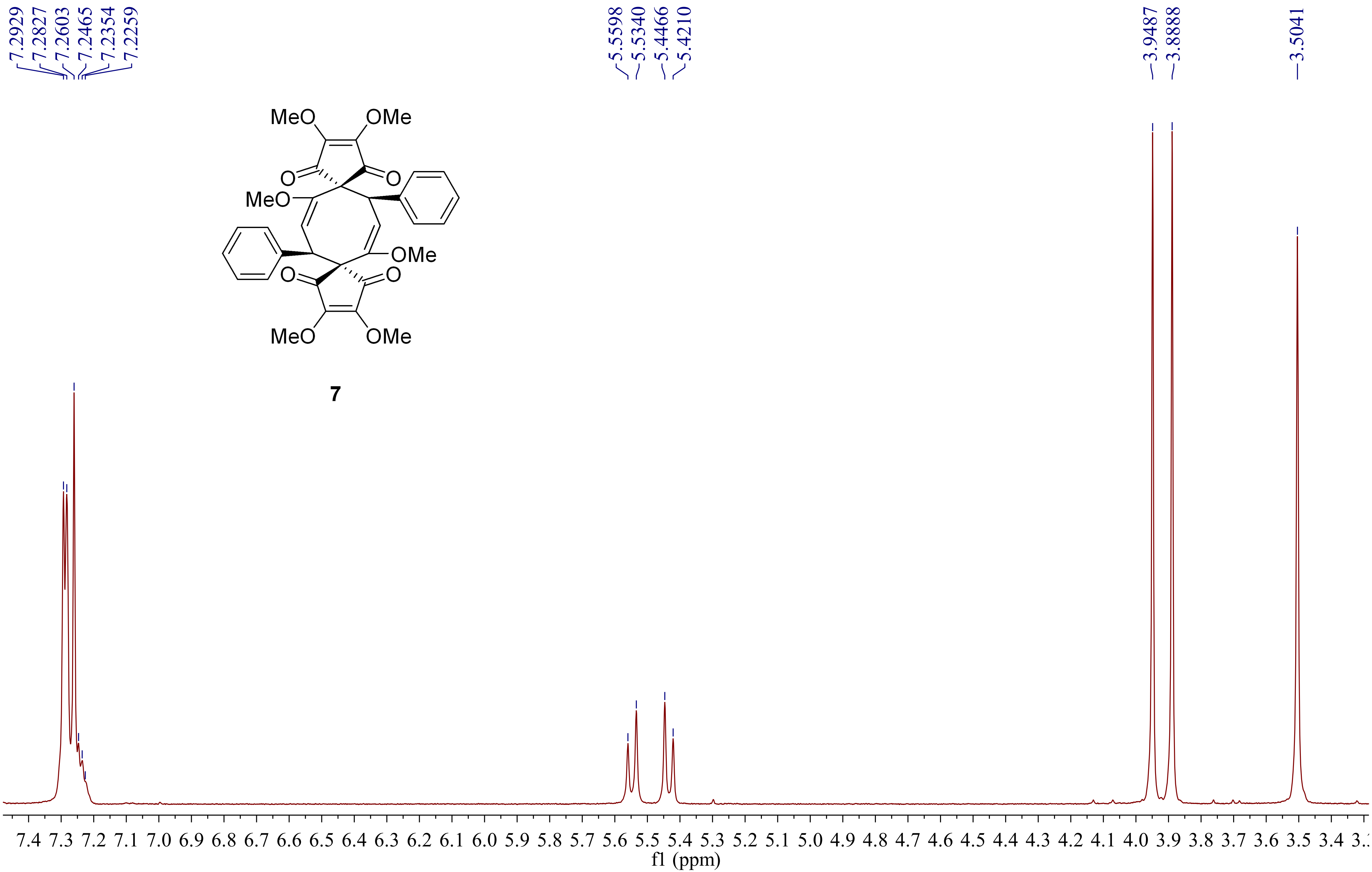

S2.28. ¹³C NMR and DEPT spectra of compound **7**

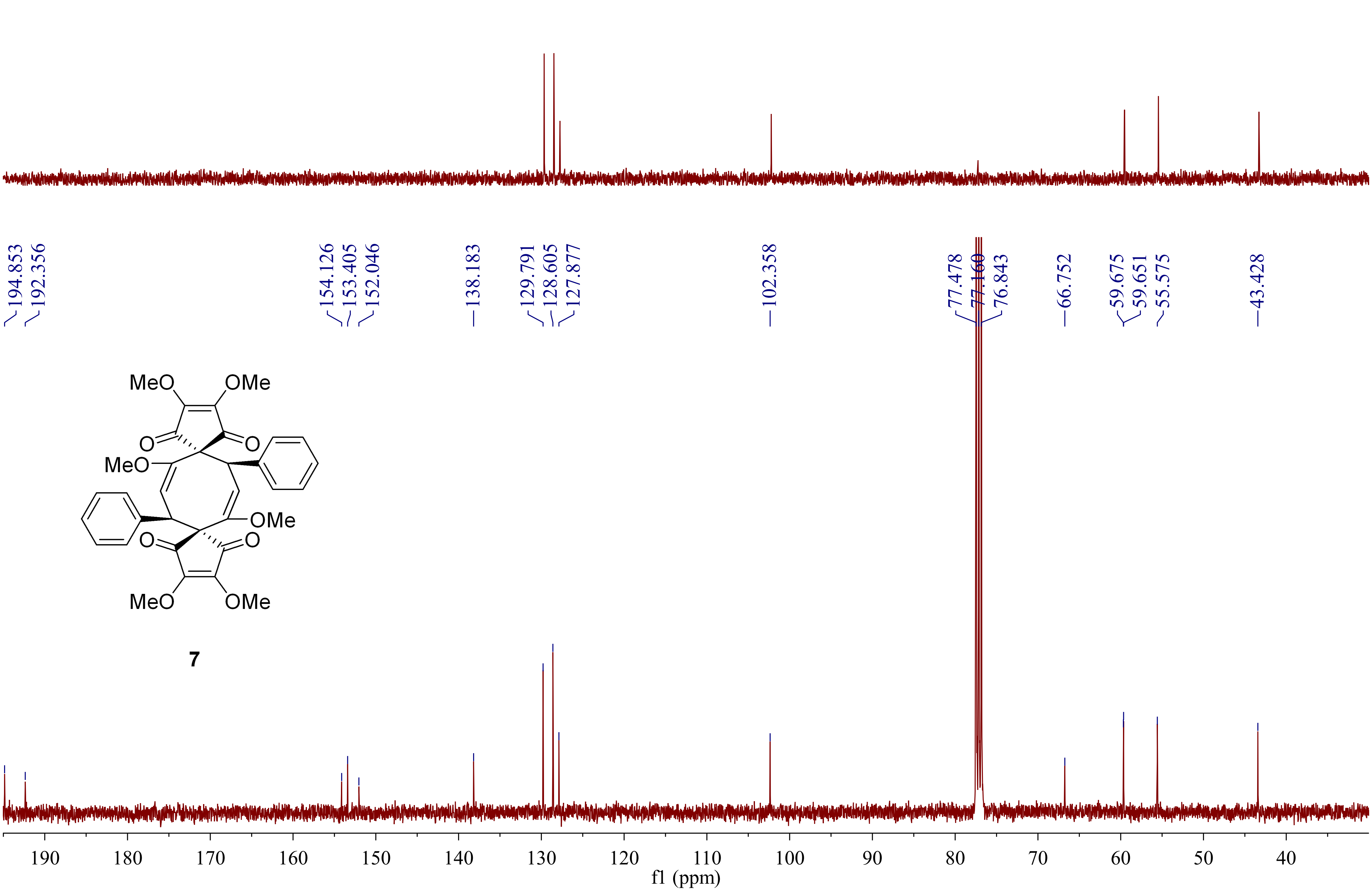

S2.29. ^{1}H NMR spectrum of compound **8**

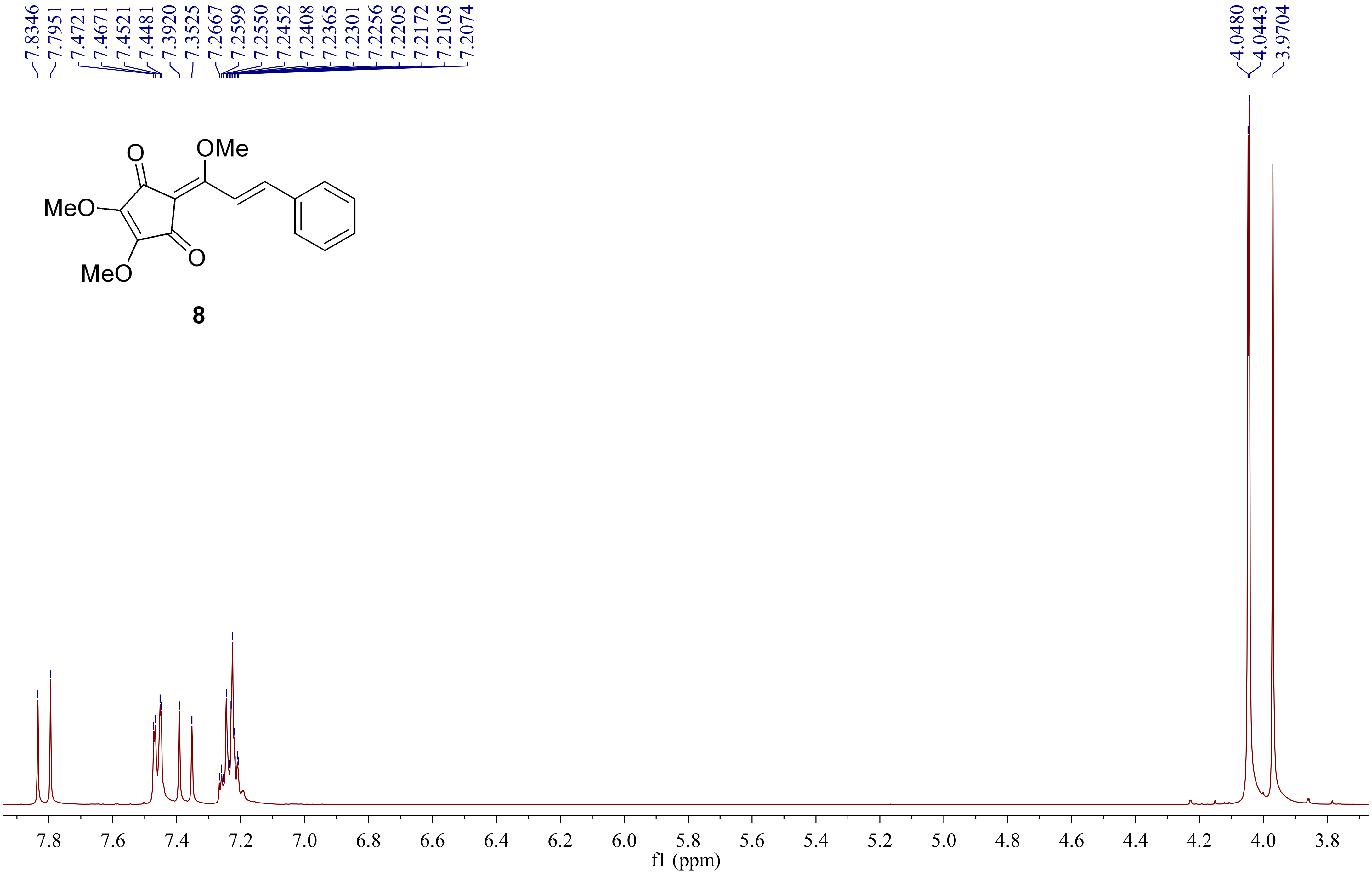

S2.30. ^{13}C NMR and DEPT spectra of compound **8**

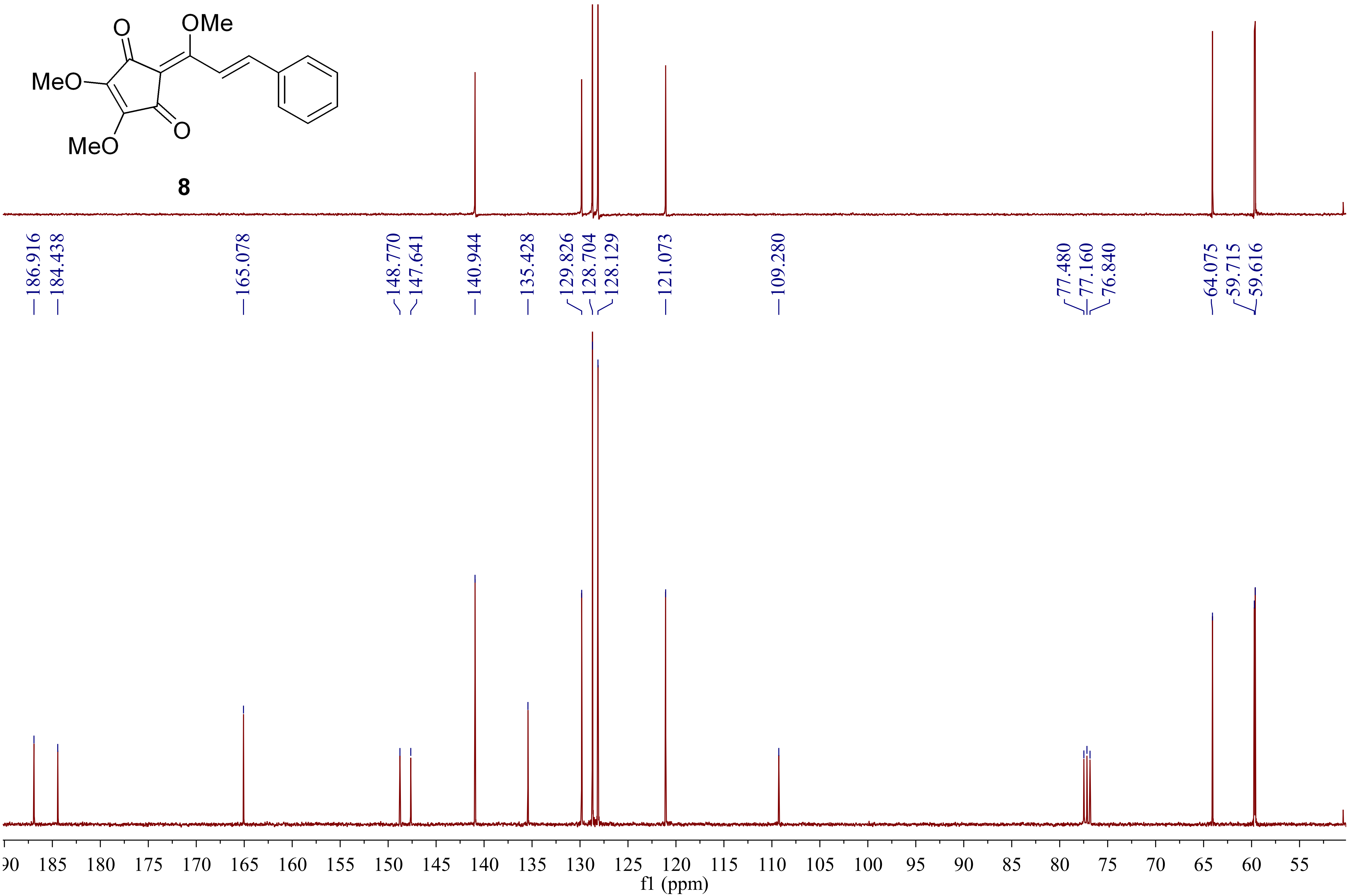

S2.31. ^{1}H NMR spectrum of compounds **9a** and **9b**

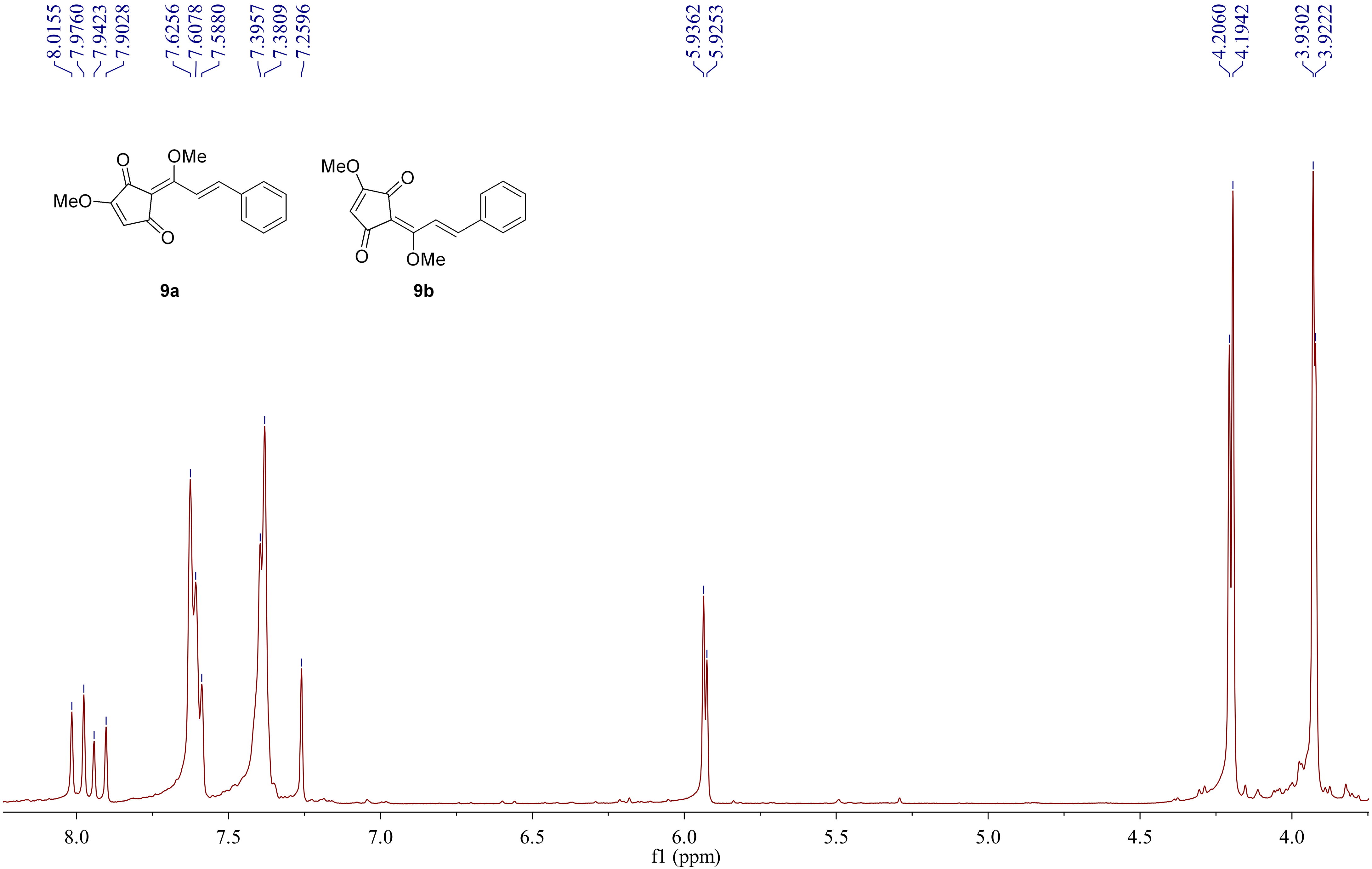

S2.32. ^{13}C NMR and DEPT spectra of compounds **9a** and **9b**

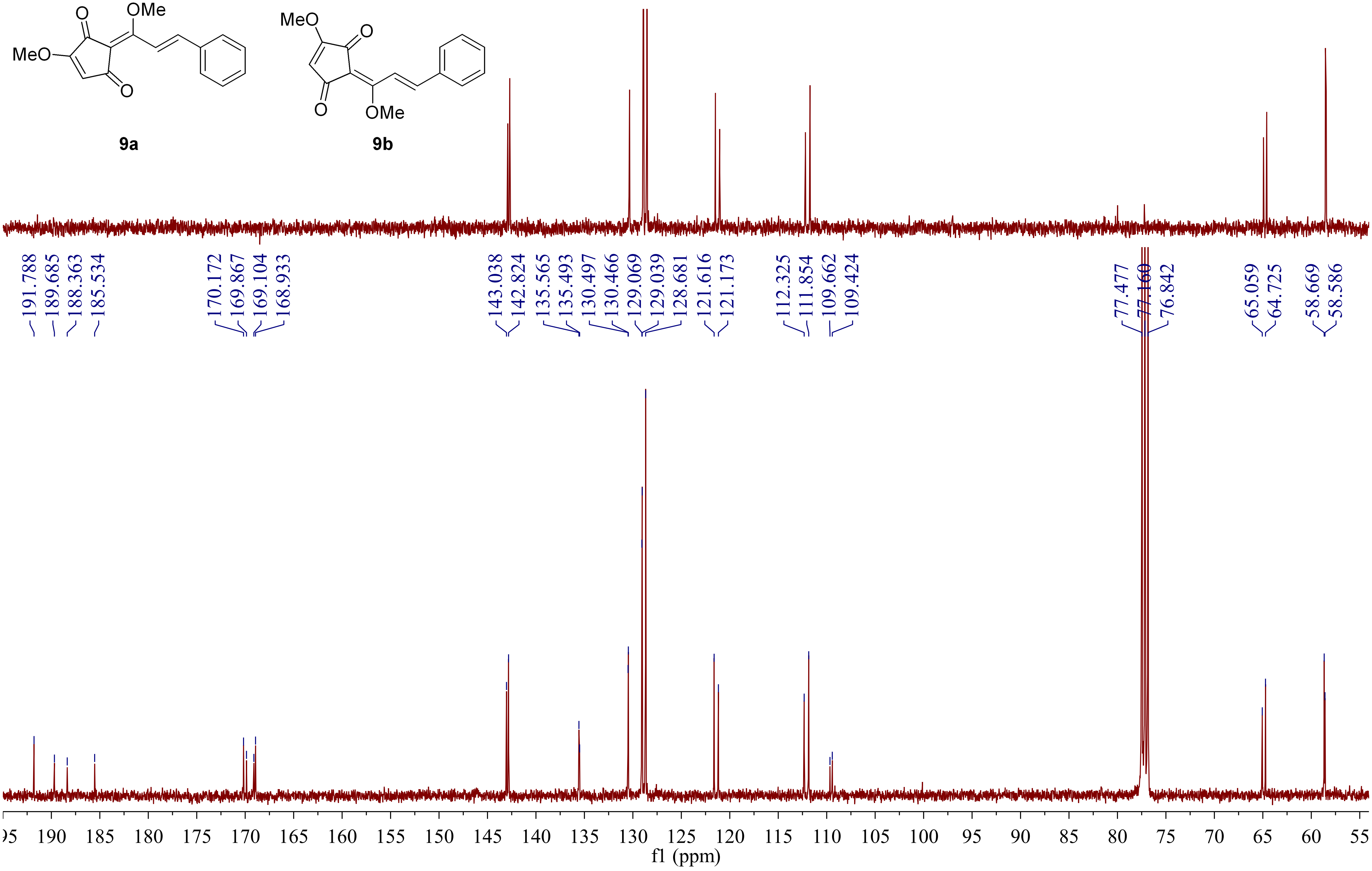

S3.1. MS data of compound **1**

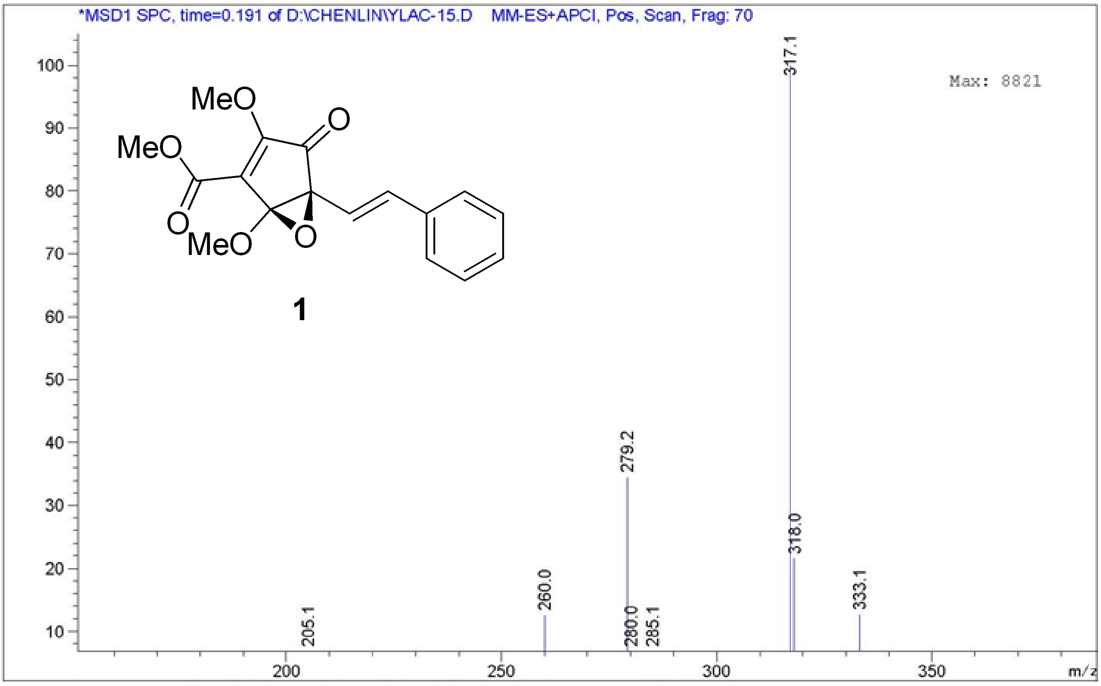

S3.2. HR-ESI-MS data of compound **1**

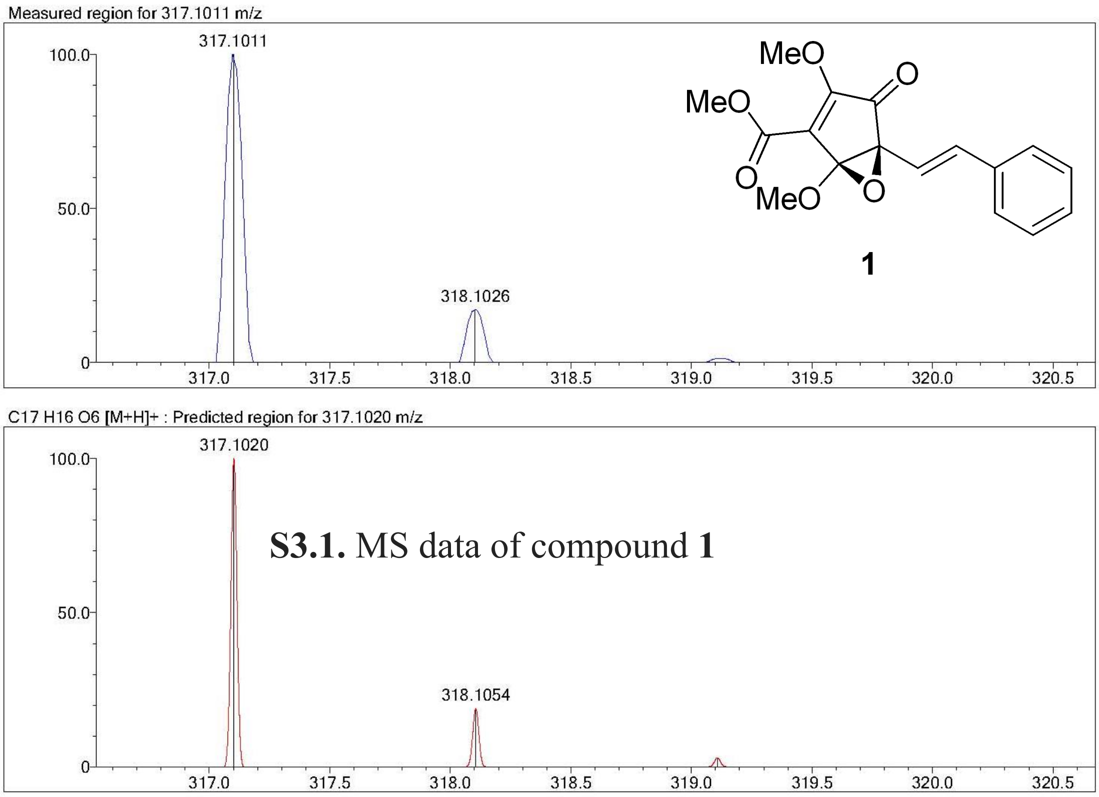

Rank	Score	Formula (M)	Ion	Meas. m/z	Pred. m/z	Df. (mDa)	Df. (ppm)	Iso	DBE
4	74.42	C17 H16 O6	[M+H]+	317.1011	317.1020	-0.9	-2.84	78.01	10.0

S3.3. MS data of compound **2**

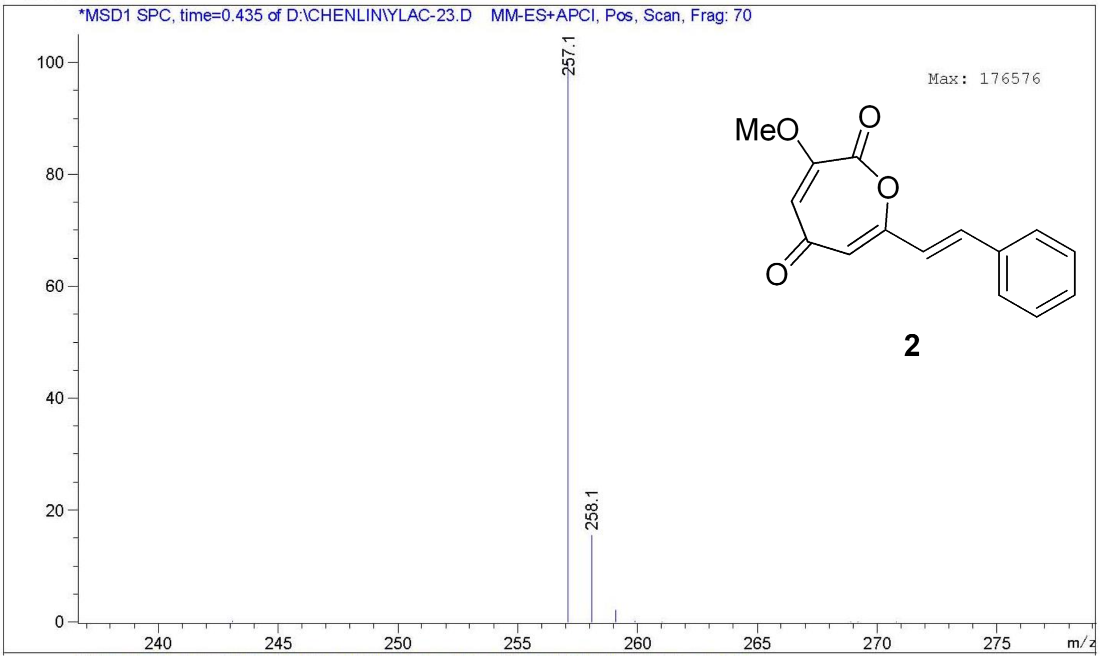

S3.4. HR-ESI-MS data of compound **2**

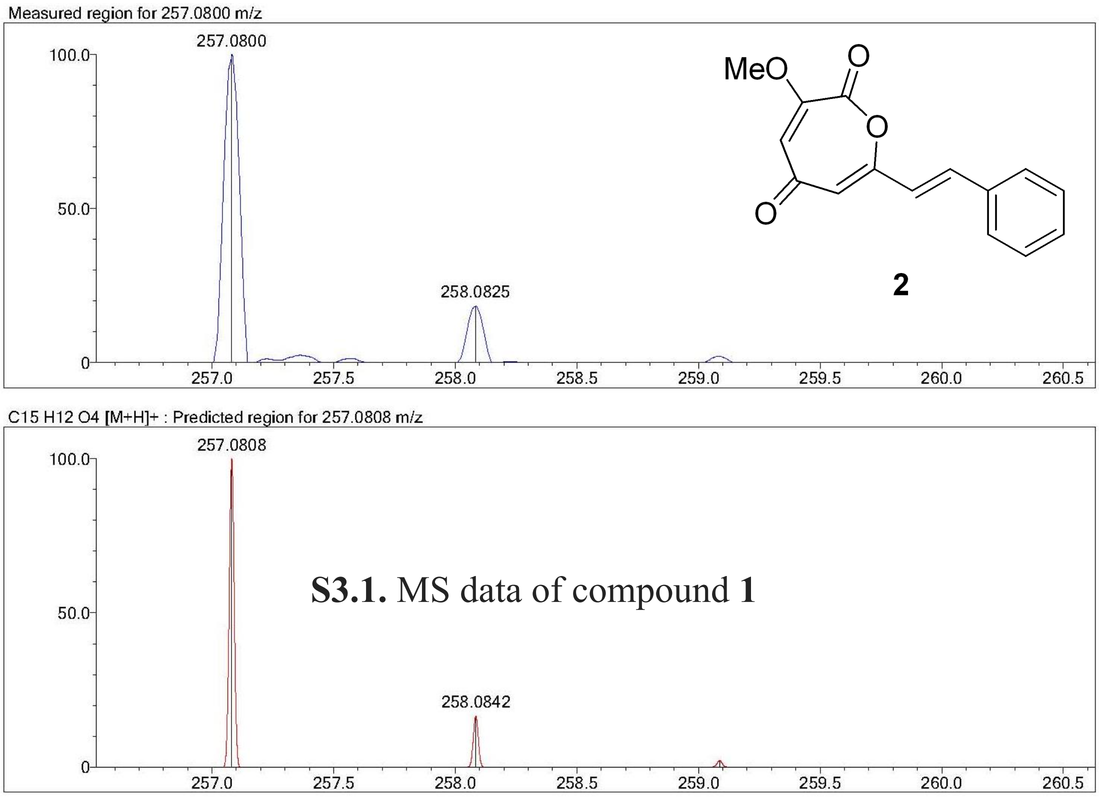

Rank	Score	Formula (M)	Ion	Meas. m/z	Pred. m/z	Df. (mDa)	Df. (ppm)	Iso	DBE
2	68.62	C15 H12 O4	[M+H]+	257.0800	257.0808	-0.8	-3.11	72.44	10.0

S3.5. MS data of compound **3**

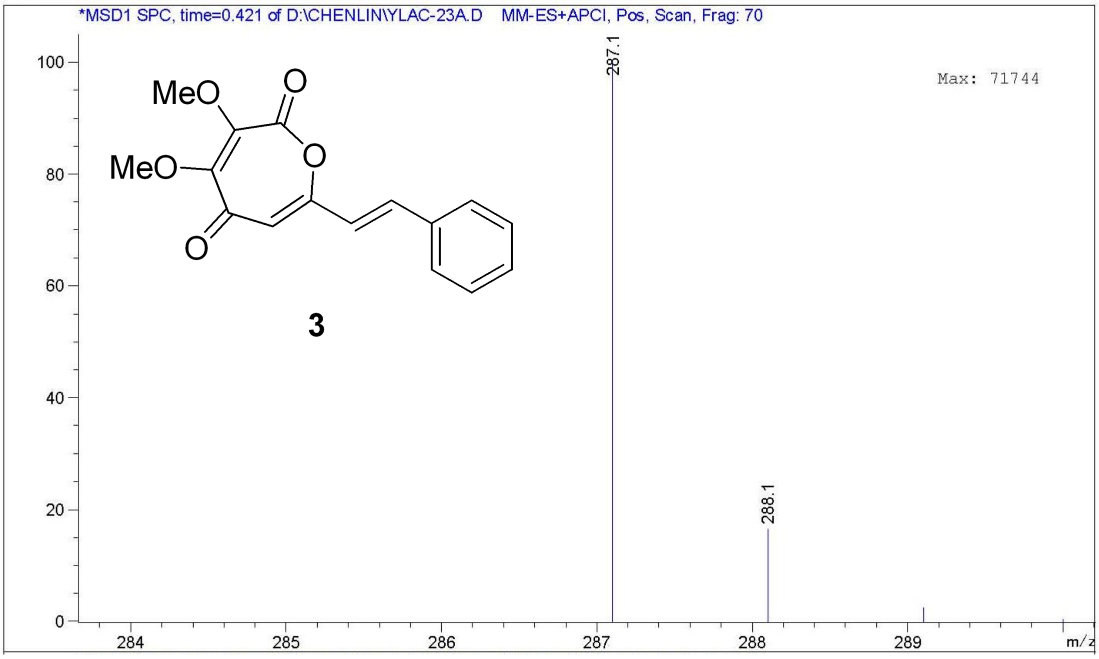

S3.6. HR-ESI-MS data of compound **3**

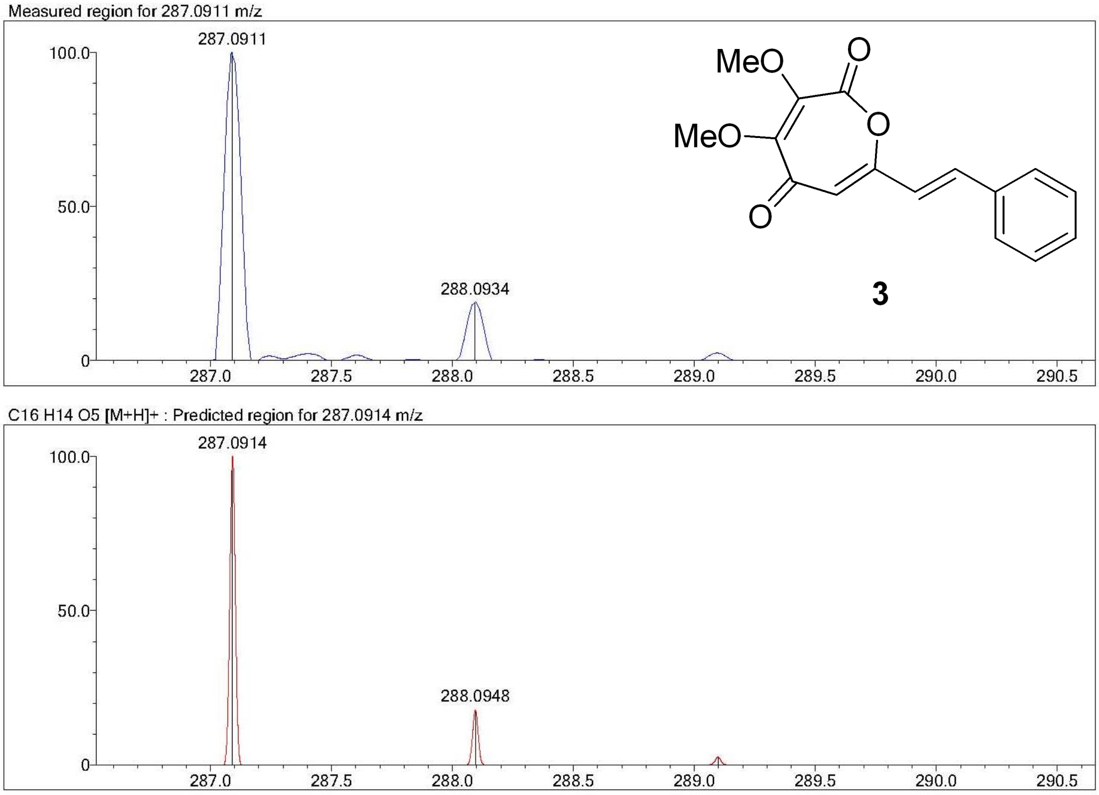

Rank	Score	Formula (M)	Ion	Meas. m/z	Pred. m/z	Df. (mDa)	Df. (ppm)	Iso	DBE
1	75.35	C16 H14 O5	[M+H]+	287.0911	287.0914	-0.3	-1.04	75.43	10.0

S3.7. MS data of compounds **4a** and **4b**

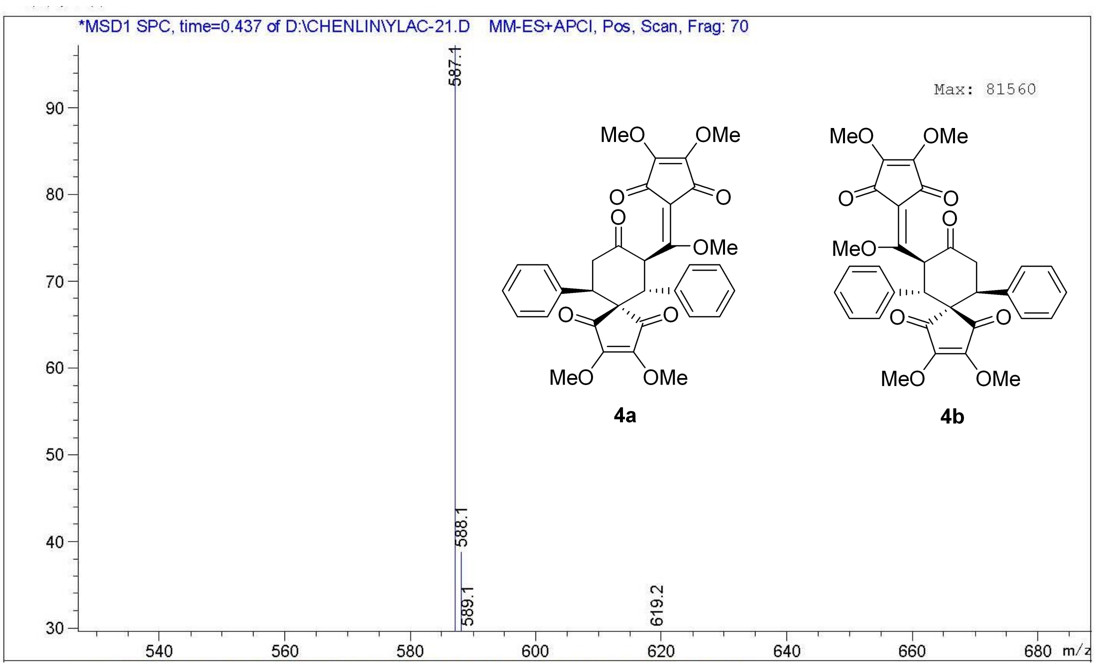

S3.8. HR-ESI-MS data of compounds **4a** and **4b**

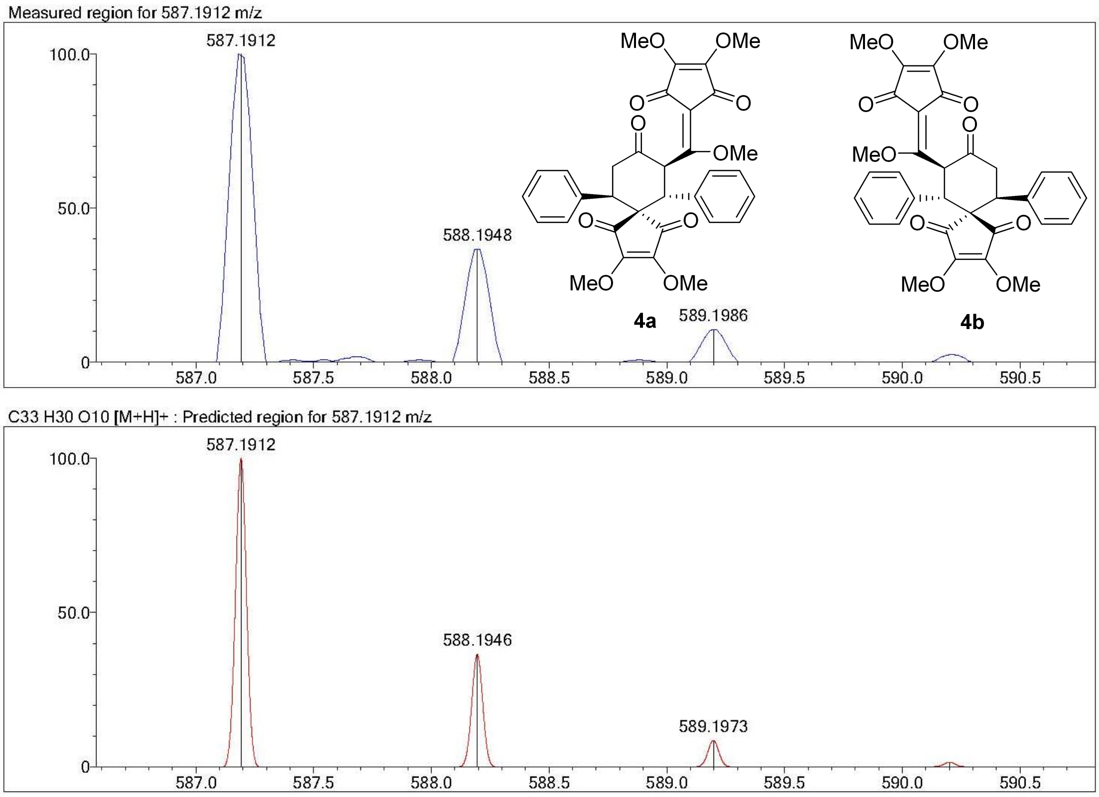

Rank	Score	Formula (M)	Ion	Meas. m/z	Pred. m/z	Df. (mDa)	Df. (ppm)	Iso	DBE
1	93.82	C33 H30 O10	[M+H]+	587.1912	587.1912	0.0	0.00	93.82	19.0

S3.9. IR (KBr disc) spectrum of compound **1**

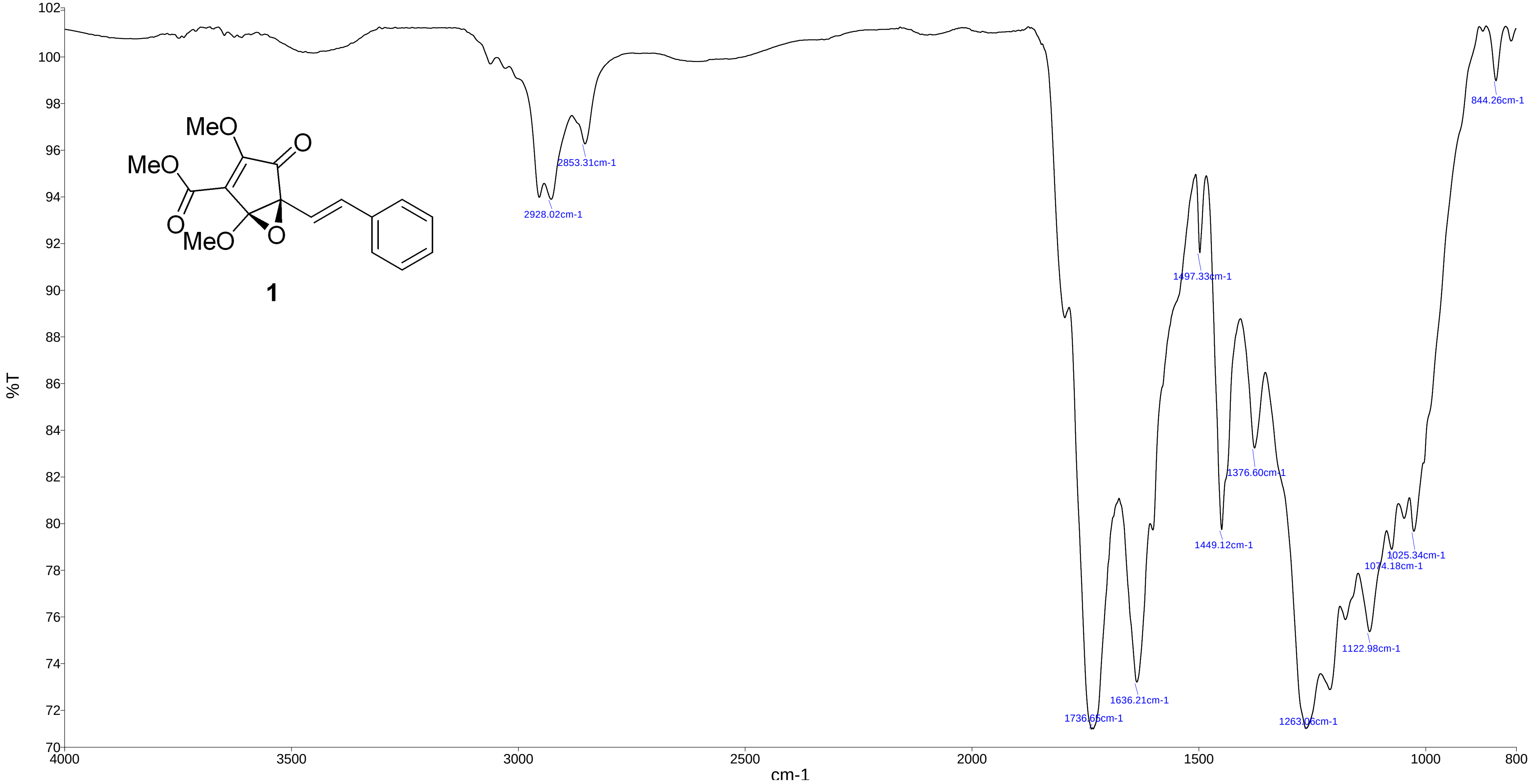

S3.10. IR (KBr disc) spectrum of compound **2**

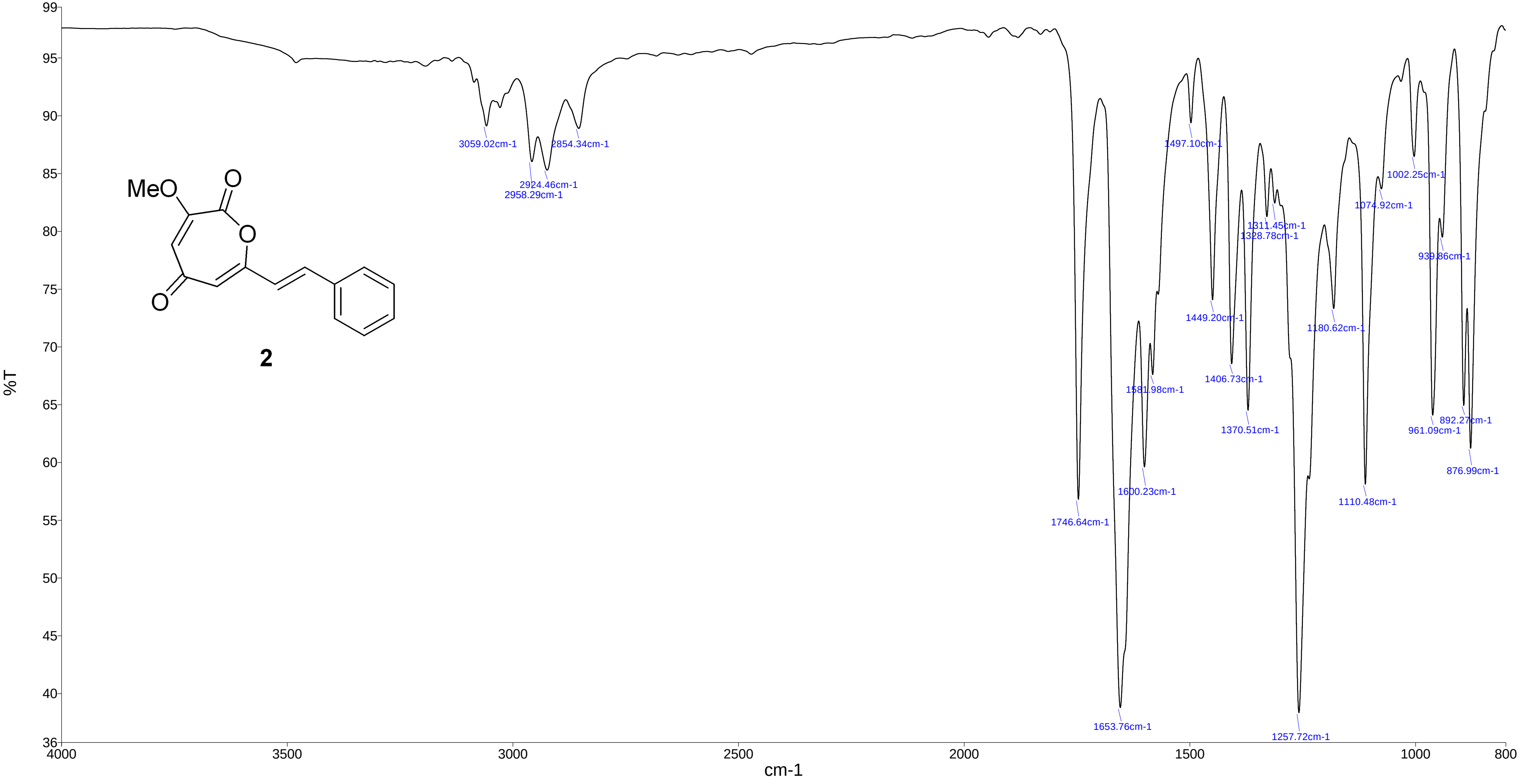

S3.11. IR (KBr disc) spectrum of compound 3

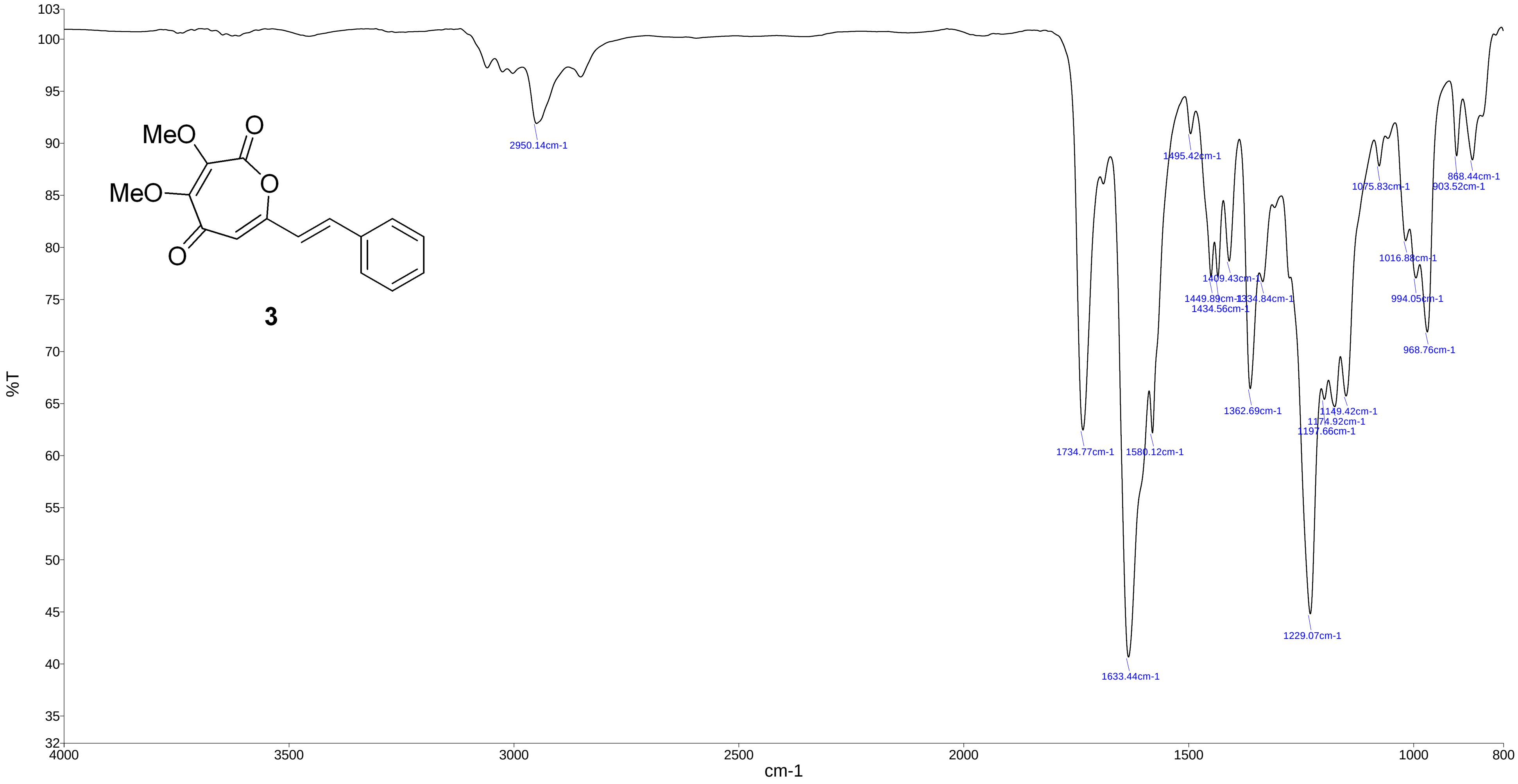

S3.12. IR (KBr disc) spectrum of compounds **4a** and **4b**

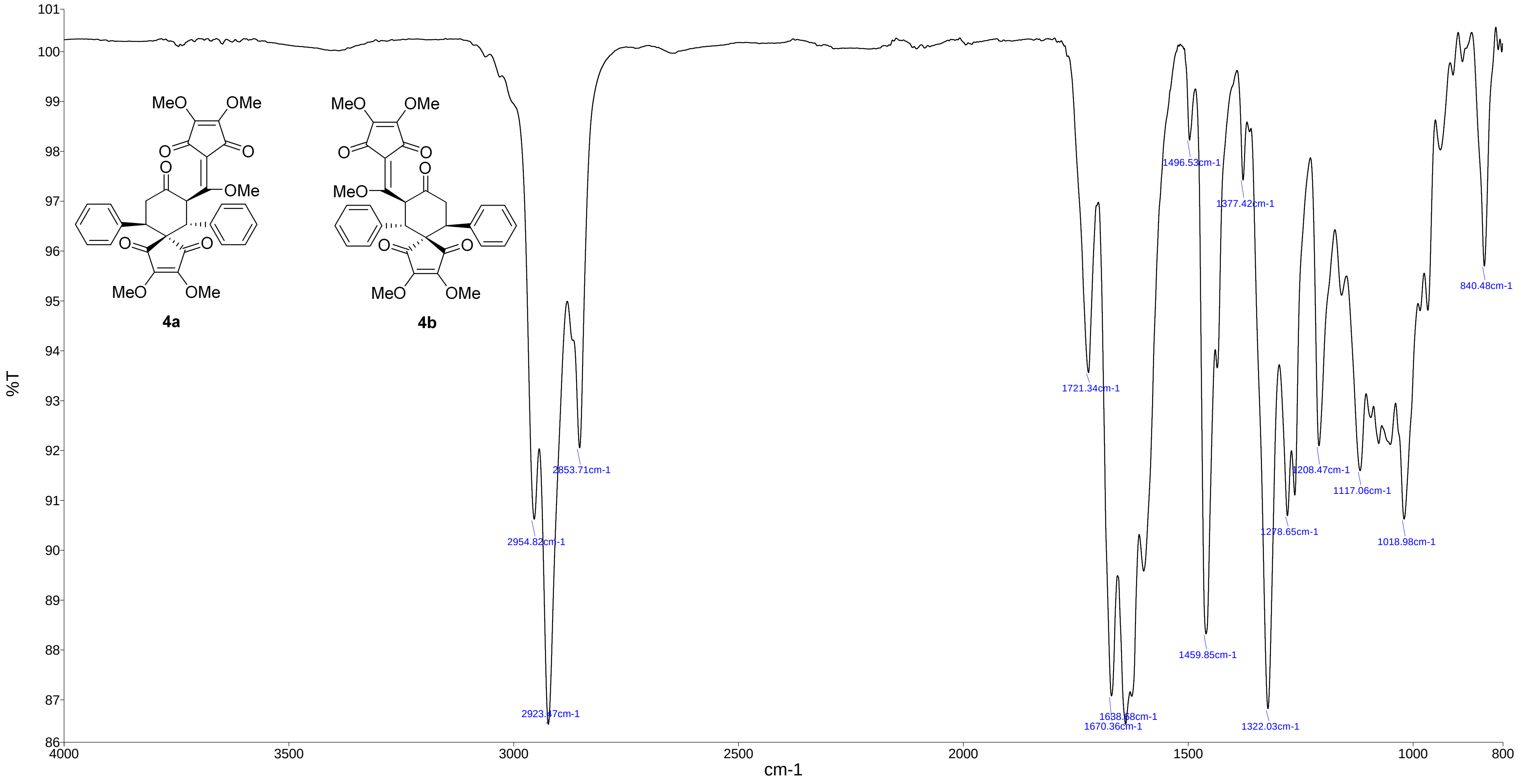

S4. ECD computational details.

In general, conformational analyses were carried out via random searching in the Sybyl-X 2.0 using the MMFF94S force field with an energy cutoff of 2.5 kcal/mol [1]. Due to the rigid skeleton and the limitations of NOESY correlations, the results showed only one lowest energy conformer for *RRR*-**4** whose relative energy within 2.5 kcal/mol. Subsequently, the conformer was re-optimized using DFT at the B3LYP/6-31+G(d) level in gas phase by the GAUSSIAN 09 program [2]. The energies, oscillator strengths, and rotational strengths (velocity) of the first 60 electronic excitations were calculated using the TDDFT methodology at the B3LYP/6-311++G(d,p) level in vacuum. The ECD spectra were simulated by the overlapping Gaussian function (half the bandwidth at 1/e peak height, σ = 0.22 eV) [3]. Theoretical ECD spectrum of the corresponding enantiomer was obtained by directly inverse of the ECD spectrum of the calculated model molecule. By comparison of the calculated ECD spectra with the experimental ones, the absolute configuration of **4a** and **4b** was resolved.

[1]. Sybyl Software, version X 2.0; Tripos Associates Inc.: St. Louis, MO, 2013.

[2]. Frisch, M. J.; Trucks, G. W.; Schlegel, H. B.; Scuseria, G. E.; Robb, M. A.; Cheeseman, J. R.; Scalmani, G.; Barone, V.; Mennucci, B.; Petersson, G. A.; Nakatsuji, H.; Caricato, M.; Li, X.; Hratchian, H. P.; Izmaylov, A. F.; Bloino, J.; Zheng, G.; Sonnenberg, J. L.; Hada, M.; Ehara, M.; Toyota, K.; Fukuda, R.; Hasegawa, J.; Ishida, M.; Nakajima, T.; Honda, Y.; Kitao, O.; Nakai, H.; Vreven, T.; Montgomery, Jr., J. A.; Peralta, J. E.; Ogliaro, F.; Bearpark, M.; Heyd, J. J.; Brothers, E.; Kudin, K. N.; Staroverov, V. N.; Kobayashi, R.; Normand, J.; Raghavachari, K.; Rendell, A.; Burant, J. C.; Iyengar, S. S.; Tomasi, J.; Cossi, M.; Rega, N.; Millam, J. M.; Klene, M.; Knox, J. E.; Cross, J. B.; Bakken, V.; Adamo, C.; Jaramillo, J.; Gomperts, R.; Stratmann, R. E.; Yazyev, O.; Austin, A. J.; Cammi, R.; Pomelli, C.; Ochterski, J. W.; Martin, R. L.; Morokuma, K.; Zakrzewski, V. G.; Voth, G. A.; Salvador, P.; Dannenberg, J. J.; Dapprich, S.; Daniels, A. D.; Farkas, Ö.; Foresman, J. B.; Ortiz, J. V.; Cioslowski, J.; Fox, D. J. Gaussian 09, Rev. C 01; Gaussian, Inc., Wallingford CT, 2009.

[3]. Stephens, P. J.; Harada, N. ECD cotton effect approximated by the Gaussian curve and other methods. *Chirality* **2010**, *22*, 229–233.

S4.1. The stable conformer of 1C of *RRR*-**4**.

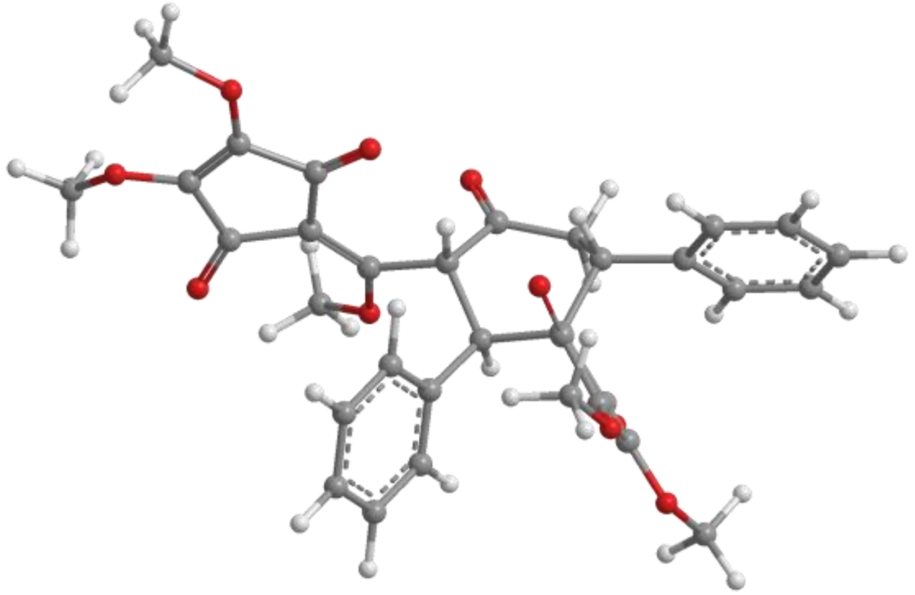

E(RB3LYP) -2028.25581948

S4.2. Important thermodynamic parameters (a.u.) of the optimized **4** at the B3LYP/6-31G(d) level in the gas phase.

Conformer	*E*	*E'=E+ZPE*	*H*	*G*
1C of *RRR*-**4**	-2027.157662	-2027.198934	-2027.156718	-2027.277369

E, *E'*, *H*, *G*: total energy, total energy with zero point energy (ZPE), enthalpy, and Gibbs free

S4.3. Excitation energies, oscillator strengths, and rotatory strengths in the ECD spectra of 1C of *RRR*-**4**

Excited State	Excitation Energies (eV)	Wavelength (nm)	Oscillator strengths	Rotatory strength*	Excited State	Excitation Energies (eV)	Wavelength (nm)	Oscillator strengths	Rotatory strength*
1	3.2312	383.71	0.0027	27.1267	31	4.7878	258.96	0.0366	-39.3049
2	3.2644	379.80	0.0102	-18.1247	32	4.8536	255.45	0.0374	-3.0563
3	3.2781	378.22	0.0088	-5.6629	33	4.8896	253.57	0.1109	61.8589
4	3.4477	359.62	0.0009	14.6460	34	4.9163	252.19	0.0224	2.5333
5	3.5114	353.09	0.0046	-6.7515	35	5.0009	247.92	0.0020	0.6639
6	3.5310	351.13	0.0003	-3.0062	36	5.0311	246.44	0.0009	-0.3798

7	3.7548	330.20	0.0689	17.4277	37	5.0398	246.01	0.0042	-0.7537
8	3.7651	329.30	0.0301	-17.0925	38	5.0616	244.95	0.0010	-0.7110
9	3.8532	321.77	0.0221	7.3104	39	5.1105	242.61	0.0030	5.3365
10	3.9019	317.75	0.0035	2.3127	40	5.1267	241.84	0.0954	12.4478
11	3.9407	314.62	0.0018	-2.1319	41	5.1651	240.04	0.0064	2.5326
12	3.9598	313.11	0.0011	-0.5389	42	5.1955	238.64	0.0008	-0.3363
13	3.9647	312.72	0.0047	4.1934	43	5.2292	237.10	0.0026	0.3520
14	4.0036	309.68	0.0031	-0.4173	44	5.2477	236.27	0.0151	16.8677
15	4.0501	306.13	0.0057	-5.4291	45	5.2657	235.45	0.0062	5.7470
16	4.1534	298.51	0.3281	10.7221	46	5.3035	233.78	0.0126	4.5481
17	4.2526	291.55	0.0108	-25.9453	47	5.3083	233.56	0.0005	-1.3998
18	4.2607	291.00	0.0645	20.3618	48	5.3412	232.13	0.0119	-2.0134
19	4.3348	286.02	0.0100	33.2084	49	5.3559	231.49	0.0007	0.5973
20	4.3460	285.28	0.0016	-1.5933	50	5.3760	230.62	0.0165	-6.5530
21	4.3531	284.82	0.0043	0.1093	51	5.4073	229.29	0.0219	-8.6573
22	4.3602	284.35	0.0049	-9.6630	52	5.4368	228.05	0.0014	-0.1521
23	4.4134	280.92	0.0038	0.3650	53	5.4551	227.28	0.0145	-10.6730
24	4.4945	275.86	0.0003	0.0160	54	5.4669	226.79	0.0486	-22.6383
25	4.5019	275.40	0.0004	-0.0515	55	5.5011	225.38	0.0049	3.4005
26	4.5651	271.59	0.0033	-0.8720	56	5.5168	224.74	0.0371	1.3825
27	4.5740	271.06	0.0053	-1.2824	57	5.5329	224.08	0.0859	38.8532
28	4.6582	266.16	0.0005	-0.1776	58	5.5495	223.42	0.0099	-2.3337
29	4.7116	263.15	0.0010	2.3118	59	5.5699	222.60	0.0069	-1.4135
30	4.7189	262.74	0.0034	-0.3018	60	5.5905	221.78	0.0052	-0.1310

* R(velocity) 10**-40 erg-esu-cm
